# MHY@MO@PPP inhibits ferroptosis positive feedback and delays the acute kidney injury-to-chronic kidney disease transition by remodeling the Fe^2+^-mTORC1-GPX4 axis

**DOI:** 10.1186/s12951-026-04918-w

**Published:** 2026-08-20

**Authors:** Chenhao Li, Chao Zhang, Chuanqi Zhang, Shiyu Duan, Jiali Zhang, Hongyue Wang

**Affiliations:** 1 Department of Nephrology, Lequn Branch, The First Hospital of Jilin University, No. 3302 Jilin Road, Erdao District, Changchun, 130031 Jilin Province China; 2 Ultrasound Diagnostic Center, The First Hospital of Jilin University, Changchun, 130031 China

**Keywords:** Ferroptosis, Acute kidney injury-to-chronic kidney disease transition, Mechanistic target of rapamycin complex 1 (mTORC1), Glutathione peroxidase 4 (GPX4), Molybdenum Oxide Nanozyme, Fe^2+^-mTORC1-GPX4 Axis

## Abstract

AKI-to-CKD progression is shaped by sustained tubular stress, ferroptosis-related iron imbalance, and oxidative injury. To target this process, we developed an MHY1485-loaded molybdenum oxide (MoOx) nanozyme coated with a pH-responsive phenylboronic acid-polyethylene glycol-phenylboronic acid (PBA-PEG-PBA) polymer (MHY@MO@PPP). We tested whether this platform could weaken the ferroptosis-associated injury loop and delay AKI-to-CKD transition through regulation of the Fe^2+^-mechanistic target of rapamycin complex 1 (mTORC1)-glutathione peroxidase 4 (GPX4) axis. Material properties were examined by dynamic light scattering (DLS), transmission electron microscopy (TEM), X-ray photoelectron spectroscopy (XPS), and liquid chromatography-tandem mass spectrometry (LC-MS/MS). Biological activity was assessed in erastin-induced ferroptotic human renal proximal tubular epithelial (HK-2) cells and in a C57BL/6 mouse unilateral ischemia-reperfusion injury (UIRI) model. MHY@MO@PPP showed stimulus-responsive MHY1485 release under simulated lesion-like conditions, lowered reactive oxygen species (ROS), lipid peroxidation, and free Fe^2+^, restored mitochondrial function, and improved cell survival. Mechanistic assays showed increased p-mTOR and GPX4 expression with reduced autophagy-related stress, supporting attenuation of the ferroptosis-related feedback state. Proteomic analysis and functional intervention experiments further implicated the Fe^2+^-mTORC1-GPX4 axis in the protective response. In vivo, MHY@MO@PPP improved renal function and reduced renal tissue injury and fibrosis. These findings support a nanozyme-based strategy that combines catalytic redox regulation with pathway modulation to mitigate AKI-to-CKD progression.

**Graphical abstract:**

**Figure:**
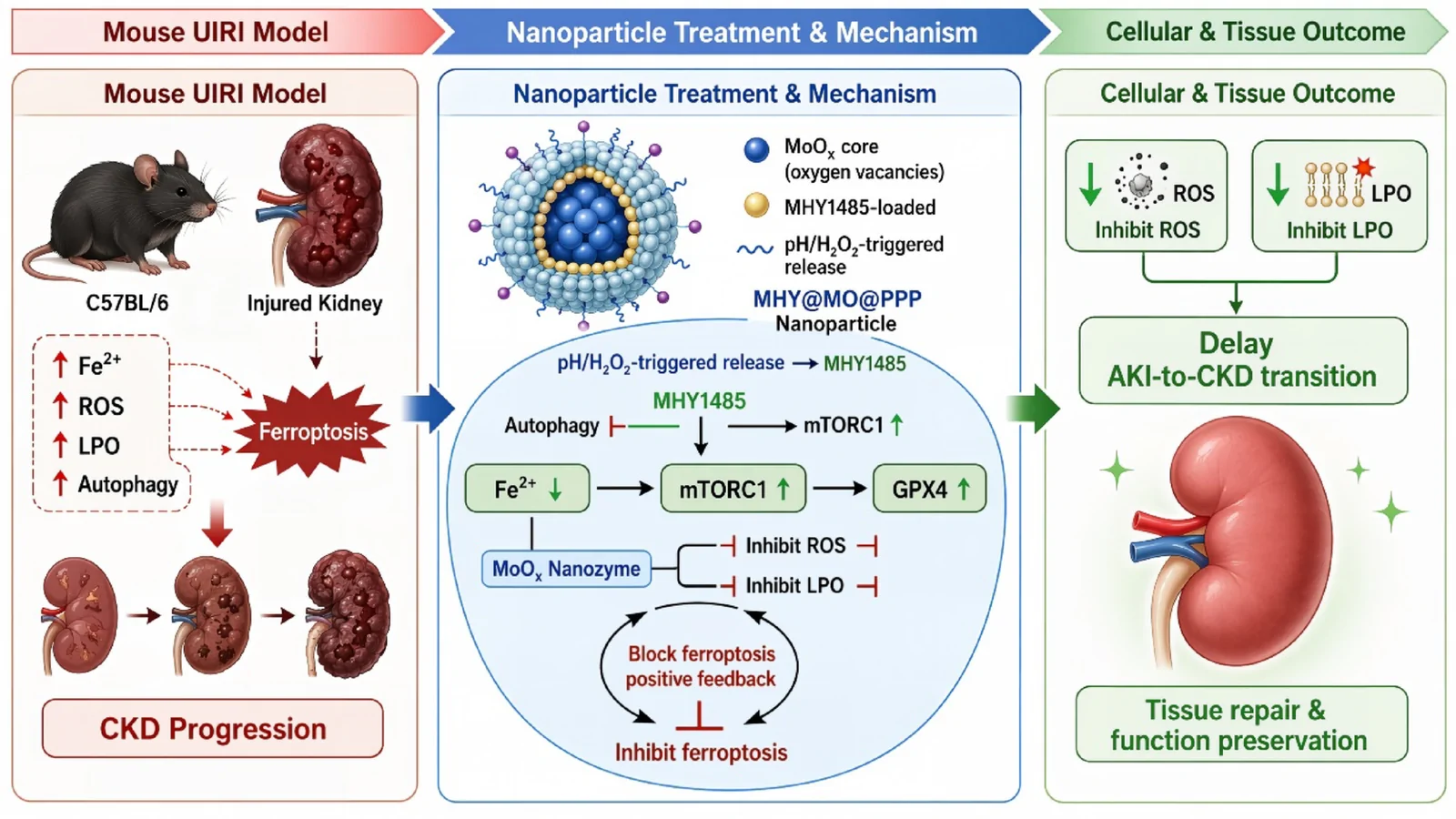

**Supplementary Information:**

The online version contains supplementary material available at https://doi.org/10.1186/s12951-026-04918-w.

## Introduction

Acute kidney injury (AKI) is a common and clinically serious renal disorder, particularly among hospitalized and critically ill patients, and is associated with high mortality and unfavorable long-term outcomes [1, 2]. AKI was once viewed mainly as a reversible event, but accumulating evidence shows that it increases the subsequent risk of chronic kidney disease (CKD) and end-stage renal disease, making it an important trigger of renal chronicity [1]. Current models describe a continuum from AKI to acute kidney disease and then to CKD, in which maladaptive repair after tubular epithelial injury is a central pathological feature [2, 3]. Severe or repeated injury can maintain inflammation, induce cell-cycle arrest, and promote profibrotic signaling in renal tubules, ultimately contributing to tubulointerstitial fibrosis and nephron loss [2]. In animal studies, the unilateral ischemia-reperfusion injury (UIRI) model develops progressive tubulointerstitial fibrosis and renal atrophy after the acute insult, making it useful for studying AKI-to-CKD mechanisms [4]. Persistent oxidative stress and metabolic dysregulation during the repair phase are increasingly recognized as drivers of renal chronicity [1]. Effective interventions that prevent AKI-to-CKD transition remain limited, underscoring the need for new mechanistic targets and therapeutic strategies.

Ferroptosis is an iron-dependent form of regulated cell death driven by lipid peroxidation and has emerged as an important contributor to AKI initiation and progression [5]. Hallmarks of this process include intracellular Fe^2+^ accumulation, increased reactive oxygen species (ROS), and lipid peroxidation, whereas glutathione peroxidase 4 (GPX4) functions as a key antioxidant defense molecule [5]. In renal ischemia-reperfusion injury, ferroptosis damages tubular epithelial cells and can amplify inflammatory and fibrotic responses, thereby facilitating AKI-to-CKD progression [6]. Loss of GPX4 expression or stability further increases ferroptosis susceptibility; for example, TRIM21-mediated GPX4 ubiquitination aggravates ischemic AKI [7]. Ferritinophagy can also raise intracellular Fe^2+^ by releasing stored iron, and the STING-NCOA4 pathway has been linked to this process in ischemic renal injury [8]. In addition, dysregulated autophagy may destabilize GPX4 and intensify ferroptotic signaling, forming a persistent injury-amplifying loop [9]. Thus, restoration of iron homeostasis and GPX4-mediated protection represents a rational approach for limiting AKI-associated damage.

The mechanistic target of rapamycin complex 1 (mTORC1) pathway is a metabolic signaling node that links cellular stress responses with antioxidant defense in the ferroptosis regulatory network [10]. mTORC1 activity promotes GPX4 protein translation and strengthens resistance to lipid peroxidation, whereas mTORC1 inhibition lowers GPX4 abundance and increases ferroptosis vulnerability [10]. Activation of mTOR signaling by the agonist MHY1485 has also been reported to alleviate renal ischemia-reperfusion injury and suppress ferroptosis [11]. However, efficient delivery and sustained local action of small molecules in injured renal tissue remain difficult, highlighting the need for delivery systems with improved lesion exposure [11]. Nanozymes, which combine enzyme-like catalytic activity with favorable biocompatibility, have attracted attention in oxidative stress-related diseases [12]. Molybdenum oxide nanozymes (MoOx) containing oxygen vacancies can scavenge ROS, regulate redox balance, and show renal-protective potential in AKI models [12].

On this basis, we engineered an MHY1485-loaded MoOx nanozyme coated with a pH-responsive PBA-PEG-PBA polymer (MHY@MO@PPP) to couple catalytic ROS regulation with mTORC1-GPX4 pathway modulation. The MoOx component was designed to relieve oxidative stress through ROS scavenging, while MHY1485 was incorporated to enhance mTORC1 signaling and support GPX4-dependent antioxidant protection. The pH-responsive polymer coating provided a physicochemical basis for preferential drug release under acidic and oxidative microenvironmental conditions, thereby improving local exposure in injured tissue. We then evaluated whether the Fe^2+^-mTORC1-GPX4 axis participates in ferroptosis-associated renal injury and whether MHY@MO@PPP can interrupt the injury loop by lowering Fe^2+^ burden, activating mTORC1 signaling, and restoring GPX4 expression. Using cellular ferroptosis assays and a UIRI-induced AKI-to-CKD model, we assessed the effects of MHY@MO@PPP on oxidative stress, iron metabolism, and fibrotic progression. This study provides experimental evidence for a ferroptosis-targeted nanotherapeutic approach in AKI-to-CKD transition.

## Materials and methods

### Synthesis of MoOx nanozymes

To synthesize the hydrogen molybdenum bronze nanozyme Hx(MoVx)(MoVI1-x)O3 (denoted as MoOx), molybdenyl acetylacetonate (54 mg; Aladdin, China) and hexadecylamine (0.30 g; Sigma-Aldrich, USA) were sequentially added to a mixed solvent containing oleic acid (3 mL; Sigma-Aldrich, USA) and 1-octadecene (12 mL; Sigma-Aldrich, USA). The system was ultrasonically dispersed (40 kHz) for 10 min, then rapidly heated to 100 °C in an oil bath and maintained for 8 min. Subsequently, the reaction mixture was transferred to a 25 mL Teflon-lined autoclave (Shanghai Yarong, China) and heated at 160 °C for 12 h. Upon completion, the mixture was cooled to room temperature, and the black product was collected via centrifugation (10,000×g, 10 min). The precipitate was washed 3–5 times with anhydrous ethanol (Sinopharm, China) to remove residual organic matter and unreacted precursors, and finally dispersed in cyclohexane (10 mL; Sigma-Aldrich, USA) to obtain a stable MoOx nanozyme stock solution.

### Construction of nanocomposites (MHY@MO@PPP)

To construct microenvironment-responsive nanocomposites, MoOx was utilized as the core carrier. MHY1485 (MilliporeSigma, USA, Cat#SML0810) was loaded onto the MoOx surface via electrostatic adsorption and hydrophobic interactions to form the MoOx@MHY intermediate. Subsequently, the particles were surface-coated with PBA-PEG-PBA (phenylboronic acid-polyethylene glycol-phenylboronic acid; Ruixi Biology, China, Cat#R-20906-2k) to generate MHY@MO@PPP. The resulting complex was centrifuged and washed three times to remove free drugs and polymers, and finally resuspended in phosphate-buffered saline (PBS; pH 7.4) at a concentration of 10 mg/mL for storage. For in vitro experiments, dosages were determined based on preliminary trials: the final concentration of free MHY1485 was set at 10 µM, the final concentration of bare MoOx was 200 µg/mL, and MHY@MO@PPP was prepared to contain an equivalent MHY1485 concentration of 10 µM.

### Dynamic light scattering (DLS)

To evaluate particle size distribution and surface electrical properties, MoOx and MHY@MO@PPP were characterized using a DLS/electrophoretic light scattering system (Zetasizer Nano ZS90, Malvern Panalytical, UK). Samples were diluted to 0.2 mg/mL with phosphate-buffered saline (PBS) or PBS containing 10% fetal bovine serum (FBS) and equilibrated at room temperature for 10 min before data acquisition. Hydrodynamic diameter, polydispersity index (PDI), and Zeta potential were measured under the same acquisition settings. To further assess short-term colloidal stability in a protein-containing medium, MHY@MO@PPP dispersed in PBS containing 10% FBS was monitored by DLS at 0, 6, 12, and 24 h. Each experiment was independently repeated three times, and data are presented as mean ± SD where applicable.

### Transmission electron microscopy (TEM)

To observe the micromorphology and dispersity of the particles, dispersions of MoOx or MHY@MO@PPP were diluted to 0.05 mg/mL, deposited onto carbon-coated copper grids (200 mesh), and allowed to stand for 1 min. After removing excess liquid and natural air drying, the samples were imaged using a TEM (JEM-2100, JEOL, Japan) at an accelerating voltage of 200 kV. Representative fields were recorded to describe morphology and estimate particle size.

### Fourier transform infrared spectroscopy (FT-IR)

To identify functional groups and polymer modifications, the materials were analyzed using an FT-IR spectrometer (Nicolet iS50, Thermo Fisher Scientific, USA). The samples were thoroughly ground with KBr at a 1:100 ratio and pressed into pellets. Spectra were acquired over a scanning range of 4000–400 cm^− 1^ with a resolution of 4 cm^− 1^ and 32 accumulated scans. MoOx, MHY@MO@PPP, and relevant control samples were analyzed simultaneously to compare variations in characteristic peaks.

### Ultraviolet-visible (UV-Vis) absorption spectroscopy

To obtain the UV-Vis absorption spectra of the samples, measurements were performed using a UV-1601 spectrophotometer (Shimadzu, Japan). The samples (molybdenum oxide, MHY1485, and MHY@MO@PPP) were diluted with their corresponding dispersion media (deionized water or PBS) to a measurable absorbance range and transferred into 1 cm path length quartz cuvettes (Hellma, Germany, 104-QS). Using the same dispersion medium as a blank control for baseline correction, continuous scanning was performed over 200–1200 nm with a 1 nm step size, and the absorption spectra were recorded and exported for subsequent analysis.

### Raman spectroscopy

To evaluate the vibration of Mo-O bonds and changes in electronic structure following recombination, detection was carried out using a Raman spectrometer (inVia Reflex, Renishaw, UK). The samples were dropped onto clean silicon wafers and allowed to dry naturally before testing. The excitation wavelength was set to 532 nm, with an integration time of 10 s. The spectra were accumulated three times and averaged to compare the shifts and intensity variations of Mo-O-related vibrational peaks.

### X-ray photoelectron spectroscopy (XPS)

To quantitatively analyze the Mo valence composition and infer the oxygen vacancy content, Mo 3 d high-resolution spectra were acquired using an XPS system (ESCALAB 250, Thermo Fisher Scientific, USA). Following film formation via drying, samples were analyzed using Al Kα radiation (1486.6 eV). The binding energy was calibrated referencing C 1 s at 284.8 eV. Subsequently, Mo^6+^/Mo^5+^ peaks were fitted to calculate relative area ratios, thereby characterizing the structural features of the tunable oxygen vacancies.

### Source and culture of HK-2 cells

Human renal proximal tubular epithelial cells (HK-2) were obtained from ATCC (CRL-2190; ATCC, USA). These cells were cultured in Dulbecco’s modified Eagle medium/Ham’s F-12 (DMEM/F-12) medium (Gibco, USA) supplemented with 10% fetal bovine serum (FBS; Gibco, USA) and 1% Penicillin-Streptomycin (Gibco, USA), and maintained in a constant temperature incubator at 37 °C with 5% CO_2_ (Thermo Fisher Scientific, USA). Cells were subcultured at a 1:3 ratio, with passages 5–15 used in experiments to mitigate the risk of phenotypic drift.

### Establishment and grouping of the ferroptosis model

To establish the renal tubule cell ferroptosis model, HK-2 cells were seeded into 6-well plates (2.0 × 10^5^ cells/well) or 96-well plates (1.0 × 10^4^ cells/well). Following a 12-h adhesion period, cells were treated with Erastin (1 µM; HY-15763, MedChemExpress, USA) for 24 h to generate the ferroptosis model group (Era), whereas the control group (CON) remained untreated. For in vitro intervention assays, four groups were established: Era, Era + MHY, Era+MoOx, and Era + MHY@MO@PPP. Specifically, the Era group received exclusive Erastin (1 µM) treatment. In the Era + MHY group, MHY1485 (10 µM; HY-B0795, MedChemExpress, USA) was administered post-Erastin induction; similarly, the Era+MoOx group was treated with molybdenum oxide (200 µg/mL), and the Era + MHY@MO@PPP group received MHY@MO@PPP (normalized to an equivalent concentration of 10 µM MHY1485). All groups were co-incubated for 24 h before subsequent analysis.

To verify the dependence of the material’s effects on iron and mTOR signaling, deferoxamine (DFO; 100 µM; Sigma-Aldrich, USA) or Rapamycin (100 nM; Selleck Chemicals, USA) was co-administered with Era + MHY@MO@PPP for 24 h. Subsequently, Western blot, JC-1 staining, ROS assessment, malondialdehyde (MDA)/glutathione (GSH) assays, FerroOrange staining, and flow cytometry were conducted to evaluate the molecular and phenotypic alterations following pathway inhibition.

### GPX4 silencing and transfection grouping (shRNA)

To verify the necessity of GPX4 in the protective effect of MHY@MO@PPP, a lentivirus-mediated shRNA knockdown strategy was employed. Two independent shRNA sequences targeting the human GPX4 gene (sh-GPX4#1 and sh-GPX4#2) and a negative control sequence (sh-NC) were synthesized by GeneChem Co., Ltd. (Shanghai, China) (Table S1) and cloned into the GV248 vector (hU6-MCS-CMV-EGFP-Puro). Transfection for packaging was performed using Lipofectamine 2000 (Thermo Fisher Scientific, 11668019, USA) according to the manufacturer’s instructions. Six hours post-transfection, the medium was replaced with antibiotic-free complete medium. Viral supernatants were collected at 48 h and 72 h, filtered through a 0.45 μm filter (Millipore, SLHP033RS, USA), and stored. The viral titer was estimated via EGFP positivity rates and converted to TU/mL, with the final titer maintained at approximately 1 × 10^8^ TU/mL. Aliquots were stored at −80 °C to avoid repeated freeze-thaw cycles.

HK-2 cells were purchased from ATCC (CRL-2190^™^) and cultured in DMEM/F12 medium (Gibco, 11320-033, USA) supplemented with 10% FBS (Gibco, 10099-141, USA) and 1% penicillin-streptomycin (Gibco, 15140-122, USA). Cells were seeded at a density of 2.0 × 10^5^ cells/well in 6-well plates and cultured overnight before infection. When cell confluence reached 50–60%, lentiviruses (sh-NC, sh-GPX4#1, or sh-GPX4#2) were added along with Polybrene (Sigma, TR-1003-G, USA) at a final concentration of 8 µg/mL to enhance infection efficiency, with the multiplicity of infection (MOI) set at 20. The medium was replaced with fresh complete medium 24 h post-infection. Selection was initiated 48 h post-infection by adding puromycin (InvivoGen, ant-pr-1, USA) at 2 µg/mL and maintained for 48–72 h until cells in the non-infected control wells died completely. sh-GPX4#2 exhibited higher knockdown efficiency and was therefore selected for subsequent experiments.

### Measurement of mitochondrial membrane potential

To assess changes in mitochondrial membrane potential, the JC-1 mitochondrial membrane potential assay kit (C2006, Beyotime Biotechnology, China) was employed. Following treatment, cells were incubated with JC-1 working solution (1×) at 37 °C for 20 min. After washing twice with the buffer supplied in the kit, red/green fluorescence images were acquired using a fluorescence microscope (ECLIPSE Ti2, Nikon, Japan), and the red-to-green ratio was calculated for quantitative analysis.

### Intracellular ROS detection

To examine intracellular ROS levels, the ROS assay kit DCFH-DA (S0033S, Beyotime Biotechnology, China) was utilized. Treated cells were incubated with 10 µM DCFH-DA working solution at 37 °C in the dark for 30 min. Following three washes with PBS, images were captured using an inverted fluorescence microscope. Synchronously, fluorescence intensity was read on a microplate reader (SpectraMax iD5, Molecular Devices, USA) at Ex/Em = 488/525 nm for quantification.

### Detection of lipid peroxidation and antioxidant indicators

To evaluate lipid peroxidation and the antioxidant system, MDA and GSH were measured. Cells were lysed with RIPA lysis buffer (Beyotime Biotechnology, China), and the supernatant was collected. MDA and GSH levels were then quantified using the MDA assay kit (Beyotime Biotechnology, China) and GSH assay kit (Beyotime Biotechnology, China) according to the manufacturer’s instructions, with absorbance readings taken on a microplate reader. The results were normalized to protein concentration determined by the BCA method (Thermo Fisher Scientific, USA).

### Measurement of intracellular free iron ions

To detect intracellular Fe^2+^ levels, the FerroOrange probe (Dojindo, Japan) was employed. Following the cell treatment, a 1 µM FerroOrange working solution was added, and the cells were incubated at 37 °C in the dark for 30 min. After washing with PBS, images were acquired by fluorescence microscopy, and fluorescence intensity was quantified under consistent exposure conditions to determine changes in intracellular labile iron load.

### Cell viability assay

To assess cell viability, the Cell Counting Kit-8 (CCK-8) kit (Dojindo, Japan) was utilized. A 10 µL volume of CCK-8 working solution was added to each well of a 96-well plate, and the plate was incubated at 37 °C for 2 h. The absorbance was subsequently measured at 450 nm using a microplate reader. Six technical replicates were established for each group, and the results were analyzed to compare cellular metabolic activity among the treatment groups.

### Detection of cell death by flow cytometry

To quantitatively assess cell death, an Annexin V-FITC/PI apoptosis detection kit (BD Biosciences, USA) was used. Following treatment, cells were harvested and washed with PBS, then resuspended in binding buffer at a concentration of 1 × 10^6^ cells/mL. Subsequently, 5 µL of Annexin V-FITC and 5 µL of PI were added, and the mixture was incubated at room temperature in the dark for 15 min. Data acquisition was performed using a flow cytometer (BD FACSCanto II, BD Biosciences, USA) to calculate the proportion of death-related cells.

### Cellular uptake and mitochondria colocalization imaging

To evaluate the cellular uptake and mitochondria localization of the complex, MHY@MO@PPP was labeled with a DiI membrane dye (Invitrogen, USA). After removing the free dye, the labeled complex was incubated with cells for 4 h. Subsequently, mitochondria were stained with MitoTracker Green (Invitrogen, USA) at 37 °C for 30 min. The cells were then fixed with 4% paraformaldehyde (60536ES60, Yeasen Biotechnology, China) for 15 min and counterstained with DAPI. Multi-channel images were acquired using a laser scanning confocal microscope (LSM 880, Carl Zeiss AG, Germany), and the colocalization coefficient was calculated under identical threshold conditions for quantification.

### Western blot

Cells were lysed using RIPA lysis buffer (P0013B, Beyotime Biotechnology, China) supplemented with protease and phosphatase inhibitors (04693132001/04906837001, Roche, Switzerland). Protein concentrations were determined using the BCA assay (23225, Thermo Fisher Scientific, USA). For each sample, 30 µg of protein was loaded, separated by SDS-PAGE, and transferred onto PVDF membranes (IPVH00010, MilliporeSigma, USA). The membranes were blocked with 5% non-fat milk at room temperature for 1 h, incubated with primary antibodies overnight at 4 °C, and then incubated with HRP-labeled secondary antibodies for 1 h at room temperature. Chemiluminescent signals were visualized using ECL substrates (32106, Thermo Fisher Scientific, USA) and acquired via an imaging system (ChemiDoc MP, Bio-Rad, USA). Gray value quantification was conducted using ImageJ, and all target proteins were normalized to the internal control GAPDH.

Primary antibodies used were: GPX4 (Cell Signaling Technology, USA, Cat#52455, 1:1000), ACSL4 (Cell Signaling Technology, USA, Cat#21249, 1:1000), FTH1 (Abcam, UK, Cat#ab75973, 1:2000), p-mTOR (Ser2448) (Cell Signaling Technology, USA, Cat#5536, 1:1000), mTOR (Cell Signaling Technology, USA, Cat#2983, 1:1000), LC3B (Cell Signaling Technology, USA, Cat#2775, 1:1000), SOD2 (Cell Signaling Technology, USA, Cat#13141, 1:1000), and GAPDH (Cell Signaling Technology, USA, Cat#5174, 1:5000).

Secondary antibodies used were: HRP goat anti-rabbit IgG (Cell Signaling Technology, USA, Cat#7074, 1:5000) and HRP goat anti-mouse IgG (Cell Signaling Technology, USA, Cat#7076, 1:5000).

### RT-qPCR

Total RNA was extracted using the TRIzol reagent (Invitrogen, USA), and its purity and concentration were determined via a Nanodrop 2000 (Thermo Fisher Scientific, USA). Subsequently, 1 µg of RNA was reverse-transcribed using the PrimeScript RT reagent Kit (Takara, Japan). qPCR was performed on a real-time fluorescence quantitative PCR system (QuantStudio 5, Thermo Fisher Scientific, USA) utilizing the SYBR Green system (TB Green Premix Ex Taq, Takara, Japan). Target genes included KIM1, NGAL, CYSC, ACTA2, Fn1, and VIMENTIN (Table S2). Relative expression levels were calculated using the 2^−ΔΔCt^ method, with GAPDH serving as the internal reference.

### Proteomics sample preparation and peptide digestion

To obtain proteomics data for HK-2 cells in the Era and Era + MHY@MO@PPP groups, three biological replicates were collected for each group. Cells were washed twice with pre-cooled PBS (Gibco, USA, Cat#10010-023), lysed in RIPA lysis buffer (Beyotime, China, Cat#P0013B) supplemented with protease/phosphatase inhibitors (Roche, Switzerland, Cat#04693132001/04906837001) for 30 min, and centrifuged at 12,000 × g for 15 min at 4 °C to harvest the supernatant. Protein concentration was quantified using the BCA method (Thermo Fisher Scientific, USA, Cat#23225), and 100 µg of total protein per sample was digested. Proteins were initially reduced with DTT (10 mM, Sigma-Aldrich, USA, Cat#D0632) at 56 °C for 30 min and subsequently alkylated with IAA (20 mM, Sigma-Aldrich, USA, Cat#I1149) in the dark at room temperature for 30 min. Trypsin (Promega, USA, Cat#V5111) was then added at a ratio of 1:50 (enzyme: protein, w/w) for overnight digestion, followed by a second addition of trypsin (1:100, w/w) the next day for an additional 2 h of digestion. Peptides were desalted using a C18 solid-phase extraction column (Waters Sep-Pak C18, USA), dried by vacuum centrifugation, and reconstituted in 0.1% formic acid (FA, Sigma-Aldrich, USA, Cat#56302) to a concentration of 0.5 µg/µL.

### Liquid chromatography-tandem mass spectrometry (LC-MS/MS) data acquisition

Peptide separation was performed using an EASY-nLC 1200 nanoflow liquid chromatography system (Thermo Fisher Scientific, USA) coupled to a high-resolution Q Exactive HF-X mass spectrometer (Thermo Fisher Scientific, USA) for data-dependent acquisition (DDA). A self-packed reversed-phase C18 column (inner diameter 75 μm, length 25 cm, particle size 1.9 μm, Dr. Maisch, Germany) was employed as the chromatographic column. Mobile phase A consisted of 0.1% FA in water, while mobile phase B was 80% acetonitrile (ACN, Merck, Germany) containing 0.1% FA. A 120-min gradient elution was established: phase B increased from 5% to 30% (0–90 min), rose to 45% (90–110 min), and then increased to 80% (110–115 min), where it was maintained for 5 min for washing, with a flow rate of 300 nL/min. Mass spectrometry parameters were configured with an MS1 resolution of 60,000 (at m/z 200), a scan range of m/z 350–1600, an AGC target of 3e6, and a maximum injection time of 50 ms. For MS2, the resolution was set to 15,000 with Top20 selection, normalized collision energy (NCE) of 28, and dynamic exclusion of 30 s. All samples were analyzed in a randomized order to mitigate batch effects.

### Protein identification and quantification (Database Search)

Raw mass spectrometry data were processed using MaxQuant software (MaxQuant) for database searching and label-free quantification (LFQ). The search was conducted against the UniProt human reference protein database (UniProt, https://www.uniprot.org/, reviewed + isoforms), supplemented with a common contaminants database. Enzyme specificity was set to Trypsin/P with a maximum of 2 missed cleavages. Fixed modifications included carbamidomethylation of cysteine, while variable modifications comprised methionine oxidation and N-terminal acetylation. Default high-resolution settings were applied to precursor and fragment mass tolerances, and the false discovery rate (FDR) for both peptide and protein identification was set to 1%. LFQ quantification was enabled with “Match between runs” (matching window 0.7 min, alignment window 20 min). The “proteinGroups.txt” file was generated as the input for subsequent statistical analysis, with entries marked as “Reverse,” “Only identified by site,” and “Potential contaminant” excluded.

### Quality control and normalization of the quantitative matrix

The LFQ intensity data from the proteinGroups file were imported into R (R Foundation) for processing. Log_2_ transformation was performed on the LFQ intensity of each sample, and protein entries containing quantitative values in at least two-thirds of the replicates were retained. Missing values were imputed using a left-shifted normal distribution approach to simulate low-abundance protein signals, with the imputation width set to 0.3 and the down-shift set to 1.8 (relative to log_2_ intensity), ensuring that the imputed values fell below the main peak of the sample’s true distribution. The inter-sample distributions were examined using box plots and density plots, and global normalization was achieved via median alignment to ensure comparable overall intensities across samples.

### Analysis of differentially expressed proteins

Screening for differentially expressed proteins was conducted in R using a statistical framework based on linear models for inter-group comparisons (Era vs. Era + MHY@MO@PPP). A group design matrix was generated from the log_2_ intensity matrix. Following model fitting, the log_2_ fold change (FC) and p-values for each protein were extracted, and the Benjamini-Hochberg method was employed for multiple testing correction to obtain the FDR. Proteins with FDR < 0.05 were defined as statistically significant differential proteins, and the log_2_FC direction and magnitude were used to interpret biological relevance. To avoid overemphasizing very small changes, downstream interpretation focused on pathway-level enrichment and proteins showing consistent directional regulation rather than isolated minimal fold-change differences.

### Clustering heatmap of differentially expressed proteins

To visualize sample grouping and protein expression patterns, the log_2_ intensity matrix of differentially expressed proteins was row-normalized using Z-scores and clustered hierarchically. This analysis utilized Euclidean distance and the complete linkage method. Heatmaps were generated using the R graphics system, employing a blue-white-red color scale to represent expression levels ranging from low to high. Sample annotation bars designated the Era and Era + MHY@MO@PPP groups (*n* = 3 per group), visually confirming intra-group consistency and inter-group separability.

### Least absolute shrinkage and selection operator (LASSO) feature selection and cross-validation

To identify the most discriminative feature proteins from the pool of differentially expressed proteins, LASSO regression was employed for variable shrinkage and selection. Specifically, the log_2_ intensity matrix of differentially expressed proteins was used as the feature input, and the experimental groups (Era and Era + MHY@MO@PPP) served as binary classification labels. All features were centralized and standardized before analysis. A 10-fold cross-validation approach was implemented to determine the optimal penalty parameter (λ) and to identify the set of proteins with non-zero coefficients. Furthermore, a cross-validation curve was plotted to illustrate the relationship between λ and the binomial deviance error, explicitly marking the feature subsets corresponding to λ_min_ and λ_1se_ for subsequent model integration.

### Random forest importance ranking

The random forest algorithm was subsequently utilized to evaluate the relative contribution of protein features to group discrimination. Using the same input dataset as the LASSO analysis, a classification random forest model was constructed with 1,000 trees to ensure stable importance estimates, and the ‘mtry’ parameter was set to sqrt(p). The MeanDecreaseGini or MeanDecreaseAccuracy was calculated as the metric of importance, and the resulting values were visualized in a descending bar chart to highlight the top-ranked candidate key proteins.

### Recursive feature elimination based on support vector machine-recursive feature elimination (SVM-RFE)

To achieve optimal discrimination with a minimal feature set, SVM-RFE was used for screening. The differentially expressed protein intensity matrix served as the input features, and a linear kernel SVM was used to generate stable weights. Variables were sequentially eliminated from lowest to highest weight, and classification error was calculated at each step via cross-validation. A “Variables-Error” curve was plotted, and the number of features corresponding to the lowest error was selected as the optimal feature size.

### Intersection of multiple algorithms and identification of candidate proteins

Candidate proteins identified by LASSO, random forests, and SVM-RFE were intersected to reduce single-algorithm bias, and the resulting intersection was visualized using a Venn diagram. Box plots were subsequently generated for the key proteins within the intersection to display inter-group distributions, using log_2_ intensity as the ordinate and group as the abscissa, with significance levels for comparisons between the two groups indicated.

### Enrichment analysis of kyoto encyclopedia of genes and genomes (KEGG) pathways

To analyze the functional pathway reconstruction directions corresponding to differentially expressed proteins, KEGG enrichment analysis was performed separately for upregulated and downregulated proteins. The Xiantao Academic Database (https://www.xiantaozi.com/) was utilized for this analysis. The enrichment results were visualized using two methods: bar charts, which indicated significance via -log_10_(FDR) and listed the top pathways; and bubble charts, which plotted GeneRatio on the abscissa and pathways on the ordinate. In the bubble charts, bubble size represented counts, while color indicated the corrected significance level, simultaneously presenting both enrichment intensity and coverage scale.

### Ethical approval

All animal experiments were approved by the Institutional Animal Care and Use Committee (IACUC) of Jilin University (No. SY202509003).

### UIRI-induced AKI-to-CKD transition model

To establish an animal model of the AKI-to-CKD transition, male C57BL/6 mice (8 weeks old, 20–22 g; Beijing Vital River Laboratory Animal Technology Co., Ltd., China) were employed. Anesthesia was maintained with isoflurane (RWD Life Science Co., Ltd., China) via inhalation. A left UIRI model was established by isolating the renal pedicle, clamping it for 30 min, and then releasing the clamp to allow reperfusion. Following suturing, the mice were kept warm during recovery. In the Sham group, the renal pedicle was exposed but not clamped. Postoperatively, analgesia was administered in accordance with animal ethical standards, and body weight and mental status were monitored daily.

### Tail vein injection and experimental grouping

For in vivo efficacy and safety evaluation, mice were randomized into seven groups (*n* = 10 per group): Sham, UIRI, UIRI + MHY, MHY@MO, MHY@MO@PPP, UIRI + MHY@MO, and UIRI + MHY@MO@PPP. No formal a priori power analysis was performed; therefore, the sample size justification is descriptive and based on prior UIRI and nanomaterial intervention studies using comparable histological and renal-function endpoints. Animals were assigned to groups using a random-number procedure, and histological scoring and image quantification were performed by investigators blinded to group allocation. All treatments were administered via tail vein injection using a 29G insulin syringe (BD, USA), with the injection volume calibrated to 5 mL/kg body weight (approximately 100 µL for a 20 g mouse) to ensure consistent dose exposure across individuals of varying weights.

To ensure dose comparability for the main treatment groups, MHY-related groups received equivalent MHY1485 doses, and MoOx-containing groups were normalized according to MoOx mass or Mo content where applicable. Specifically: (1) the UIRI + MHY group received free MHY1485 at 1 mg/kg/day; (2) the UIRI + MHY@MO group received the MHY1485-loaded MoOx intermediate without the PBA-PEG-PBA coating, with MHY1485 normalized to 1 mg/kg/day and the corresponding MoOx amount matched to the composite formulation; and (3) the UIRI + MHY@MO@PPP group received composite nanoparticles at a dosage calculated to deliver an equivalent MHY1485 dose. All formulations were prepared in sterile PBS, with the final volume adjusted to 5 mL/kg. A bare MoOx-only in vivo efficacy group was not included, which is acknowledged as a limitation for component-specific attribution.

Administration commenced 24 h after the completion of ischemia-reperfusion (representing immediate/delayed intervention post-reperfusion), followed by once-daily administration for 21 consecutive days. Body weight was recorded throughout the treatment period to adjust injection volumes daily, ensuring a constant actual dosage (mg/kg). Upon sacrifice, heart, liver, spleen, and lung tissues were immediately harvested under sterile conditions and stained with hematoxylin and eosin (H&E) to assess in vivo safety.

### Near-infrared (NIR) in vivo imaging

To evaluate the short-term systemic distribution and renal-region fluorescence enrichment of the nanoparticles in vivo, NIR imaging was performed using a small animal imaging system (IVIS Spectrum, PerkinElmer, USA). Following tail vein injection of the nanomaterials, mice underwent dynamic in vivo imaging at Prescan, 1, 5, 10, and 30 min post-administration. Before imaging, mice were anesthetized with 2.0% isoflurane and maintained at 1.5% isoflurane (RWD Life Science Co., Ltd., China). The animals were placed in a 37 degrees C thermostatic imaging chamber to maintain body temperature, and images were acquired in both supine and prone positions to cover major organ regions. short-term distribution After fluorescently labeled MHY@MO or MHY@MO@PPP Prescan, 1, 5, 10, and 30 min, and images the renal region and body regions.

The imaging parameters were standardized as follows: excitation filter Ex = 745 nm, emission filter Em = 800 nm; exposure time = 10 s; F/Stop = 2; Bin = Medium; field of view (FOV) = D; and excitation intensity set to Auto. The same parameters were used for all animals and time points. Quantitative analysis was conducted using Living Image software (PerkinElmer, USA). Regions of interest (ROIs) covering the whole body and bilateral kidney areas were delineated in the same posture, and average radiance (photons/s/cm2/sr) was exported after background correction using Prescan values or corresponding control-group signals as the baseline. The same used for animals and performed each Average exported, were subtracted Prescan control-signals baseline.

### Ex vivo IVIS imaging of major organs at 24 h post-injection

For 24 h ex vivo biodistribution analysis, fluorescently labeled MHY@MO or MHY@MO@PPP was administered by tail-vein injection. At 24 h post-injection, mice were anesthetized and perfused with PBS to reduce circulating fluorescence background. Major organs, including the heart, liver, spleen, lung, contralateral kidney, and injured kidney, were harvested and immediately imaged by IVIS under identical acquisition parameters. Regions of interest (ROIs) were drawn around each organ using Living Image software, and fluorescence intensity was quantified according to the system output. To evaluate renal tissue-level localization, frozen kidney sections collected at 24 h post-injection were examined by laser scanning confocal microscopy (LSCM). Fluorescence signals were acquired with consistent laser power and gain settings, and renal-section fluorescence intensity was quantified using the same threshold strategy across groups.

### Frozen kidney-section LSCM imaging

To further validate renal tissue localization of the nanomaterials, kidneys were collected at 24 h after intravenous injection of fluorescently labeled MHY@MO or MHY@MO@PPP. Fresh kidney tissues were embedded in OCT compound, frozen, and cut into 6–10 μm sections. Sections were fixed with 4% paraformaldehyde, washed with PBS, counterstained with DAPI, and imaged using a laser scanning confocal microscope (LSM 880, Carl Zeiss AG, Germany) under identical laser power, gain, and exposure settings. Fluorescence intensity or fluorescence-positive area was quantified using ImageJ under the same threshold settings across groups.

### Hemolysis assay

To assess the hemocompatibility of the nanomaterials, a hemolysis assay was conducted. Whole blood was collected into EDTA-K2 anticoagulant tubes (BD Biosciences, USA, Cat#367841) and diluted 1:10 with physiological saline (0.9% NaCl; Baxter, USA, Cat#2F7123). The mixture was centrifuged at 1500 × g for 5 min, and the supernatant was discarded. The erythrocyte pellet was washed three times with sterile PBS (pH 7.4; Gibco, USA, Cat#10010-023) to prepare a final 2% (v/v) erythrocyte suspension. PBS, MHY@MO, and MHY@MO@PPP were mixed with the 2% erythrocyte suspension at a 1:1 volume ratio, resulting in a final material concentration of 200 µg/mL. After incubation at 37 °C with constant oscillation for 2 h (Thermo Fisher Scientific, USA, MaxQ 4450), the samples were centrifuged, and the absorbance of the supernatant was measured at 540 nm. The hemolysis ratio was calculated relative to the negative control (PBS) and positive control (distilled water). This procedure was repeated three times independently.

### Serum aspartate aminotransferase (ast)/alanine aminotransferase (ALT) detection

To evaluate in vivo hepatotoxicity-related safety, serum AST and ALT levels were measured. Twenty-four hours after the final administration, mice in the Sham, MHY@MO, and MHY@MO@PPP groups were fasted for 6 h with ad libitum access to water. Subsequently, anesthesia was induced via isoflurane inhalation (RWD Life Science Co., Ltd., China, R510), and approximately 0.6 mL of blood was collected from the retro-orbital venous plexus into clot activator tubes (BD Biosciences, USA, Cat#367820). After standing at room temperature for 30 min to allow coagulation, the samples were centrifuged at 3000 × g for 10 min at 4 °C to isolate the serum.

The levels of AST and ALT were determined using commercial assay kits (Nanjing Jiancheng Bioengineering Institute, China; Cat# C010-2-1 for AST and Cat# C009-2-1 for ALT) strictly following the manufacturer’s protocols. The reaction systems were maintained at 37 °C (Eppendorf, Germany, ThermoMixer C). After color development, absorbance was measured at 510 nm using a microplate reader (BioTek, USA, Synergy H1), and the activities of AST and ALT (U/L) were calculated from the standard curves provided with the kits. All serum samples were analyzed in triplicate technical replicates, with 10 animals per group.

### Assessment of renal function markers

To evaluate renal function recovery, serum was separated from orbital blood collected from mice after the final administration. The levels of blood urea nitrogen (BUN) and creatinine (CRE) were detected using commercial kits (Nanjing Jiancheng Bioengineering Institute, China), and the results were obtained using an automatic biochemical analyzer (AU480, Beckman Coulter, USA).

### H&E, masson, and periodic acid-schiff (PAS) staining

To assess the extent of renal tubule injury and interstitial fibrosis, paraffin-embedded kidney Sect. (4 μm) were subjected to H&E, Masson’s trichrome, and PAS staining. H&E staining was performed using a H&E Staining Kit (Beyotime Biotechnology, China, C0105S), while Masson’s trichrome staining employed a Masson Tricolor Staining Kit (Servicebio, China, G1006). Additionally, PAS staining was conducted utilizing a PAS Stain Kit (Servicebio, China, G1008). Following the staining procedures, representative field images were acquired using a bright-field microscope (BX53, Olympus, Japan) under uniform magnification and identical exposure parameters. Specifically, H&E staining facilitated scoring of renal tubular injury, whereas Masson and PAS staining were used to quantify fibrosis/basement membrane-associated areas. The percentage of positive area (%Area) was calculated in ImageJ using a standardized threshold and a consistent ROI strategy.

### Immunohistochemistry (IHC)

To evaluate the expression and localization of pathway-related proteins, fibrosis markers, and inflammatory infiltration markers in kidney tissues, paraffin Sect. (4 μm) were deparaffinized, rehydrated, and subjected to antigen retrieval in citrate buffer (pH 6.0) using a microwave for 10 min. Endogenous peroxidase was blocked with 3% H_2_O_2_ at room temperature for 10 min. Subsequently, sections were blocked with 5% BSA (36101ES25, Yeasen Biotechnology, China) for 30 min at room temperature and incubated with primary antibodies overnight at 4 °C. The following day, sections were incubated with HRP-labeled secondary antibodies for 30 min at room temperature, followed by DAB color development (K3468, Dako, Denmark) and hematoxylin counterstaining. Images were captured using a bright-field microscope (BX53, Olympus, Japan), and the positive area (%Area) was quantified using ImageJ under a uniform threshold. Primary antibody information: p-mTOR (Ser2448) (CST, USA, Cat#5536), GPX4 (CST, USA, Cat#52455), FTH1 (Abcam, UK, Cat#ab75973), α-SMA (Abcam, UK, Cat#ab5694), Fn1 (Abcam, UK, Cat#ab2413), and F4/80 (Bio-Rad, USA, Cat#MCA497GA). Secondary antibody information: HRP goat anti-rabbit IgG (CST, USA, Cat#7074) and HRP goat anti-mouse IgG (CST, USA, Cat#7076).

### Immunofluorescence (IF)

Following deparaffinization, rehydration, and antigen retrieval of tissue sections, blocking was performed using 5% BSA (36101ES25, Yeasen Biotechnology, China) for 30 min at room temperature, followed by incubation with primary antibodies overnight at 4 °C. The following day, sections were incubated with fluorescently labeled secondary antibodies for 1 h at room temperature in the dark, counterstained with DAPI (D1306, Thermo Fisher Scientific, USA), and mounted with an antifade mounting medium (P0126, Beyotime Biotechnology, China). Multi-channel images were acquired using a laser scanning confocal microscope (LSM 880, Carl Zeiss AG, Germany). Consistent laser power, gain, and exposure parameters were applied across all groups, and fluorescence intensity/positive area was quantified under a uniform threshold. Primary antibody information: Fn1 (Abcam, UK, Cat#ab2413). Secondary antibody information: Alexa Fluor 488 goat anti-rabbit IgG (Invitrogen, USA, Cat#A-11008) and Alexa Fluor 594 goat anti-mouse IgG (Invitrogen, USA, Cat#A-11005).

### Statistical software and data analysis methods

Data analysis was performed using R (version 4.2.1) and compiled in RStudio (version 2022.12.0–353). File processing was conducted using Perl (version 5.30.0), and figures were generated using GraphPad Prism software (version 8.0). Quantitative data were presented as mean ± standard deviation. Comparisons between two groups were assessed using independent sample t-tests. Comparisons among multiple groups were conducted using one-way ANOVA followed by Tukey’s post hoc test unless otherwise specified. For data comparisons across different time points or two experimental factors, two-way ANOVA followed by Bonferroni post hoc tests was employed. A significance level of *p <* 0.05 was considered statistically significant.

## Results

### Successful construction and physicochemical characterization of MoOx nanozymes

MoOx nanozymes with adjustable oxygen-vacancy features were first prepared as the catalytic core of the platform (Figure S1A). This design was intended to accelerate hydrogen peroxide decomposition, neutralize •OH/O_2_•^−^ radicals, and support GPX4-associated antioxidant defense in the ferroptosis-related microenvironment. In combination with MHY1485, the nanozyme was used to modulate the Fe^2+^-mTORC1-GPX4 profile rather than relying on a single antioxidant mechanism.

To verify the successful construction of MoOx nanozymes, we first systematically analyzed their morphology and microstructure. TEM results indicated that the constructed nanozymes appeared as uniformly dispersed nanodot structures with diameters concentrated around 300 nm (Figure S1B-C). Furthermore, the structure of the MoOx nanounits was characterized via FT-IR (Figure S1D).

Oxygen vacancy is a critical structural attribute that determines the catalytic performance and ROS-regulation capability of MoOx. Therefore, we quantitatively analyzed the chemical valence composition of Mo using XPS. The Mo 3 d high-resolution spectrum clearly distinguished two characteristic peaks corresponding to Mo6 + and Mo5+, suggesting that the nanozyme is rich in tunable oxygen vacancy structures (Figure S1E). This valence distribution is highly consistent with the reported high ROS-scavenging capacity of oxygen-vacancy MoOx ([13]), establishing a structural foundation for subsequent ferroptosis regulation. Additionally, UV-vis-NIR spectroscopy revealed an extremely broad absorption band in the NIR-II region (Figure S1F).

Continuous monitoring at 4 degrees C for 30 days showed no obvious macroscopic aggregation or precipitation of the nanozyme, although the solution color changed during storage (Figure S1G). This color shift is consistent with gradual alteration of the oxidation state or surface coordination environment of molybdenum oxide; therefore, the stability conclusion is limited to colloidal dispersibility under the tested storage condition. To mimic a protein-containing biological medium, MHY@MO@PPP was incubated in PBS containing 10% FBS. Compared with PBS alone, PBS +10% FBS produced a mild increase in hydrodynamic diameter and a slight decrease in the absolute Zeta potential, consistent with serum protein adsorption or protein-corona formation and partial shielding of surface charge (Figure S1H-I). During 0–24 h monitoring in PBS +10% FBS, the particle-size distribution remained mainly concentrated without an obvious large-aggregate peak, supporting short-term colloidal stability in the protein-containing medium (Figure S1J).

Successful Construction of MHY@MO@PPP with Physicochemical Stability and Stimulus-Responsive Release Characteristics.

**Fig. 1:**
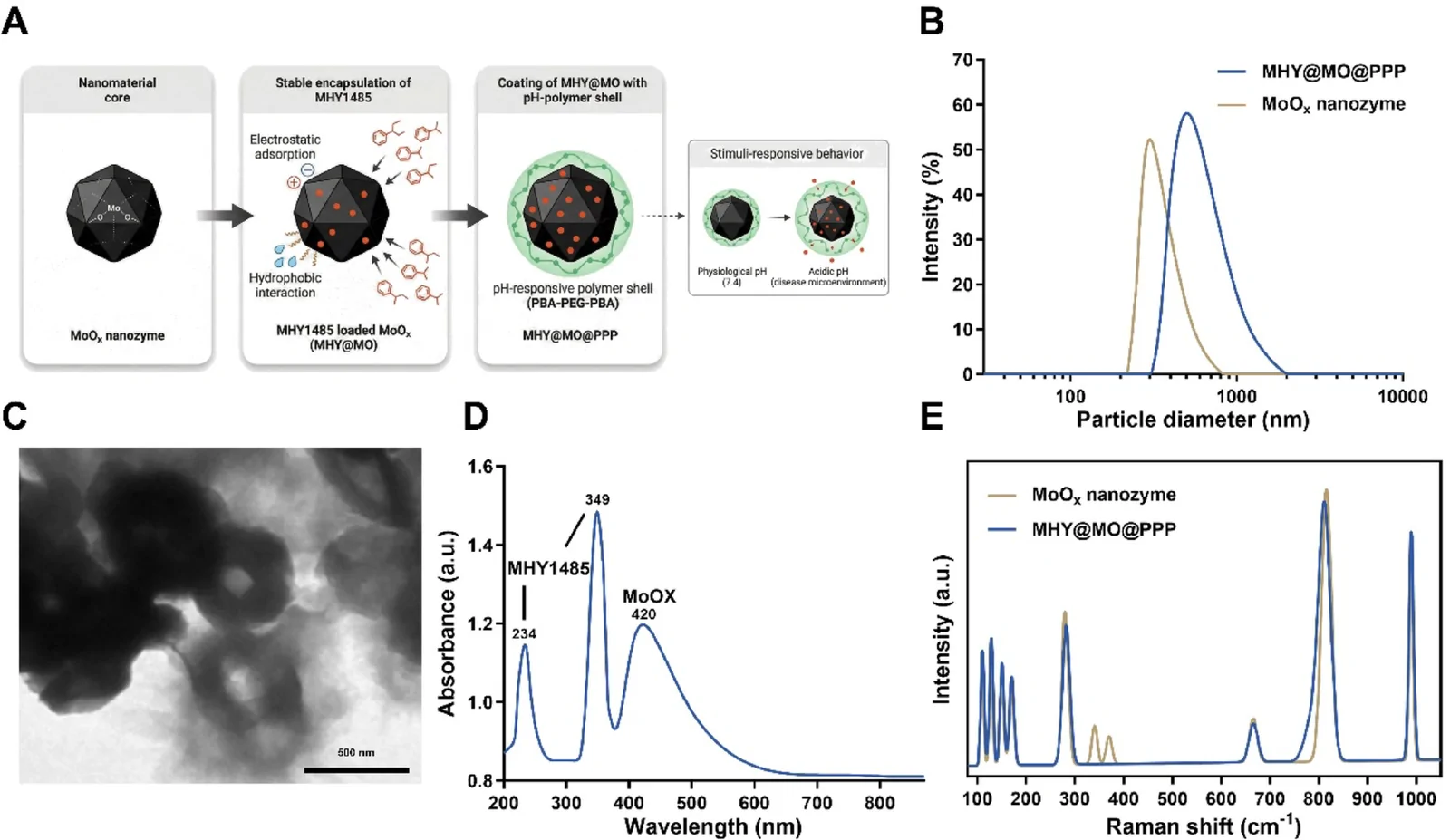
Construction and physicochemical characterization of MHY@MO@PPP nanozyme complex. (**A**) Schematic illustration showing the construction strategy of the MHY@MO@PPP nanozyme complex, the loading method of MHY1485, and the pH/H_2_O_2_ stimuli-responsive release design; (**B**) Hydrodynamic size distribution of MoOx and the MHY@MO@PPP nanozyme complex determined by DLS; (**C**) Morphology of the MHY@MO@PPP nanozyme complex observed by TEM, bar: 500 nm; (**D**) Optical absorption properties of the MHY@MO@PPP nanozyme complex detected by UV-Vis absorption spectroscopy to confirm the loading of MHY1485; (**E**) Mo-O related vibration modes in the MHY@MO@PPP nanozyme complex detected by Raman spectroscopy to analyze the electronic structure changes after MHY1485 loading

Previous results successfully established and systematically characterized the molybdenum oxide nanozyme platform with oxygen vacancy structures. To achieve microenvironment-responsive intervention, this study further incorporated MHY1485 to construct the MHY@MO@PPP nanozyme complex. The construction of MHY@MO@PPP used molybdenum oxide as the core, enabling stable loading of MHY1485 via electrostatic adsorption and hydrophobic interactions. Simultaneously, a pH-responsive polymer layer (PBA-PEG-PBA) was introduced onto the molybdenum oxide surface to endow the platform with ferroptosis-associated responsive release capability under acidic and oxidative microenvironment-like conditions (Fig. 1A). 

Particle-size and morphology analyses were then used to examine whether MHY1485 loading and polymer coating altered the basic architecture of the nanozyme. DLS showed an overall increase in hydrodynamic diameter after formation of MHY@MO@PPP, consistent with drug loading and surface modification (Fig. 1B). TEM imaging showed dispersed particles with a regular morphology and no obvious collapse or aggregation, indicating that the composite construction preserved the structural integrity of the MoOx core (Fig. 1 C).

Spectroscopic characterization further supported the incorporation of MHY1485 into the MoOx-based system. In the UV-vis spectra, MHY@MO@PPP displayed additional absorption features within the characteristic range of MHY1485 (Fig. 1D). Raman analysis also detected changes in Mo-O-related vibration signals after loading, suggesting interaction with the electronic environment of MoOx while retaining the host nanozyme framework (Fig. 1E).

After confirming MHY1485 loading, we examined the encapsulation efficiency and release behavior of the MHY@MO@PPP nanozyme complex. LC-MS/MS detected stable characteristic ion signals of MHY1485 in MHY@MO@PPP, confirming successful drug encapsulation (Figure S2A). In vitro release assays showed that MHY1485 release changed significantly under simulated acidic or high-H2O2 conditions compared with neutral conditions, indicating that PBA-PEG-PBA modification conferred stimulus-responsive release behavior (Figure S2B-C). Cellular uptake was then evaluated by co-incubating HK-2 cells with DiI-labeled MoOx-based formulations or the free-MHY1485 comparator for 24 h. Laser scanning microscopy showed the strongest intracellular red fluorescence and cytoplasmic distribution in the MHY@MO@PPP group, indicating higher cellular uptake efficiency of the composite formulation under pathological stress. The MoOx group also showed intracellular fluorescence, but the overall signal was weaker than that in the MHY@MO@PPP group, suggesting that the polymer-coated composite configuration may improve particle-cell interactions and endocytosis. Free MHY1485 lacks particle-based fluorescence tracing; therefore, its signal should be interpreted as a comparator rather than direct nanoparticle uptake; nanocarrier loading may increase effective intracellular exposure to MHY1485 during ferroptotic stress (Figure S2D).

Together, these characterization results show that MHY@MO@PPP retains the structural features required for subsequent biological testing and has the physicochemical basis for microenvironment-responsive MHY1485 exposure.

### Successful construction of the renal tubule cell ferroptosis model

After confirming the stability and stimulus-responsive properties of MHY@MO@PPP, we established an in vitro ferroptosis model in renal tubular cells to evaluate its biological activity. HK-2 cells were exposed to 1 µM Erastin, with untreated cells serving as the CON group and Erastin-treated cells serving as the Era group.

Erastin treatment produced the expected ferroptosis-associated phenotype in HK-2 cells. JC-1 staining showed loss of mitochondrial membrane potential in the Era group relative to CON cells (Figure S3A), and DCFH-DA staining showed higher intracellular ROS levels (Figure S3B). MDA increased and GSH decreased after Erastin exposure, indicating enhanced lipid peroxidation (Figure S3C-D). FerroOrange staining also showed increased intracellular free iron, supporting successful induction of the high-iron ferroptosis model (Figure S4A-B).

Cellular function was then assessed after the oxidative and iron-load changes had been verified. CCK-8 assays showed reduced viability in the Era group compared with CON cells (Figure S3E). Annexin V/PI flow cytometry detected a higher proportion of death-related cells after Erastin treatment (Figure S4C-D), providing functional evidence that the model impaired HK-2 cell survival under ferroptosis-inducing conditions.

We next examined molecular markers linked to ferroptosis, autophagy, and oxidative stress. Western blotting showed lower GPX4 and FTH1 expression and higher ACSL4 expression in Era-treated cells than in CON cells, indicating activation of ferroptosis-related molecular changes (Figure S5A). p-mTOR was reduced, whereas LC3II/I was increased, consistent with enhanced autophagy-associated stress (Figure S5B). SOD2 expression was also decreased in the Era group, further indicating oxidative damage during ferroptosis induction (Figure S5C).

The Erastin model also showed molecular features of tubular injury and profibrotic activation. RT-qPCR demonstrated increased KIM1, NGAL, and CYSC expression in the Era group compared with CON cells (Figure S5D). ACTA2, Fn1, and Vimentin were likewise elevated (Figure S5E), and confocal IF imaging showed stronger Fn1 signals after Erastin exposure (Figure S3F). These findings indicate that ferroptosis induction was accompanied by renal tubular injury and EMT/fibrosis-related changes.

### The MHY@MO@PPP nanocomplex significantly alleviates ferroptosis and kidney injury

**Fig. 2:**
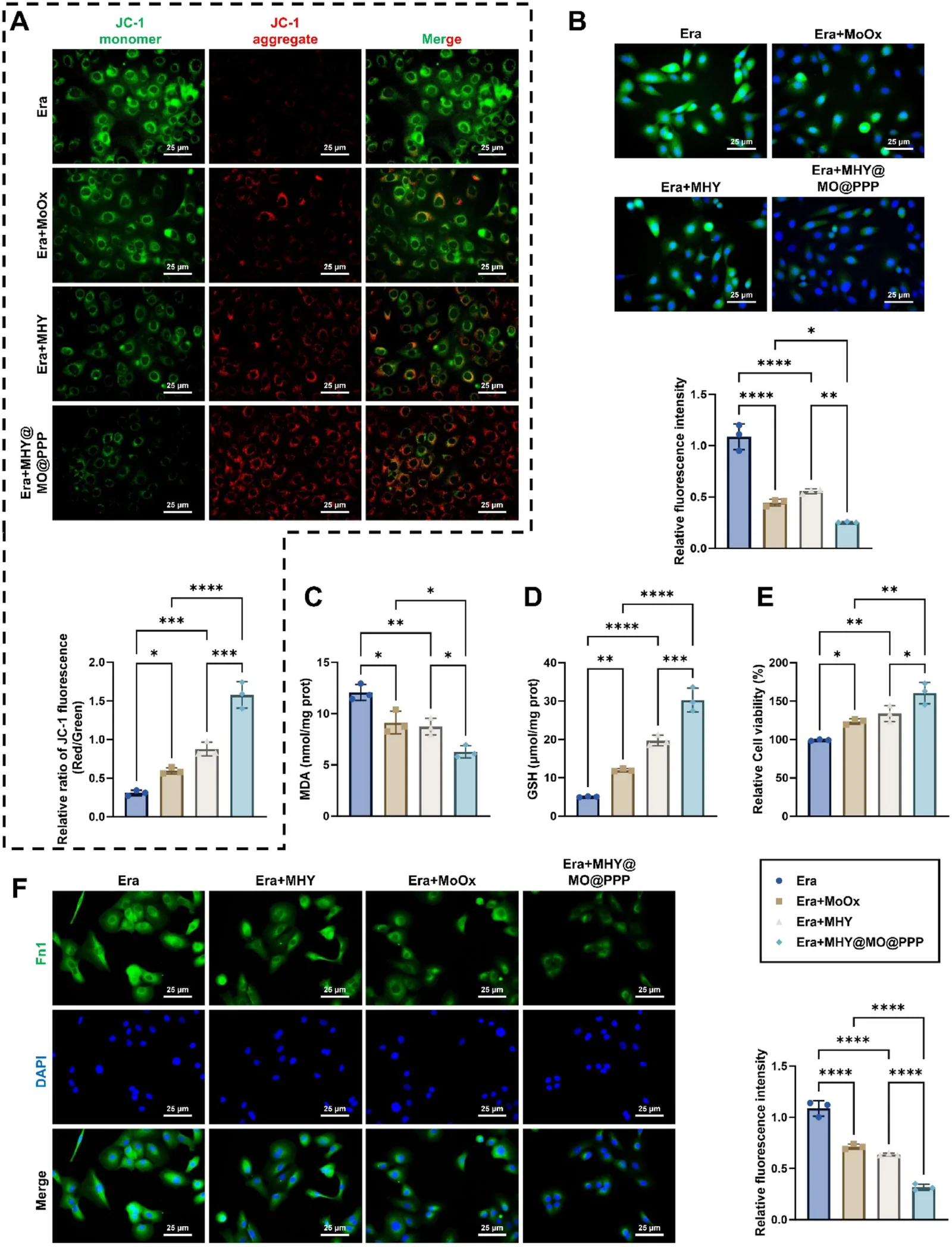
In vitro functional evaluation of MHY@MO@PPP nanocomposites in the HK-2 ferroptosis model. (**A**) JC-1 fluorescence staining to detect changes in mitochondrial membrane potential of HK-2 cells, bar: 25 μm; (**B**) DCFH-DA fluorescent probe to detect ROS generation levels in HK-2 cells, bar: 25 μm; (**C**) Biochemical kit detection of MDA content in HK-2 cells; (**D**) Biochemical kit detection of GSH content in HK-2 cells; (**E**) CCK-8 assay to detect changes in HK-2 cell viability; (**F**) IF staining combined with confocal laser scanning microscopy to detect the expression and distribution of Fibronectin protein in HK-2 cells, bar: 25 μm. The cell experiments were repeated three times. * Indicates comparison between two groups, *p <* 0.05, **p < 0.01, ***p < 0.001, ****p < 0.0001

Mo^5+^/Mo^6+^ valence cycling in MoOx supports ROS scavenging and oxidative-stress relief [14]. On this basis, we next tested the in vitro efficacy of the nanozyme platform in an erastin-induced ferroptosis model. HK-2 cells were assigned to the Era, Era + MHY, Era+MoOx, and Era + MHY@MO@PPP groups. JC-1 staining showed that mitochondrial membrane potential was restored in both the Era+MoOx and Era + MHY groups compared with the Era group, with a stronger recovery in the Era + MHY group; the most pronounced improvement was observed in the Era + MHY@MO@PPP group (Fig. 2 A). ROS detection showed a similar pattern: ROS generation decreased after MoOx or MHY treatment, was lower in the Era + MHY group than in the Era+MoOx group, and reached the lowest level in the Era + MHY@MO@PPP group (Fig. 2B). MDA and GSH assays further indicated that both interventions reduced lipid peroxidation and improved antioxidant capacity, with the composite formulation producing the strongest effect (Fig. 2C-D). CCK-8 analysis also showed improved cell viability in the Era+MoOx and Era + MHY groups relative to the Era group, and viability increased further after MHY@MO@PPP treatment (Fig. 2E).

The FerroOrange probe detection further demonstrated that iron ion accumulation was reduced in the Era + MHY group compared to the Era group, with the Era + MHY@MO@PPP group exhibiting the lowest iron load. In contrast, no significant difference was observed between the Era+MoOx group and the Era group (Figure S6A-B). Similarly, Annexin V/PI flow cytometry showed that the proportion of death-related Annexin V/PI-positive cells was decreased in the Era + MHY group compared with the Era group. The Era + MHY@MO@PPP group showed the most pronounced reduction in Annexin V/PI-positive cell death, whereas the Era+MoOx group did not differ significantly from the Era group (Figure S6C-D).

**Fig. 3:**
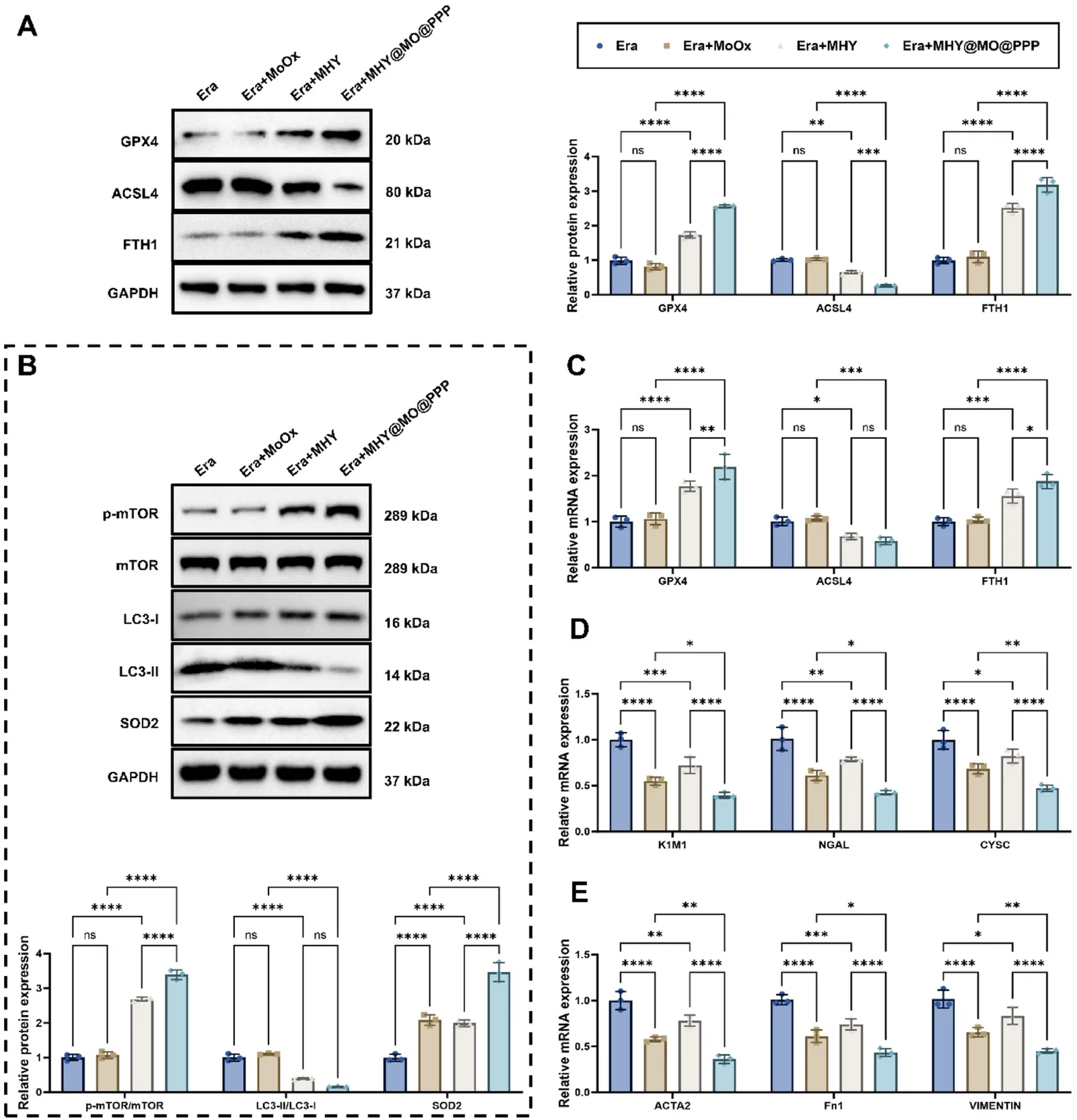
Regulatory effects of nanocomplexes on ferroptosis, autophagy/oxidative stress, and renal injury-fibrosis markers. (**A**) Western blot detection of ferroptosis-related protein (GPX4, ACSL4, FTH1) expression in HK-2 cells; (**B**) Western blot detection of mTOR-autophagy-oxidative stress-related proteins p-mTOR, LC3II/I, and SOD2; (**C**) RT-qPCR detection of ferroptosis-related transcripts GPX4, ACSL4, and FTH1; (**D**) RT-qPCR detection of renal injury markers KIM1, NGAL, and CYSC; (**E**) RT-qPCR detection of fibrosis/EMT markers ACTA2, Fn1, and VIMENTIN. Cell experiments were repeated three times. Comparisons were analyzed by one-way ANOVA followed by Tukey’s post hoc test unless otherwise specified. *p < 0.05, **p < 0.01, ***p < 0.001, ****p < 0.0001

We then assessed proteins associated with ferroptosis and autophagy signaling. Western blotting showed that, compared with the Era group, GPX4 and FTH1 were upregulated and ACSL4 was downregulated in the Era + MHY group. The Era + MHY@MO@PPP group showed the strongest regulation of these ferroptosis-related proteins, whereas the Era+MoOx group did not differ significantly from the Era group (Fig. 3 A). For autophagy-associated signaling, p-mTOR/mTOR increased and LC3II/I decreased in the Era + MHY group relative to the Era group; these changes were further enhanced in the Era + MHY@MO@PPP group. No significant differences in p-mTOR or LC3II/I were detected between the Era+MoOx and Era groups (Fig. 3B). For oxidative-stress-related proteins, SOD2 was upregulated in both the Era+MoOx and Era + MHY groups compared with the Era group, and the Era + MHY@MO@PPP group showed an additional increase compared with Era + MHY (Fig. 3B).

RT-qPCR results were consistent with the protein-level findings. Among ferroptosis-related transcripts, GPX4 and FTH1 increased and ACSL4 decreased in the Era + MHY group compared with the Era group. Relative to Era + MHY, MHY@MO@PPP further increased GPX4 and FTH1 expression and reduced ACSL4 expression, whereas Era+MoOx did not significantly alter GPX4, ACSL4, or FTH1 compared with Era alone (Fig. 3E). Transcripts associated with tubular injury, including KIM1, NGAL, and CYSC, were reduced in both the Era+MoOx and Era + MHY groups, with a larger decrease after MHY and the greatest decrease after MHY@MO@PPP treatment (Fig. 3 C). Fibrosis/EMT markers ACTA2, Fn1, and VIMENTIN showed the same treatment-responsive trend (Fig. 3D). Immunofluorescence analysis further showed that Fn1 fluorescence was strongest in the Era group, weakened after MoOx or MHY treatment, and was lowest in the Era + MHY@MO@PPP group (Fig. 2 F).

In conclusion, while individual interventions with MoOx and MHY alleviated ferroptosis-associated injury to varying degrees, the MHY@MO@PPP composite system showed the most pronounced protective effect under the tested in vitro conditions.

### Proteomics reveals the mechanism of ferroptosis-mTOR signaling reconstruction in HK-2 cells following nanomaterial intervention

**Fig. 4:**
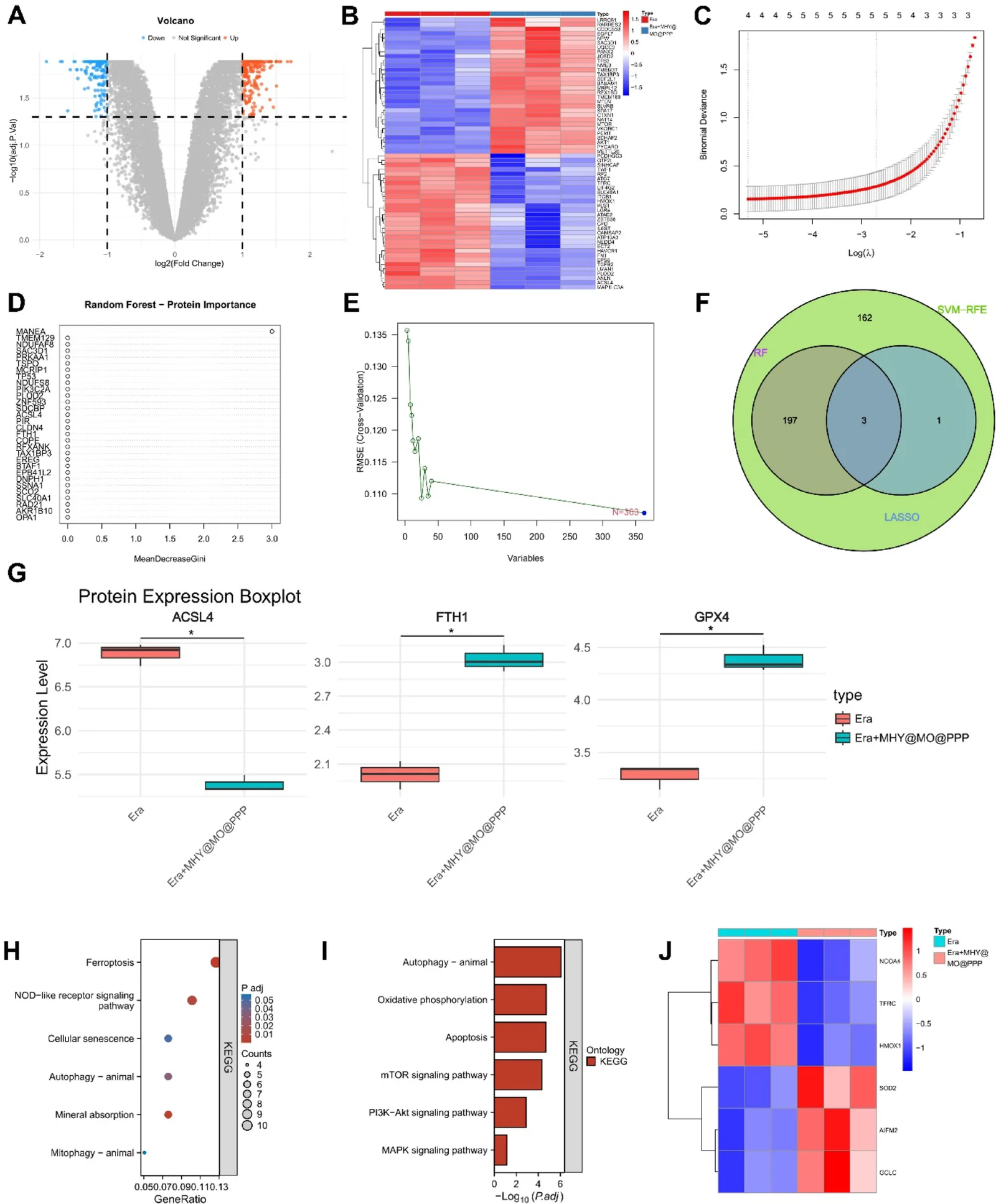
Proteomics and machine learning combined analysis of signal pathway characteristics after MHY@MO@PPP intervention. (**A**) Volcano plot of differential proteins detected by proteomics in HK-2 cells between the Era group and the Era + MHY@MO@PPP group. (**B**) Expression profile of differential proteins detected by proteomics in HK-2 cells between the two groups. (**C**) Characteristic variables of differential proteins in HK-2 cells screened by the LASSO regression model and cross-validation curve. (**D**) Random Forest model evaluating the importance ranking of differential proteins in HK-2 cells. (**E**) SVM-RFE screening for the optimal number of features and error changes of differential proteins in HK-2 cells. (**F**) Venn diagram showing common characteristic proteins screened by LASSO, Random Forest, and SVM-RFE. (**G**) Box plots comparing the expression levels of key proteins (ACSL4, FTH1, GPX4) in HK-2 cells between the two groups. (**H**) KEGG enrichment bubble chart showing pathway enrichment results of downregulated differential proteins in HK-2 cells. (**I**) KEGG bar chart showing pathway enrichment results of differential proteins in HK-2 cells. (**J**) Heatmap showing the expression profile clustering of representative proteins related to ferroptosis/oxidative stress in HK-2 cells between the two groups. Three samples per group. * indicates comparison between the two groups, *p <* 0.05, **p < 0.01, ***p < 0.001

After confirming that MHY@MO@PPP alleviated ferroptosis-associated cellular injury, we performed proteomic analysis to characterize molecular changes in HK-2 cells. Whole-proteome profiling was conducted in the Era and Era + MHY@MO@PPP groups. Quantitative mass spectrometry identified approximately 12,500 quantifiable protein groups across the LC-MS/MS runs. Using an FDR < 0.05 threshold, 363 differential proteins were identified, including 215 upregulated and 148 downregulated proteins in the Era + MHY@MO@PPP group (Fig. 4A-B). Biological interpretation was based on both the direction and magnitude of log2FC, with emphasis on pathway-level remodeling and consistently regulated ferroptosis-related proteins rather than minimal non-zero fold changes. Machine-learning feature selection further identified GPX4 and FTH1 as upregulated ferroptosis-suppressive proteins and ACSL4 as a downregulated ferroptosis-promoting protein after MHY@MO@PPP treatment (Fig. 4C-G). These findings indicate that the nanomaterial intervention attenuated the erastin-induced ferroptosis-related stress state.

KEGG enrichment analysis was used to interpret pathway-level changes among the differential proteins. Downregulated proteins were mainly associated with iron metabolism, autophagy activation, and oxidative-stress response modules, with enrichment in ferroptosis, autophagy, and oxidative-damage pathways (Fig. 4H). By contrast, upregulated proteins in the Era + MHY@MO@PPP group were enriched in mTOR signaling, cell-survival, and anti-stress regulatory networks. Together with the recovery trend of GPX4, these results support participation of the Fe^2+^-mTOR-GPX4 axis in MHY@MO@PPP-mediated relief of ferroptosis-associated stress (Fig. 4I). The enrichment heatmap also showed that several ferroptosis- and ROS-related proteins clustered at high expression in the Era group and decreased after MHY@MO@PPP treatment, indicating broad attenuation of the stress phenotype in the treated group (Fig. 4J).

Overall, MHY@MO@PPP restored GPX4 expression, weakened the iron-related lipid peroxidation cascade, reduced autophagy/ROS-associated stress, and increased mTOR pathway activity. These molecular changes provide mechanistic support for the observed reduction in Erastin-induced ferroptosis and renal tubular injury.

### Intervention in the Fe^2+^-mTORC1-GPX4 axis attenuates the ferroptosis phenotype

**Fig. 5:**
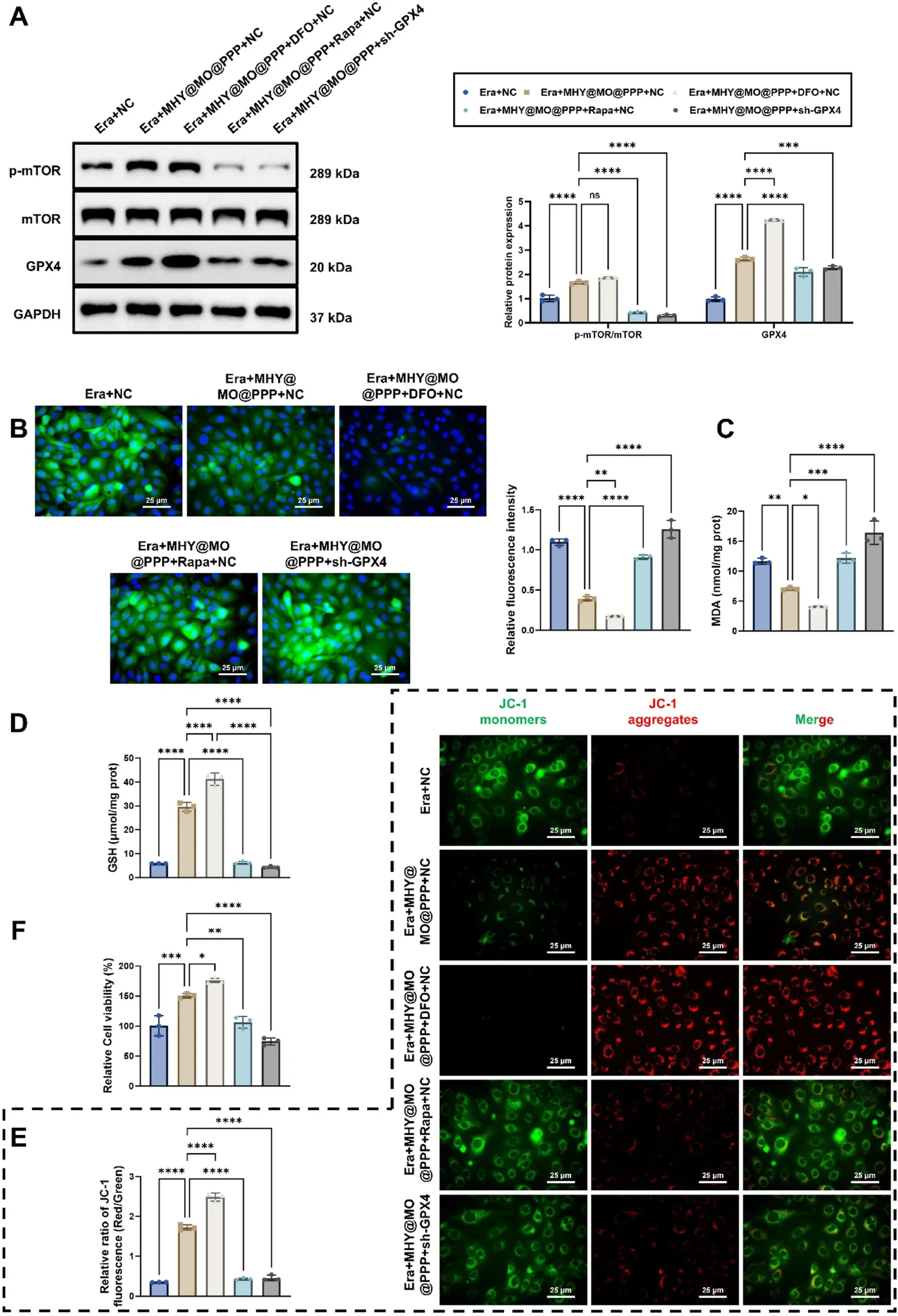
Verification of the role of the Fe²⁺-mTORC1-GPX4 signaling axis in nanocomplex therapy. (**A**) Western blot detection of GPX4 and p-mTOR/mTOR protein expression in HK-2 cells after DFO, rapamycin, or sh-GPX4 intervention; (**B**) DCFH-DA detection of intracellular ROS generation, scale bar: 25 μm; (**C**) MDA content detection to evaluate lipid peroxidation; (**D**) GSH measurement to evaluate antioxidant capacity; (**E**) JC-1 staining to assess mitochondrial membrane potential (MMP), scale bar: 25 μm; (**F**) CCK-8 assay to assess HK-2 cell viability. Cell experiments were repeated three times. Comparisons were analyzed by one-way ANOVA followed by Tukey’s post hoc test unless otherwise specified. *p < 0.05, **p < 0.01, ***p < 0.001, ****p < 0.0001

Based on the previous proteomics data indicating that MHY@MO@PPP regulates the Fe^2+^-mTOR-GPX4 signaling network, we further evaluated the functional contribution of this axis using shRNA and pharmacological blockade. Western blot results demonstrated that compared with the Era + MHY@MO@PPP + NC group, treatment with the iron chelator DFO further enhanced GPX4 expression and maintained elevated p-mTOR levels. Conversely, administration of the mTOR inhibitor rapamycin or transfection with sh-GPX4 significantly downregulated GPX4 and p-mTOR expression compared to the Era + MHY@MO@PPP + NC group (Fig. 5 A), indicating that mTORC1 signaling and GPX4 restoration are closely associated with the protective phenotype. These results support the Fe^2+^-mTOR-GPX4 signaling pathway as an important regulatory axis associated with the nanocomplex’s anti-ferroptosis effects, without establishing a strict linear hierarchy between mTORC1 activation and GPX4 restoration.

To verify functional consequences, we examined mitochondrial activity, oxidative status, and cell viability. DCFH-DA fluorescence imaging showed that ROS levels increased after rapamycin or sh-GPX4 intervention (Fig. 5B), accompanied by higher MDA content (Fig. 5 C) and lower GSH content (Fig. 5D). JC-1 analysis indicated that mitochondrial membrane potential decreased in the rapamycin and sh-GPX4 groups compared with the Era + MHY@MO@PPP + NC group (Fig. 5E). CCK-8 assays showed reduced cell viability after rapamycin treatment or GPX4 silencing (Fig. 5 F). FerroOrange staining further demonstrated increased iron load in these groups (Figure S7A-B), and Annexin V/PI analysis showed more Annexin V/PI-positive cells (Figure S7C-D). In combination with ferroptosis-related molecular and oxidative-stress markers, these results indicate that mTOR blockade or GPX4 silencing weakens the anti-ferroptotic activity of MHY@MO@PPP, supporting the mTORC1-GPX4 axis as an important component of its therapeutic effect.

Renal injury and fibrosis-related transcripts were also examined by RT-qPCR. KIM1, NGAL, and CYSC were higher in the rapamycin and sh-GPX4 groups than in the Era + MHY@MO@PPP + NC group (Figure S7E), and ACTA2, Fn1, and VIMENTIN showed a similar increase (Figure S7F). These results indicate that inhibition of mTOR signaling or GPX4 knockdown weakens the ability of MHY@MO@PPP to suppress injury- and fibrosis-associated transcriptional responses, supporting the Fe^2+^-mTORC1-GPX4 profile as a key contributor to the protective phenotype.

### MHY@MO@PPP nanocomplex exhibits favorable biosafety and targeting ability

**Fig. 6:**
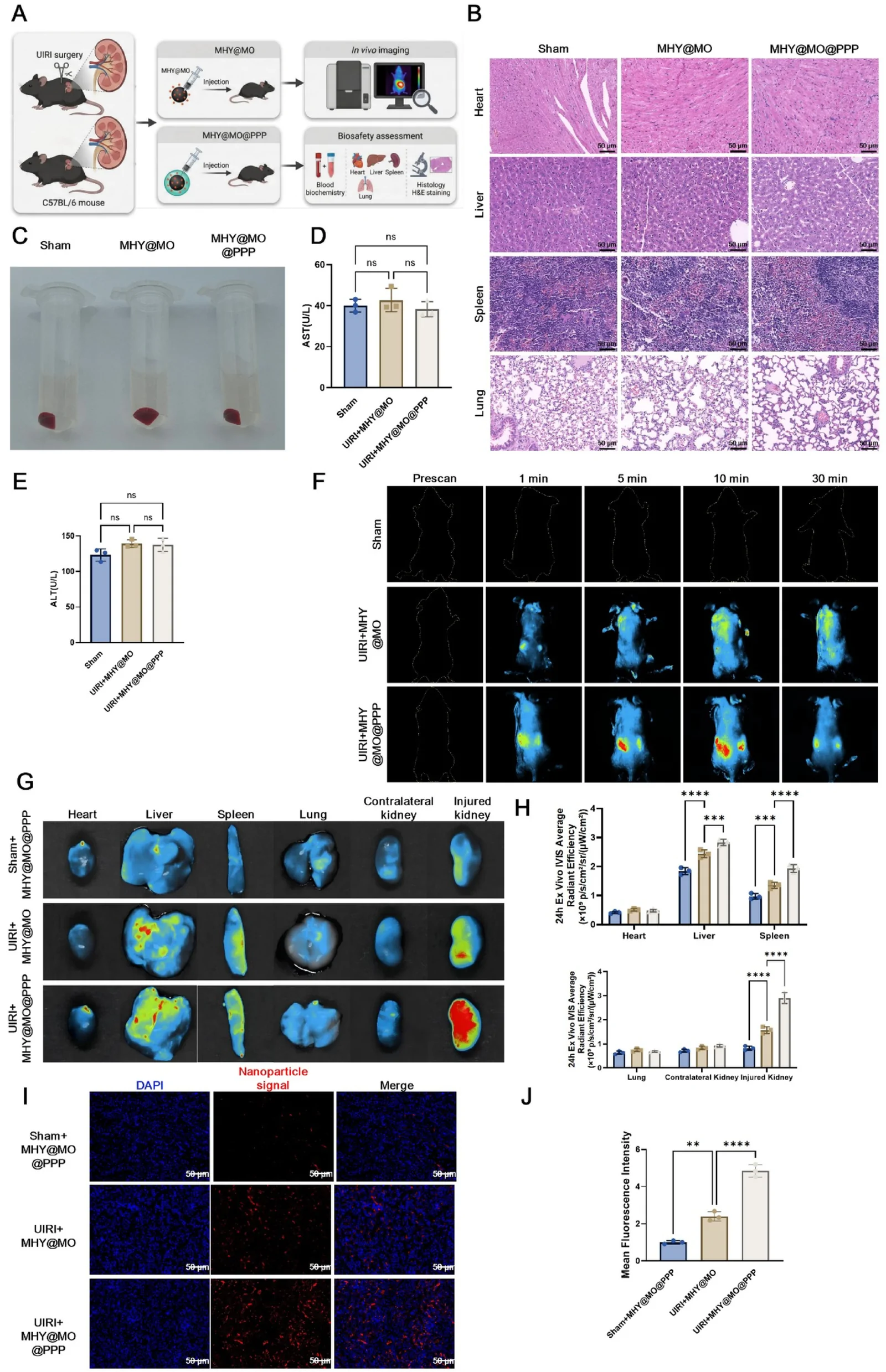
In vivo imaging, 24 h ex vivo biodistribution, renal-section localization, and biosafety assessment of MHY@MO@PPP. (**A**) Schematic diagram illustrating the experimental workflow for in vivo imaging, ex vivo biodistribution, renal-section localization, and biosafety assessment; (**B**) H&E staining of major organs (heart, liver, spleen, lung) to evaluate systemic toxicity, scale bar: 50 μm; (**C**) hemolysis assay results, including quantitative hemolysis ratio; (**D**) AST detection after the final administration; (**E**) ALT detection after the final administration; (**F**) dynamic NIR in vivo imaging showing the short-term distribution of nanomaterials at Prescan, 1, 5, 10, and 30 min; (**G**-**H**) 24 h ex vivo IVIS imaging and quantitative fluorescence analysis of major organs, including heart, liver, spleen, lung, contralateral kidney, and injured kidney; (**I**-**J**) renal-section LSCM imaging and quantitative fluorescence analysis at 24 h post-injection. Comparisons were analyzed by one-way ANOVA followed by Tukey’s post hoc test. *p < 0.05, **p < 0.01, ***p < 0.001, ****p < 0.0001

After the in vitro mechanism had been characterized, we performed in vivo experiments to evaluate the biosafety, short-term NIR distribution, 24 h organ biodistribution, and renal tissue localization of the nanocomplex. Sham mice received MHY@MO or MHY@MO@PPP for preliminary biosafety assessment, whereas UIRI mice were used to examine renal accumulation after nanomaterial administration (Fig. 6 A).

For biosafety assessment, major organs were collected and examined histologically. H&E staining showed preserved morphology in the heart, liver, spleen, and lung across the tested groups (Fig. 6B). The hemolysis assay revealed no obvious hemocompatibility concern (Fig. 6 C). Serum AST and ALT levels were comparable among the Sham, MHY@MO, and MHY@MO@PPP groups, indicating no evident short-term liver toxicity under the tested regimen (Fig. 6D-E).

NIR in vivo imaging was used to assess short-term nanomaterial distribution over 0–30 min. Across the Prescan, 1, 5, 10, and 30 min imaging window, the UIRI + MHY@MO@PPP group displayed a relatively concentrated fluorescence signal in the renal region, whereas the UIRI + MHY@MO group showed a weaker or more dispersed renal-region signal and the Sham/UIRI controls showed low background signals (Fig. 6 F). These data support short-term renal fluorescence enrichment and were interpreted together with the 24 h organ and tissue imaging experiments below.

To further evaluate renal accumulation at a later time point, major organs were collected at 24 h after intravenous injection and analyzed by ex vivo IVIS imaging. The injured kidney in the UIRI + MHY@MO@PPP group exhibited the strongest fluorescence signal, whereas the UIRI + MHY@MO group showed a moderate renal signal and the Sham + MHY@MO@PPP group showed lower renal fluorescence (Fig. 6G-H). Detectable fluorescence in reticuloendothelial organs, including the liver and spleen, was consistent with the general biodistribution pattern of nanomaterials. Quantitative organ analysis further supported enhanced accumulation of MHY@MO@PPP in the injured kidney region at 24 h post-injection.

Renal-section LSCM analysis at 24 h post-injection further confirmed tissue-level localization. The UIRI + MHY@MO@PPP group showed the strongest fluorescence signal in kidney sections, whereas the UIRI + MHY@MO and Sham + MHY@MO@PPP groups displayed weaker signals (Fig. 6I-J). Together, these findings support enhanced renal accumulation and tissue localization of MHY@MO@PPP in the UIRI model, although they should be interpreted as renal-enrichment evidence rather than definitive proof of lesion-specific targeting or long-term retention.

Taken together, the imaging and safety results indicate acceptable preliminary biosafety, short-term renal-region fluorescence enrichment within the 0–30 min window, and strengthened 24 h evidence for renal accumulation and kidney-section localization of MHY@MO@PPP. short-term NIR imaging, 24 h ex vivo organ imaging, renal-section LSCM validation, indicate MHY@MO@PPP exhibits renal-region fluorescence enrichment, enhanced 24 h accumulation injured tissue, acceptable preliminary under the tested dosing regimen.

### MHY@MO@PPP nanocomposites ameliorate renal injury and the progression of fibrosis

**Fig. 7:**
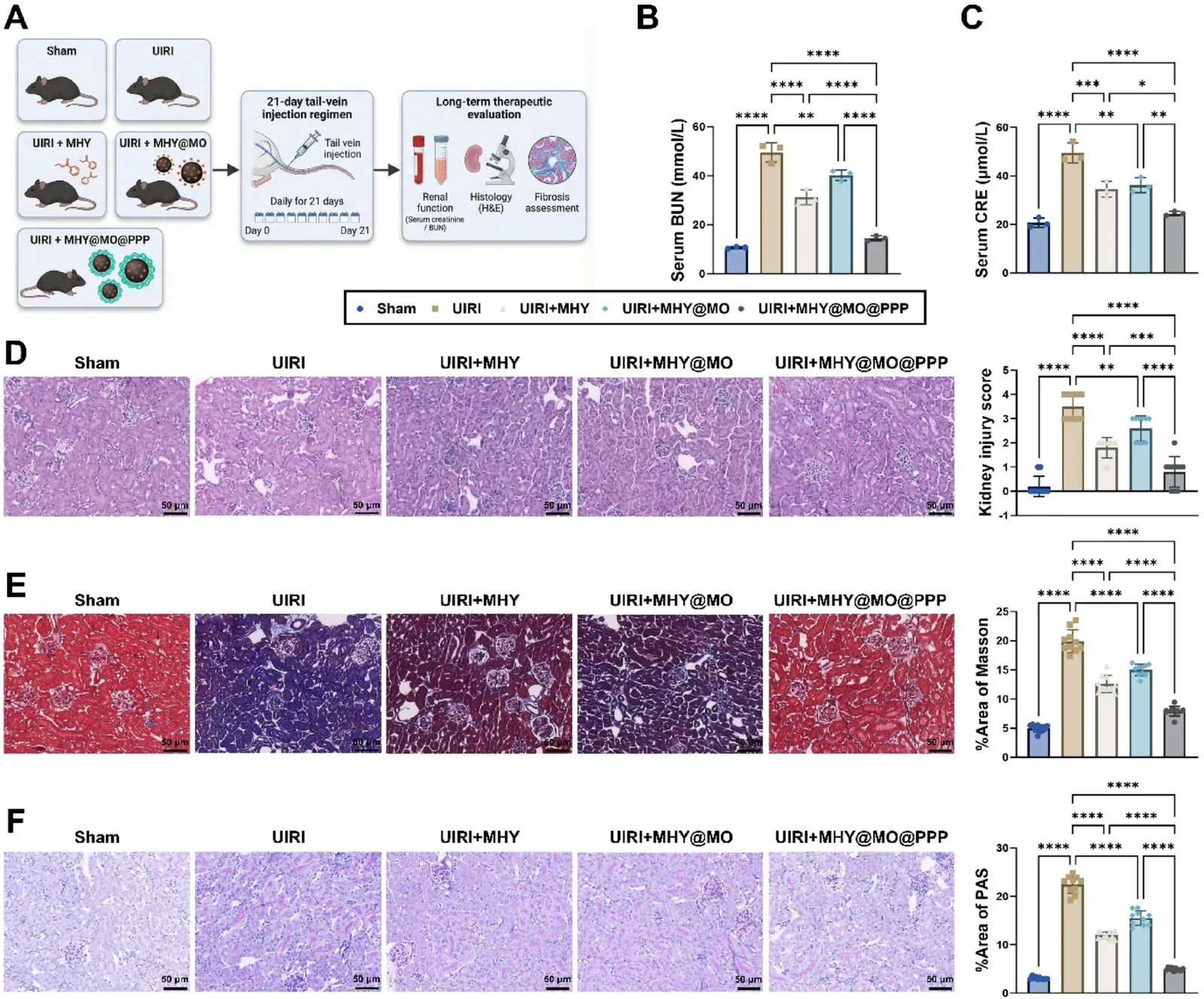
Evaluation of MHY@MO@PPP on renal function and histopathology in the UIRI model. (**A**) Schematic diagram showing the intervention procedure for the AKI-to-CKD transition model in UIRI mice; (**B**-**C**) Blood urea nitrogen (BUN) and creatinine (CRE) levels used to evaluate renal injury; (**D**) H&E staining to evaluate renal tubular structural damage, scale bar: 50 μm; (**E**) Masson staining to evaluate interstitial collagen deposition, scale bar: 50 μm; (**F**) PAS staining to evaluate brush-border integrity and basement-membrane status, scale bar: 50 μm. *n* = 10 mice per group. Comparisons were analyzed by one-way ANOVA followed by Tukey’s post hoc test. *p < 0.05, **p < 0.01, ***p < 0.001, ****p < 0.0001

The therapeutic effect of MHY@MO@PPP was then evaluated in the AKI-to-CKD UIRI model (Fig. 7 A). BUN and CRE were elevated in the UIRI group compared with Sham mice, confirming renal dysfunction. Both UIRI + MHY and UIRI + MHY@MO showed partial decreases in these indicators, whereas UIRI + MHY@MO@PPP produced the strongest reduction (Fig. 7B-C). Histological analyses further supported this protective effect: H&E staining showed tubular disruption and necrosis after UIRI, Masson staining showed increased collagen deposition, and PAS staining revealed brush-border damage and basement-membrane thickening. These abnormalities were alleviated after treatment, with the most evident preservation of tubular architecture and reduction in collagen deposition in the UIRI + MHY@MO@PPP group (Fig. 7D-F).

**Fig. 8:**
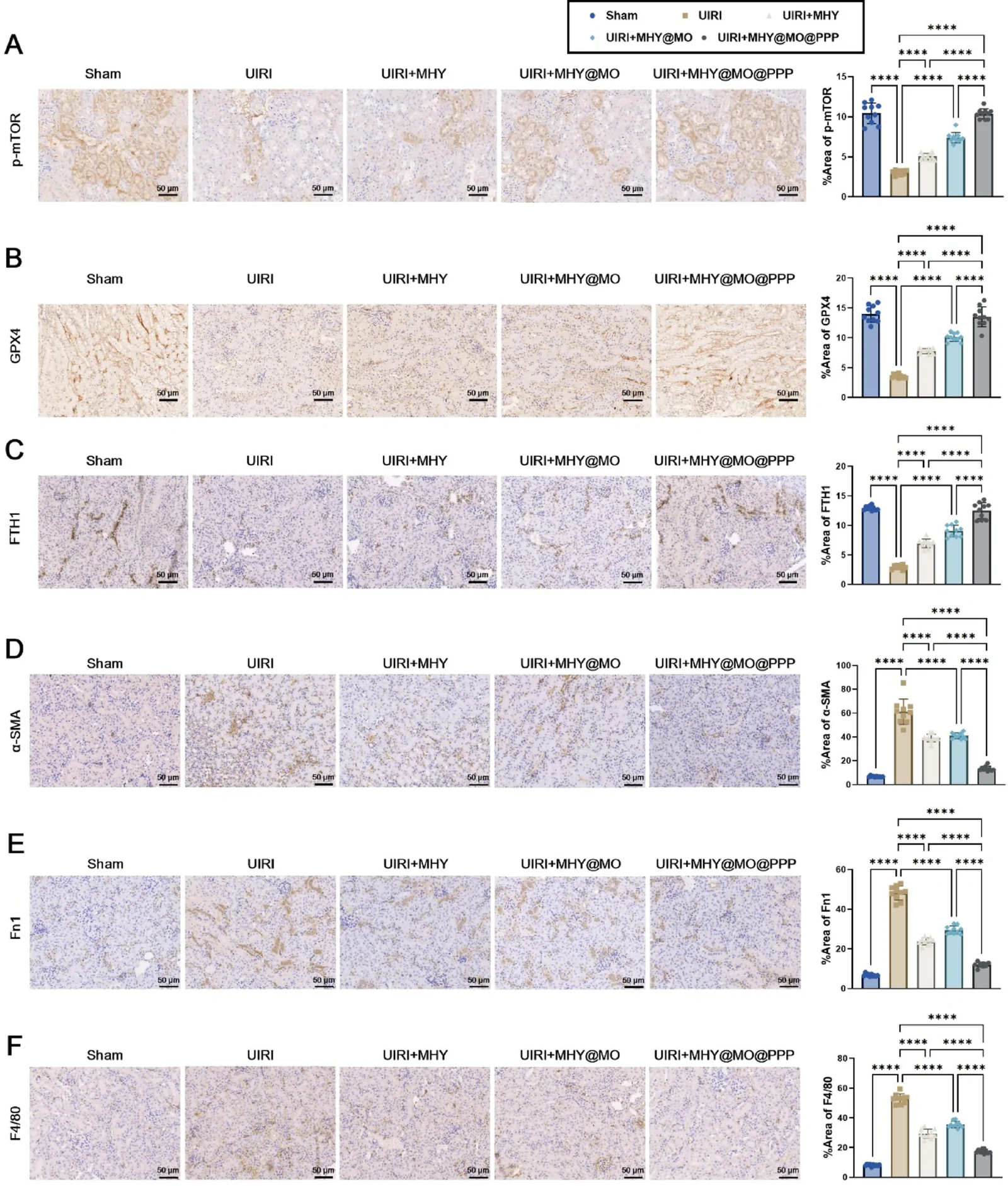
Histological verification of the Fe²⁺-mTORC1-GPX4-related protein profile and fibrosis/inflammation markers in the UIRI model. (**A**) IHC detection of p-mTOR protein expression and localization in renal tissue, scale bar: 50 μm; (**B**) IHC detection of GPX4 protein expression and localization in renal tissue, scale bar: 50 μm; (**C**) IHC detection of FTH1 protein expression and localization in renal tissue, scale bar: 50 μm; (**D**) IHC detection of α-SMA protein expression and localization in renal tissue, scale bar: 50 μm; (**E**) IHC detection of Fn1 protein expression and localization in renal tissue, scale bar: 50 μm; (**F**) IHC detection of F4/80 protein expression and localization in renal tissue, scale bar: 50 μm. *n* = 10 animals per group. Comparisons were analyzed by one-way ANOVA followed by Tukey’s post hoc test. *p < 0.05, **p < 0.01, ***p < 0.001, ****p < 0.0001

To investigate the molecular basis of the therapeutic effects, we further assessed key protein expression changes via IHC. The positive areas of p-mTOR, GPX4, and FTH1 were lower in the UIRI group than in the Sham group and were restored to varying degrees after intervention, with the most pronounced restoration in the UIRI + MHY@MO@PPP group (Fig. 8A-C). This pattern is consistent with the in vitro direction and supports an association between MHY@MO@PPP treatment and restoration of the Fe²⁺-mTORC1-GPX4-related protein profile, without establishing a strict upstream-downstream hierarchy in vivo. In contrast, the fibrosis markers α-SMA and Fn1 were upregulated in the UIRI group but reduced after MHY@MO@PPP treatment (Fig. 8D-E). A similar downward trend was observed for the inflammatory macrophage marker F4/80 (Fig. 8 F), suggesting that the nanozyme composite treatment suppresses inflammation- and fibrosis-associated histological changes.

In summary, the in vivo data show that MHY@MO@PPP improves renal function, reduces tissue damage and fibrosis, and is accompanied by recovery of p-mTOR, GPX4, and FTH1 expression. These results support a contribution of the Fe^2+^-mTORC1-GPX4-related protein profile to the protective effect in the AKI-to-CKD transition model, while not proving a strict linear pathway in vivo.

## Discussion

This study developed an MHY@MO@PPP nanocomposite system centered on the Fe^2+^-mTORC1-GPX4 regulatory axis. In erastin-induced HK-2 cell ferroptosis and the UIRI-induced AKI-to-CKD model, MHY@MO@PPP improved oxidative-stress status, reduced free Fe^2+^ burden, and attenuated ferroptosis-related phenotypes, accompanied by increased mTORC1 activity and restored GPX4 expression. These observations are consistent with the role of ferroptosis in AKI-related cell injury [5]. Whereas many previous studies have focused on acute injury endpoints, evidence for a sustained ferroptosis-amplifying loop during AKI-to-CKD transition remains limited. By examining both renal functional indices and fibrotic remodeling in the UIRI chronicity model, our data align with the concept that AKI-to-CKD progression is driven by persistent tubular damage and maladaptive repair [1]. Thus, the results are best interpreted as evidence that suppressing ferroptosis-related stress may improve the post-AKI injury microenvironment. Proteomics and machine-learning analyses further indicated broad remodeling of cellular stress networks after intervention, although these findings remain model-based and require validation in additional systems.

For iron homeostasis, MHY@MO@PPP reduced Fe^2+^ levels and restored related indicators such as FTH1, suggesting partial correction of cellular iron imbalance. Prior work has shown that disrupted iron handling and ferritin autophagy can amplify ferroptosis in ischemia-reperfusion-associated AKI by increasing free Fe^2+^ [8]. In the present study, DFO intervention functionally supported the importance of Fe^2+^ in the protective response, but the specific source of iron mobilization was not dissected. This interpretation fits the multi-node nature of nanocomposite interventions, in which redox regulation, iron burden, and signaling changes may occur simultaneously. The present study did not directly determine whether altered Fe^2+^ levels originated from iron uptake, storage, or autophagy; thus, the upstream regulation of iron homeostasis should be interpreted cautiously. Overall, the findings are consistent with the view that Fe^2+^ accumulation promotes ferroptosis and aggravates renal injury, while leaving the precise upstream regulatory mechanism for future study.

GPX4 is a central defense molecule against ferroptosis. In this study, GPX4 expression increased in parallel with improvements in ferroptosis-related indicators, and GPX4 silencing markedly weakened the protective effect of MHY@MO@PPP. These results support a key role for GPX4 in the anti-ferroptotic activity of the composite system. Previous studies have shown that reduced GPX4 stability increases ferroptosis sensitivity in renal tubular cells; for example, TRIM21-mediated GPX4 ubiquitination aggravates ischemic AKI [7]. Other reports also link GPX4 inhibition with AKI-to-CKD progression [6]. Compared with those studies, our work emphasizes the association between GPX4 restoration and overall relief of the ferroptotic phenotype, but it does not define the molecular source of GPX4 upregulation. In addition, GPX4 silencing may alter basal antioxidant capacity, so the magnitude of treatment differences should be interpreted with appropriate caution.

The mTORC1 pathway changed in a pattern consistent with GPX4 in this study. Previous evidence indicates that mTORC1 influences cellular antioxidant capacity and GPX4 protein synthesis, thereby regulating susceptibility to ferroptosis [10]. Here, MHY1485-containing treatment increased p-mTOR levels, whereas rapamycin weakened the protective phenotype, supporting participation of mTORC1 in the Fe^2+^-mTORC1-GPX4-related response. However, these data should not be read as definitive proof of a linear in vivo hierarchy from mTORC1 activation to GPX4 restoration. mTOR signaling in renal injury is context-dependent and may differ by injury stage and cell type. Therefore, the observed mTORC1 changes are more appropriately interpreted as part of a protective adaptation to ferroptotic stress rather than as a universal mechanism across all renal injury settings. The combination of MHY1485 with a nanozyme also differs from small-molecule treatment alone, suggesting potential interaction between pathway regulation and redox modulation.

Regarding autophagy-related stress, the observed changes in LC3II/I were consistent with the trend of ferroptosis mitigation, consistent with the perspective that autophagy may facilitate ferroptosis amplification. Previous studies have demonstrated that in ischemic AKI, specific types of autophagy, such as ferritinophagy, can mobilize iron stores and promote ferroptosis ([8]). Concurrently, some studies indicate that autophagy-related processes may influence GPX4 stability ([9]). However, the role of autophagy in AKI is dualistic, potentially exerting protective effects or contributing to injury amplification depending on the distinct stages or stress contexts. Therefore, this study does not interpret autophagy alterations as the sole mechanism but rather discusses them as an integral component of the ferroptosis network.

From a materials perspective, the platform combines oxygen-vacancy-containing MoOx with MHY1485. Molybdenum-based nanomaterials have reported ROS-scavenging activity and potential utility in AKI treatment [12]. Unlike strategies that rely only on nanozyme antioxidant activity, MHY@MO@PPP integrates redox regulation, MHY1485 delivery, and pH-responsive release. Table S3 compares representative nanozyme-based AKI strategies and highlights that the present platform links ROS scavenging, responsive MHY1485 delivery, mTORC1 activation, GPX4 restoration, and AKI-to-CKD fibrosis-related endpoints. The FBS incubation results also suggest that serum protein adsorption or protein-corona formation may mildly change effective particle size and surface charge, which should be considered when interpreting in vivo biodistribution. Nevertheless, pharmacokinetics, long-term safety, and detailed protein-corona composition have not yet been systematically characterized; therefore, the translational potential of the material system requires further evaluation.

This study has several limitations. First, the evidence was obtained mainly from in vitro cell experiments and a single animal model, without validation in human tissues or clinical samples; interspecies differences may therefore affect extrapolation. Second, although proteomics and machine-learning screening helped define network alterations, the stability of these findings may be influenced by sample size and batch effects and should be tested in independent datasets. Third, the study did not precisely localize autophagy subtypes or identify the sources of altered iron homeostasis, leaving mechanistic resolution incomplete. Fourth, the in vivo experiments did not include a bare MoOx-alone efficacy group, systematic pharmacokinetic analysis, long-term clearance evaluation, or detailed protein-corona proteomic profiling, limiting direct attribution of component-specific efficacy, long-term retention, and blood-protein interaction mechanisms. Fifth, ACSL4 was identified as a ferroptosis-associated protein in the in vitro proteomic analysis, but direct validation of ACSL4 expression in UIRI kidney tissue remains lacking. Future studies should validate these findings in additional AKI models and larger cohorts, include component-specific in vivo controls, and use spatial, single-cell, or proteomic approaches to clarify the roles of the Fe^2+^-mTORC1-GPX4 axis and material-biointerface interactions in distinct renal tubular subpopulations.

## Supplementary Information

Supplementary Material 1: Figure S1. Construction process and physicochemical property analysis of MoOx nanozymes and protein-environment stability of MHY@MO@PPP. Note: (A) Schematic diagram illustrating the construction process of MoOx nanozymes; (B) TEM observation of the overall morphology and dispersion state of MoOx nanozymes, scale bar: 500 nm; (C) DLS analysis of MoOx nanozymes; (D) FT-IR detection of the chemical bond structure and surface functional group characteristics of MoOx nanozymes; (E) XPS analysis of the chemical valence composition of Mo (Mo⁶⁺/Mo⁵⁺) in MoOx nanozymes, used to evaluate oxygen-vacancy structural characteristics; (F) UV-Vis-NIR absorption spectrum detection of the light absorption characteristics of MoOx nanozymes in the visible to NIR-II range; (G) continuous monitoring for 30 days at 4°C to observe the storage dispersibility of the nanozymes; (H) DLS detection of the hydrodynamic size distribution of MHY@MO@PPP after incubation in PBS or PBS+10% FBS; (I) Zeta potential detection of MHY@MO@PPP after incubation in PBS or PBS+10% FBS; (J) DLS monitoring of MHY@MO@PPP hydrodynamic diameter in PBS+10% FBS at 0, 6, 12, and 24 h to evaluate short-term colloidal stability in a protein-containing environment. All experiments were independently repeated three times, and quantitative data are presented as mean ± SD where applicable. Figure S2. Encapsulation and release characteristics of MHY1485 in MHY@MO@PPP nanozyme complex. Note: (A) LC-MS/MS detection of the characteristic peak of MHY1485 in the MHY@MO@PPP nanozyme complex to verify the encapsulation of MHY1485; (B) LC-MS/MS detection of the release behavior of MHY1485 from the MHY@MO@PPP nanozyme complex under different pH conditions; (C) LC-MS/MS detection of the release behavior of MHY1485 from the MHY@MO@PPP nanozyme complex under different H_2_O_2_ stimulation conditions; (D) Laser scanning microscopy observation of the uptake of nanomaterials by HK-2 cells; red fluorescence represents Dil, and blue is DAPI nuclear staining (scale bar: 25 μm). Figure S3. Construction and phenotypic analysis of the HK-2 cell ferroptosis model under a high iron environment. Note: (A) JC-1 fluorescence staining detected mitochondrial membrane potential changes in HK-2 cells, bar: 25 μm; (B) DCFH-DA fluorescence probe detected intracellular ROS generation levels in HK-2 cells, bar: 25 μm; (C) Biochemical kit detected intracellular MDA content in HK-2 cells; (D) Biochemical kit detected intracellular GSH content in HK-2 cells; (E) CCK-8 assay detected changes in HK-2 cell viability; (F) IF staining combined with laser confocal microscopy detected the expression and distribution of Fibronectin protein in HK-2 cells, bar: 25 μm. Cell experiments were repeated three times. * indicates comparison between two groups, *p <* 0.05, **p < 0.01, ***p < 0.001. Figure S4. Analysis of iron homeostasis dysregulation and cell death characteristics in the ferroptosis model. Note: (A-B) FerroOrange fluorescent staining detection of intracellular free iron levels in HK-2 cells (A) and quantitative analysis results (B), bar: 25 μm; (C) Flow cytometry Annexin V/PI staining detection of HK-2 cell death proportion; (D) Quantitative analysis of PI-positive cell distribution in HK-2 cells by flow cytometry. Cell experiments were repeated three times. * indicates comparison between two groups, *p <* 0.05, ***p < 0.001. Figure S5. Analysis of molecular expression profiles and renal injury markers in the ferroptosis model. Note: (A) Western blot detection of ferroptosis-related proteins (GPX4, ACSL4, FTH1) expression in HK-2 cells; (B) Western blot detection of autophagy-related proteins p-mTOR and LC3II/I expression in HK-2 cells; (C) Western blot detection of oxidative stress-related protein SOD2 expression in HK-2 cells; (D) RT-qPCR detection of mRNA expression of renal tubular injury markers KIM1, NGAL, and CYSC in HK-2 cells; (E) RT-qPCR detection of mRNA expression of fibrosis and EMT-related genes ACTA2, Fn1, and VIMENTIN in HK-2 cells. Cellular experiments were repeated three times. * indicates comparison between two groups, *p <* 0.05, **p < 0.01, ***p < 0.001, ****p < 0.0001. Figure S6. Effects of MHY@MO@PPP nanocomposites on iron ion homeostasis and ferroptosis in cells. Note: (A-B) FerroOrange detection of Fe^2+^ levels in HK-2 cells, bar: 25 μm; (C-D) Annexin V/PI flow cytometry detection of Annexin V/PI-positive cell death proportion. Cell experiments were repeated three times. Comparisons were analyzed by one-way ANOVA followed by Tukey's post hoc test. *p < 0.05, **p < 0.01, ***p < 0.001. Figure S7. Detection of cellular Fe^2+^ load, death-related phenotypes, and renal injury/fibrosis markers after intervention of the Fe^2+^-mTORC1-GPX4 axis. Note: (A-B) FerroOrange detection of Fe^2+^ levels in HK-2 cells, scale bar: 25 μm; (C-D) Annexin V/PI flow cytometry detection of death-related Annexin V/PI-positive cells; (E) RT-qPCR detection of renal injury markers KIM1, NGAL, and CYSC; (F) RT-qPCR detection of fibrosis/EMT markers ACTA2, Fn1, and VIMENTIN. Cell experiments were repeated three times. * indicates comparison between two groups, *p <* 0.05, **p < 0.01, ***p < 0.001.

Supplementary Material 2

Supplementary Material 3

## Data Availability

All data generated or analyzed during this study are included in this article and/or its supplementary material files. Further enquiries can be directed to the corresponding author.

## Abbreviations

AKI: Acute kidney injury
ALT: Alanine aminotransferase
AST: Aspartate aminotransferase
BUN: Blood urea nitrogen
CKD: Chronic kidney disease
CRE: Creatinine
DFO: Deferoxamine
DLS: Dynamic light scattering
EMT: Epithelial-mesenchymal transition
FC: Fold change
FDR: False discovery rate
FT-IR: Fourier transform infrared spectroscopy
GSH: Glutathione
H&E: Hematoxylin and eosin
IF: Immunofluorescence
IHC: Immunohistochemistry
KIM1: Kidney injury molecule-1
LASSO: Least absolute shrinkage and selection operator
LC-MS/MS: Liquid chromatography-tandem mass spectrometry
MDA: Malondialdehyde
MHY@MO@PPP: MHY1485-loaded MoOx nanozyme coated with a pH-responsive PBA-PEG-PBA polymer
NIR: Near-infrared
PAS: Periodic acid-schiff
ROIs: Regions of interest
ROS: Reactive oxygen species
SVM-RFE: Support vector machine-recursive feature elimination
TEM: Transmission electron microscopy
UIRI: Unilateral ischemia-reperfusion injury
UV-Vis: Ultraviolet-visible
XPS: X-ray photoelectron spectroscopy
