# Supplementary material for: MHY@MO@PPP inhibits ferroptosis positive feedback and delays the acute kidney injury-to-chronic kidney disease transition by remodeling the Fe2+-mTORC1-GPX4 axis
Source: J Nanobiotechnology. 2026 Aug 20;24:914. doi: 10.1186/s12951-026-04918-w (PMC13625235; doi:10.1186/s12951-026-04918-w)
Supplement: Supplementary file 1 [file 12951_2026_4918_MOESM2_ESM.pdf]

Supplementary Figure S1

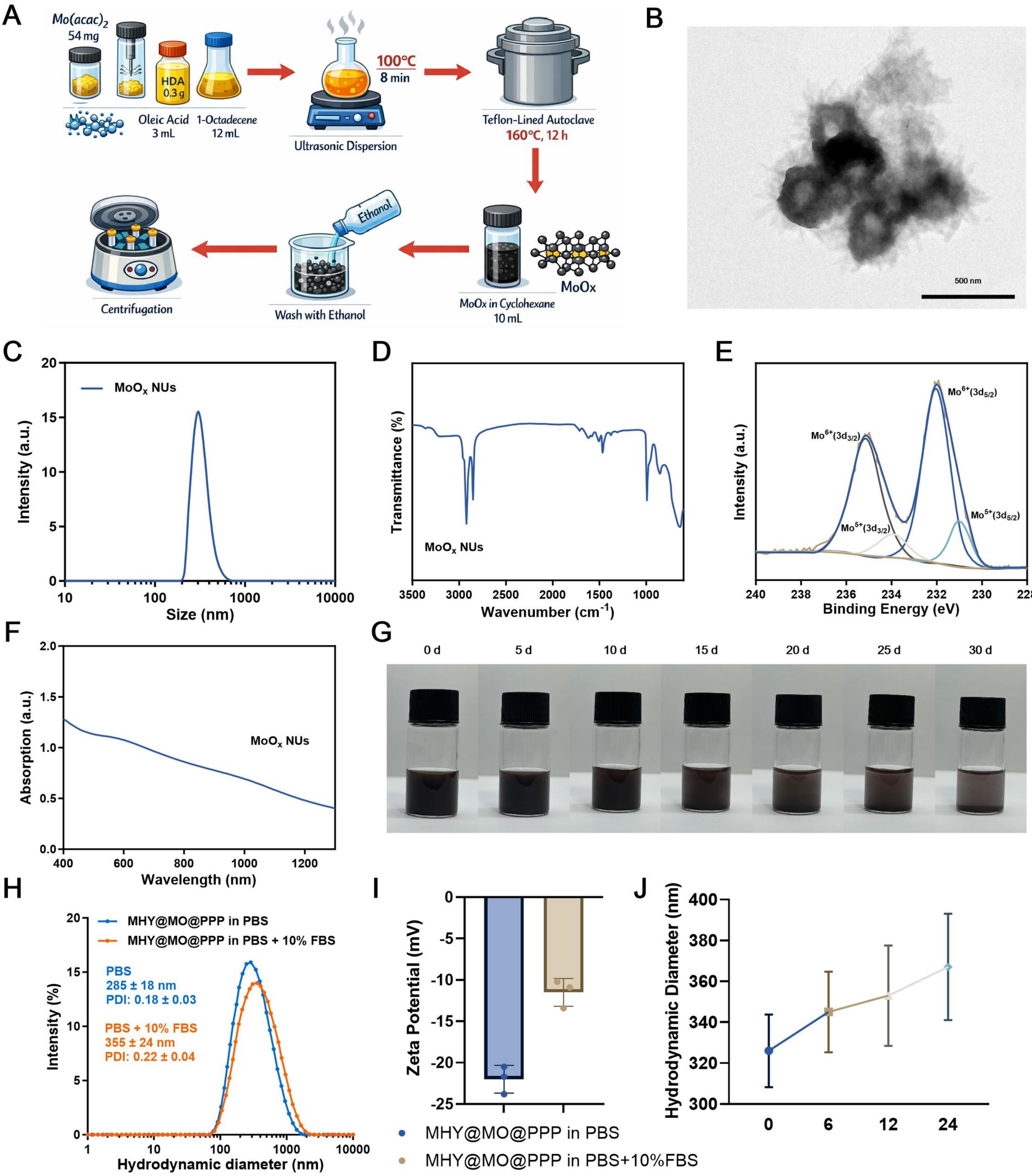

Supplementary Figure S2

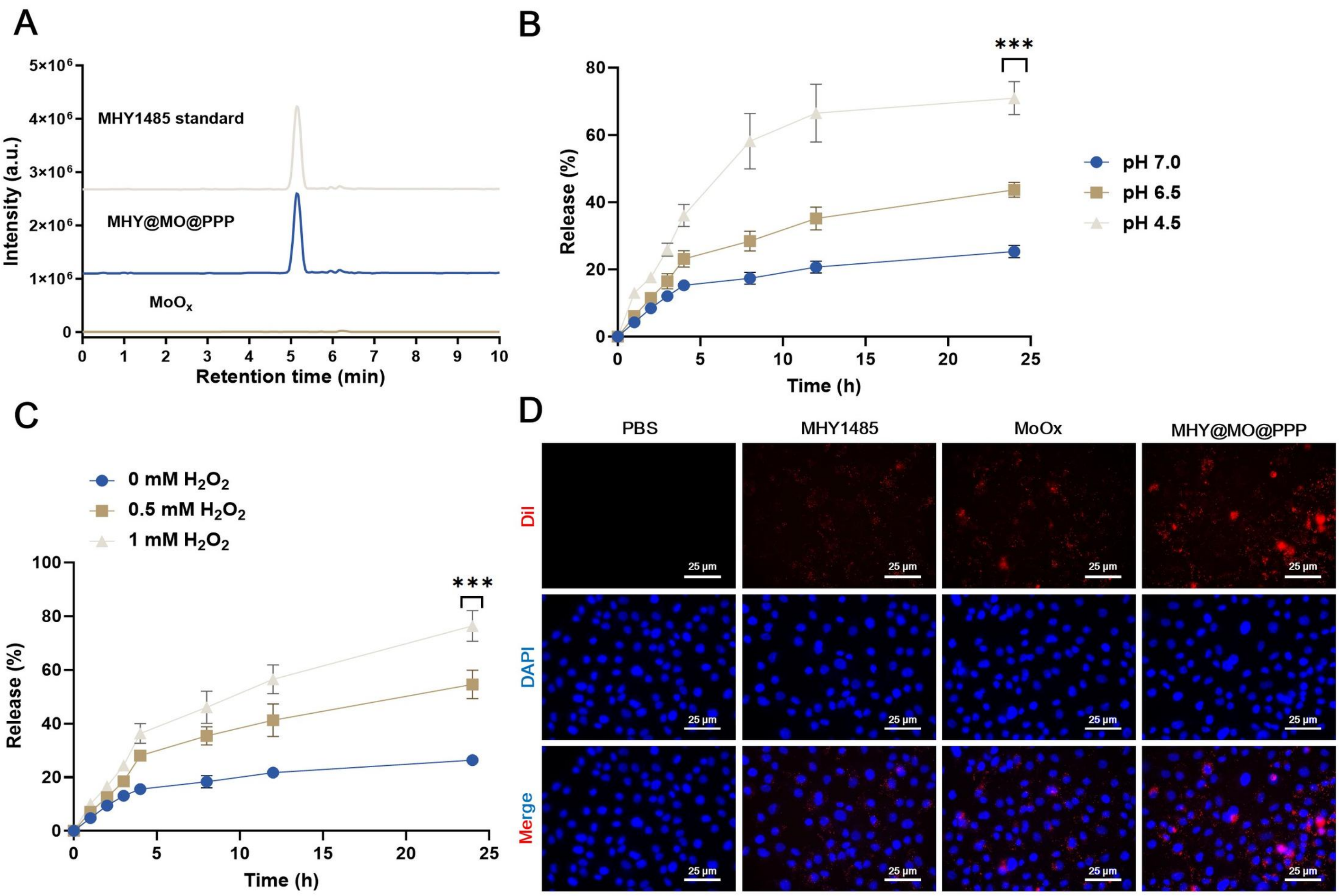

Supplementary Figure S3

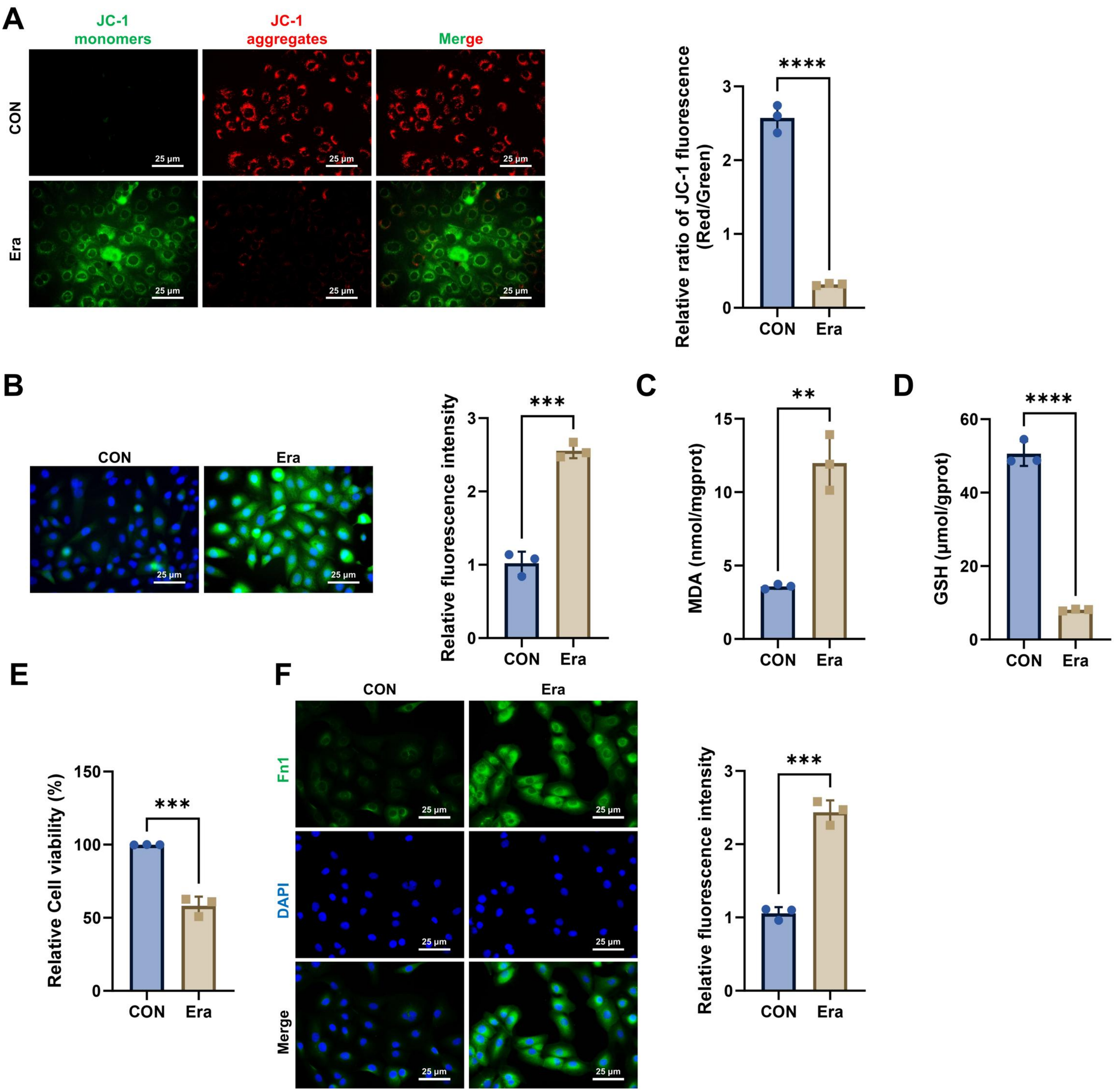

Supplementary Figure S4

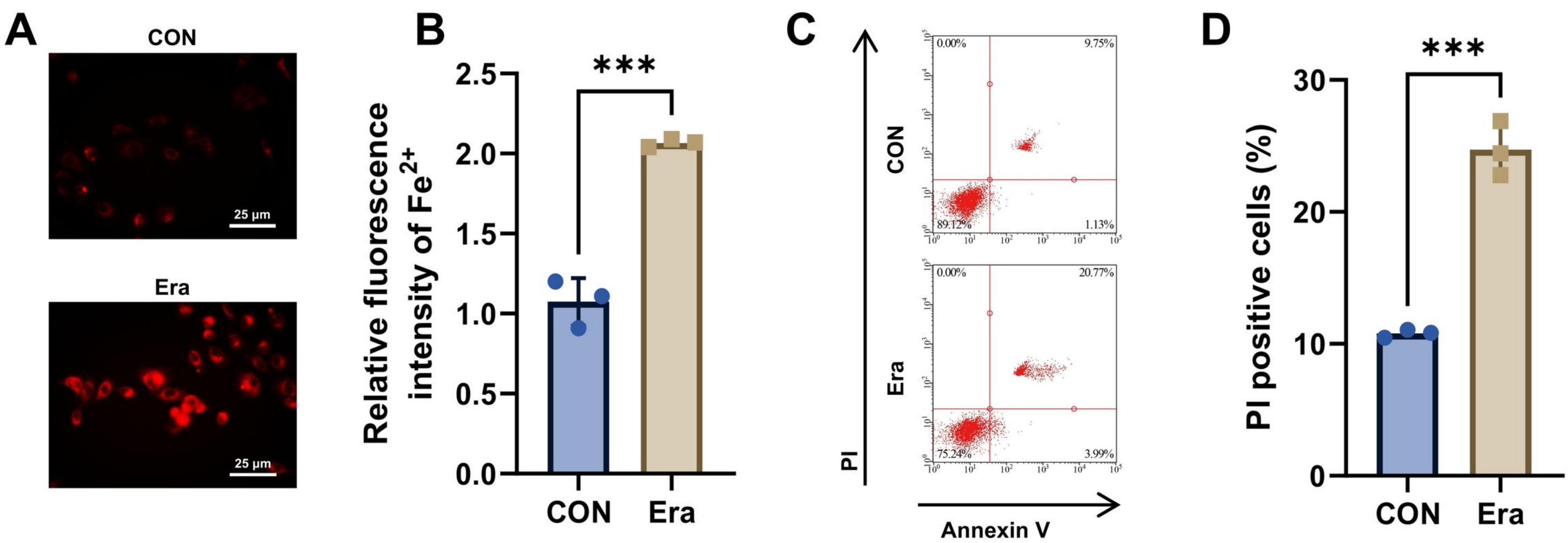

Supplementary Figure S5

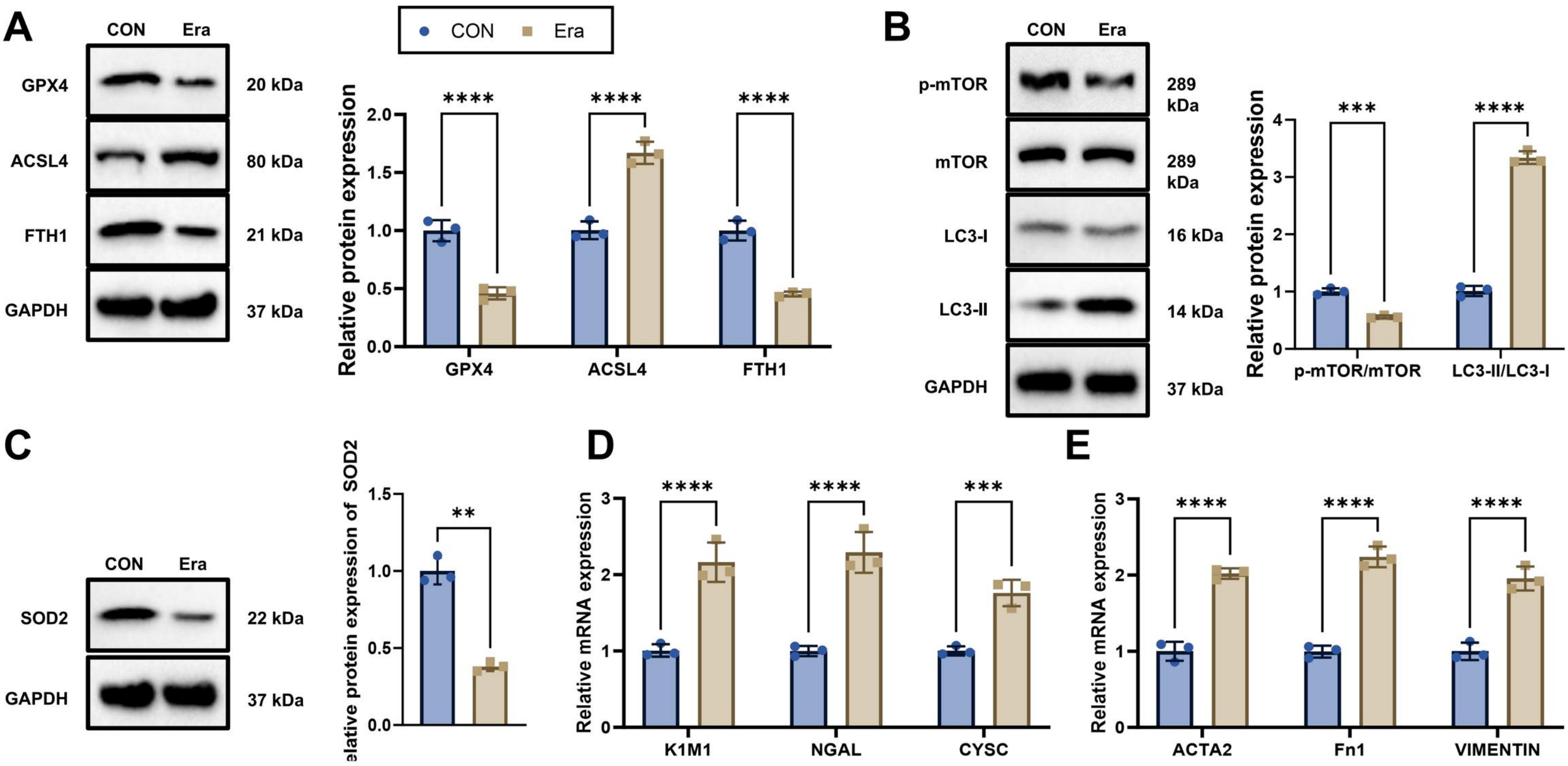

Supplementary Figure S6

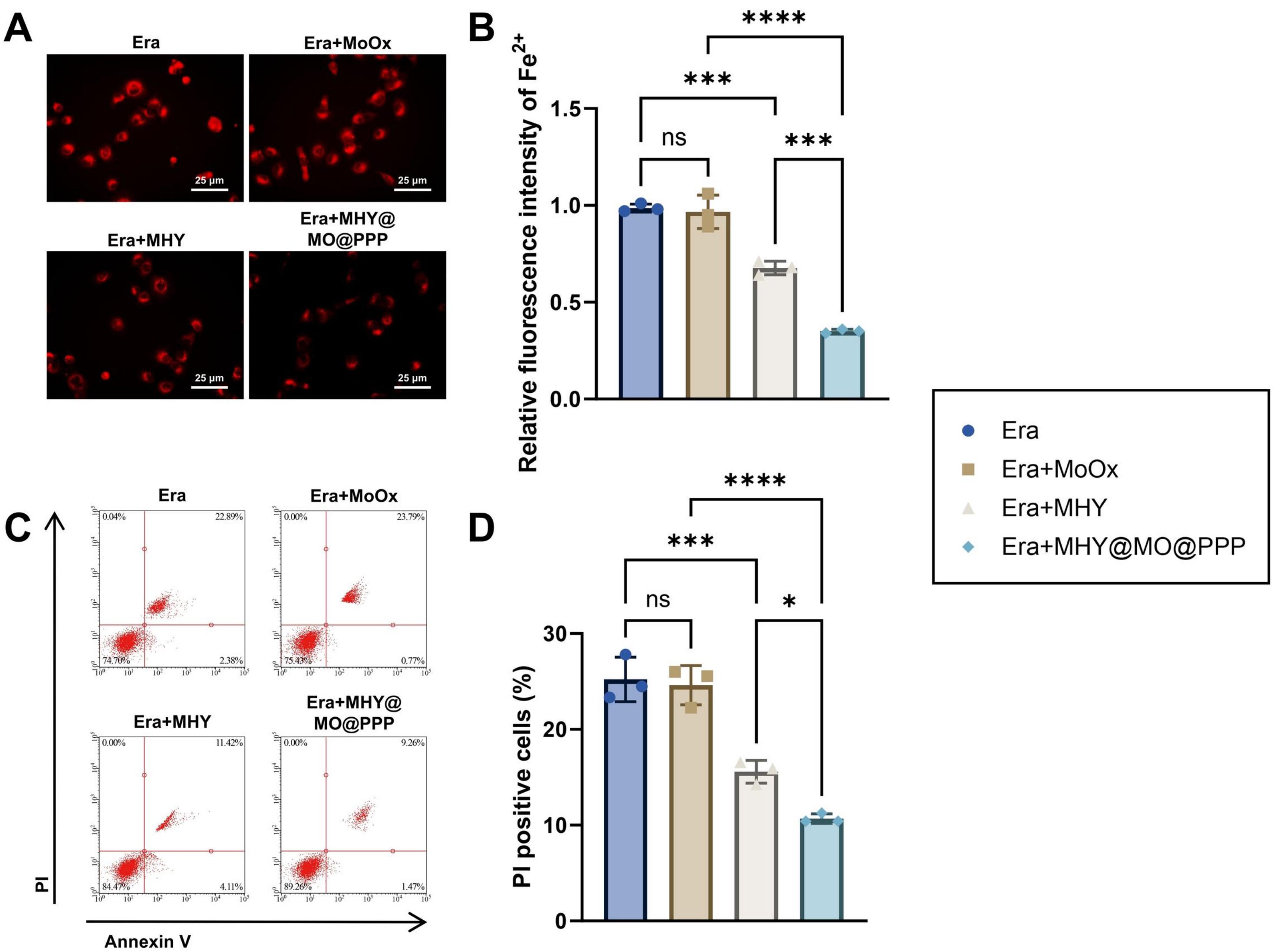

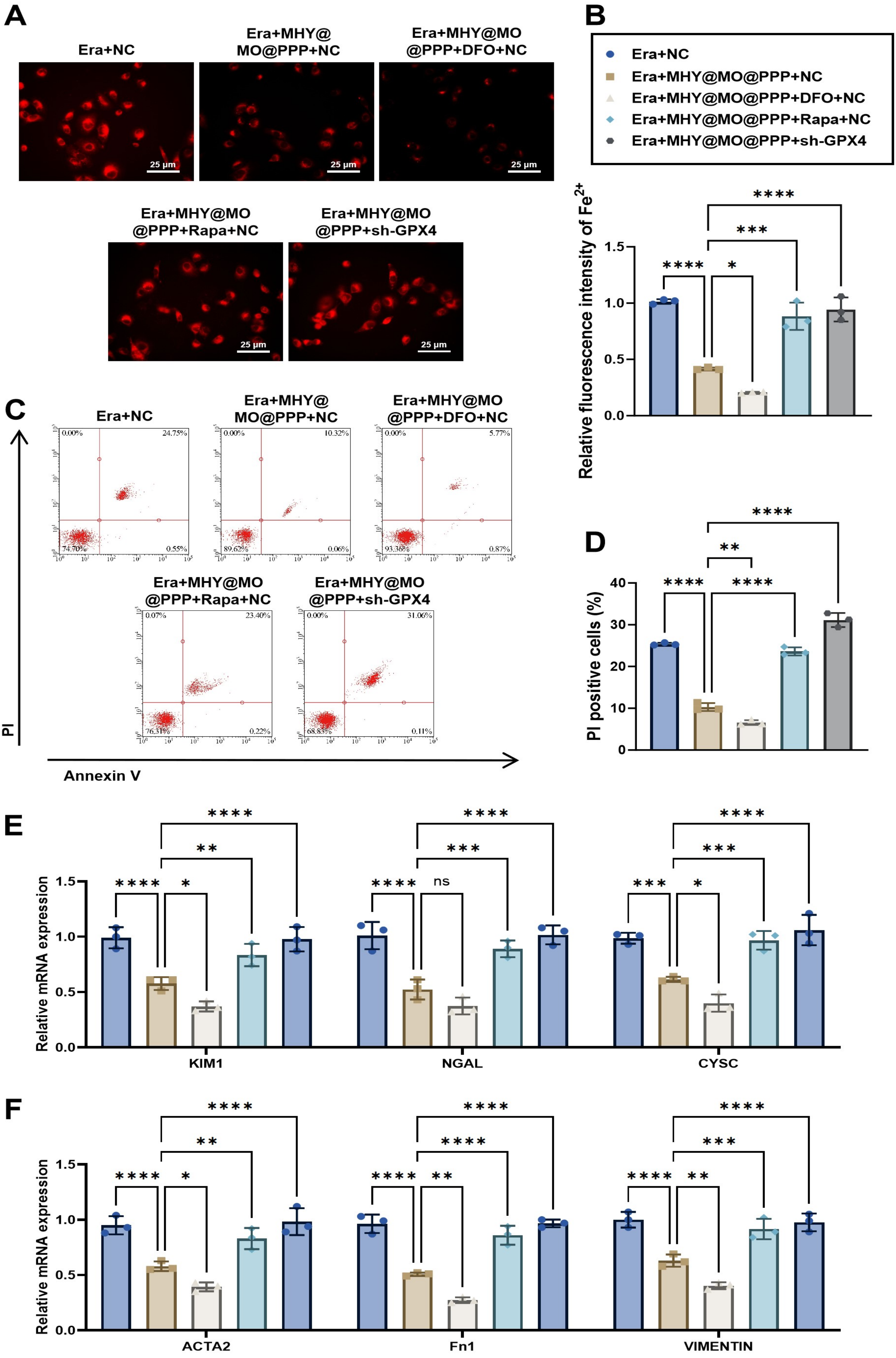
A
Era+NC
Era+MHY@MO@PPP+NC
Era+MHY@MO@PPP+DFO+NC
Era+MHY@MO@PPP+Rapa+NC
Era+MHY@MO@PPP+sh-GPX4
25 μm
B
Era+NC
Era+MHY@MO@PPP+NC
Era+MHY@MO@PPP+DFO+NC
Era+MHY@MO@PPP+Rapa+NC
Era+MHY@MO@PPP+sh-GPX4
Relative fluorescence intensity of Fe2+
C
PI
Annexin V
D
PI positive cells (%)
E
Relative mRNA expression
KIM1
NGAL
CYSC
ns
F
Relative mRNA expression
ACTA2
Fn1
VIMENTIN
