# Supplementary material for: MHY@MO@PPP inhibits ferroptosis positive feedback and delays the acute kidney injury-to-chronic kidney disease transition by remodeling the Fe2+-mTORC1-GPX4 axis
Source: J Nanobiotechnology. 2026 Aug 20;24:914. doi: 10.1186/s12951-026-04918-w (PMC13625235; doi:10.1186/s12951-026-04918-w)

Figure 3A source image 1

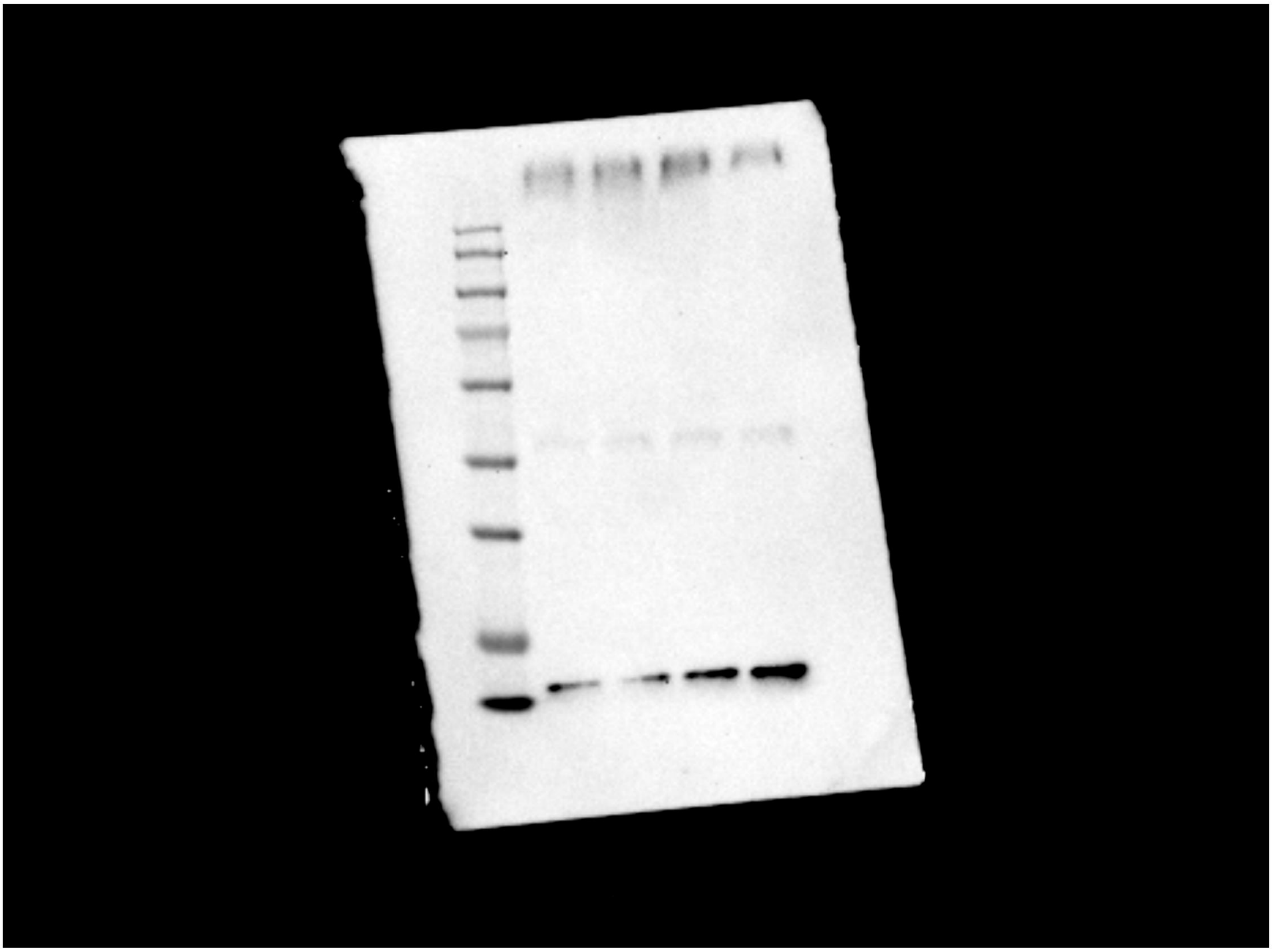

Figure 3A source image 2

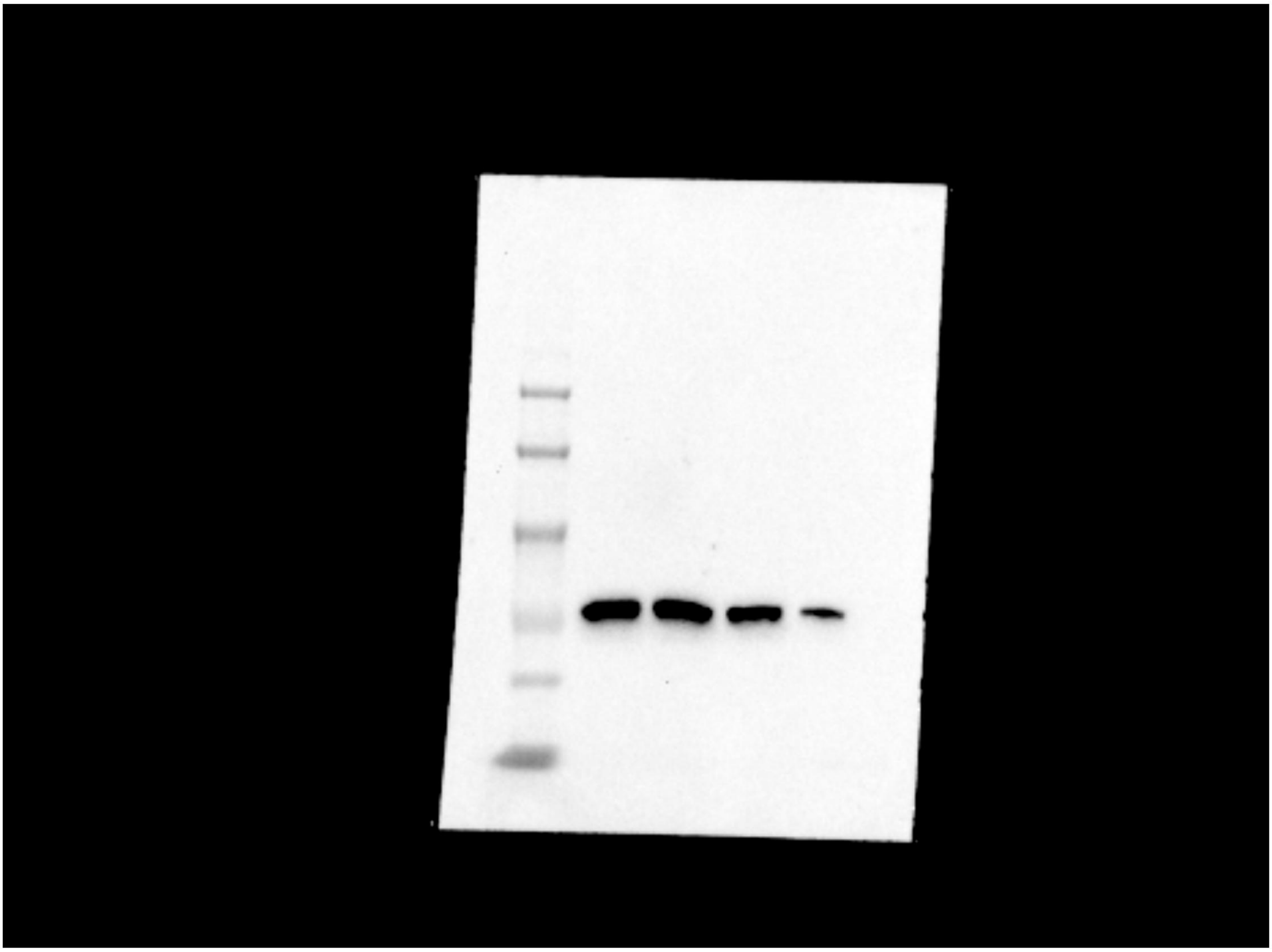

Figure 3A source image 3

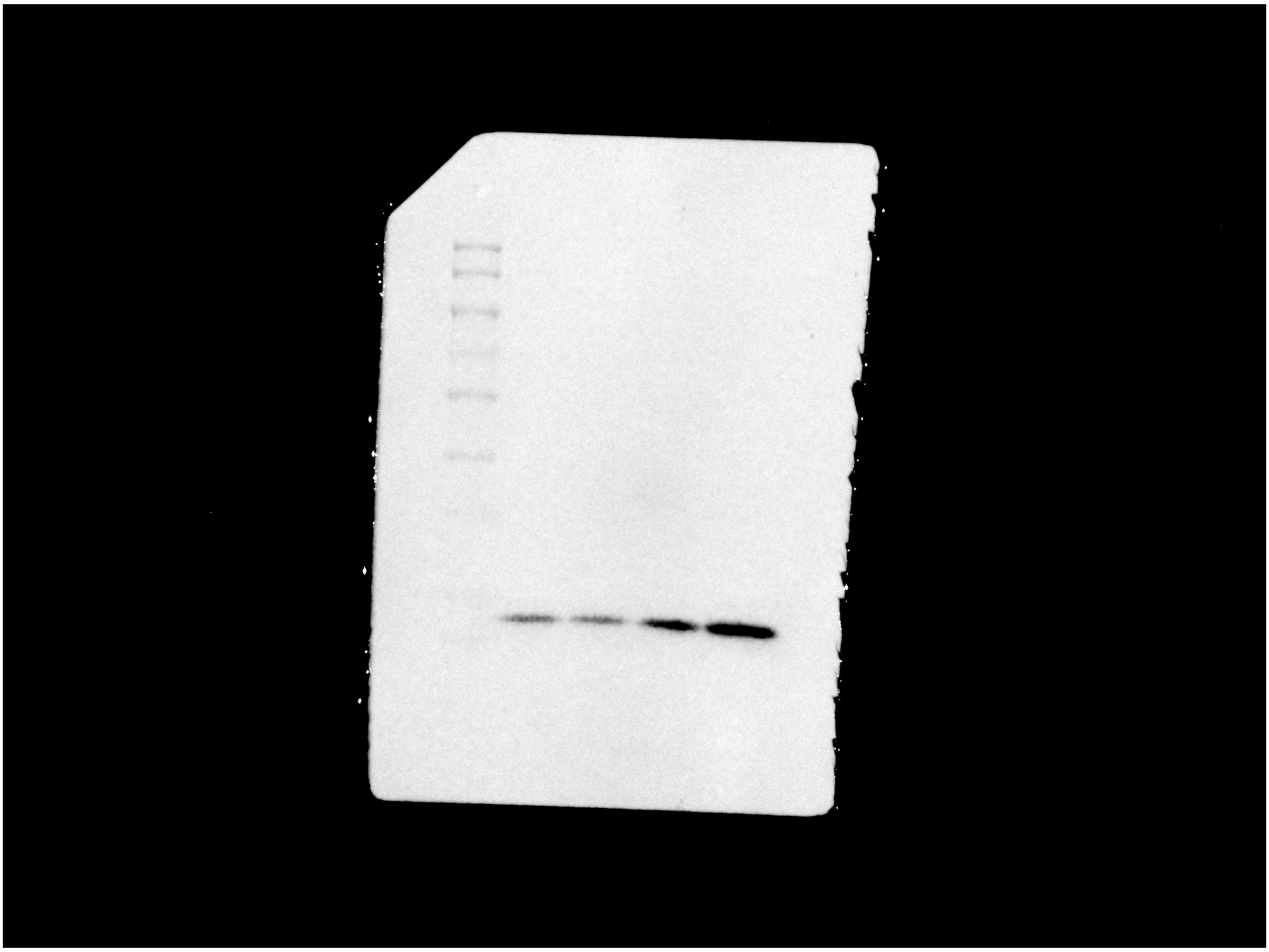

Figure 3A source image 4

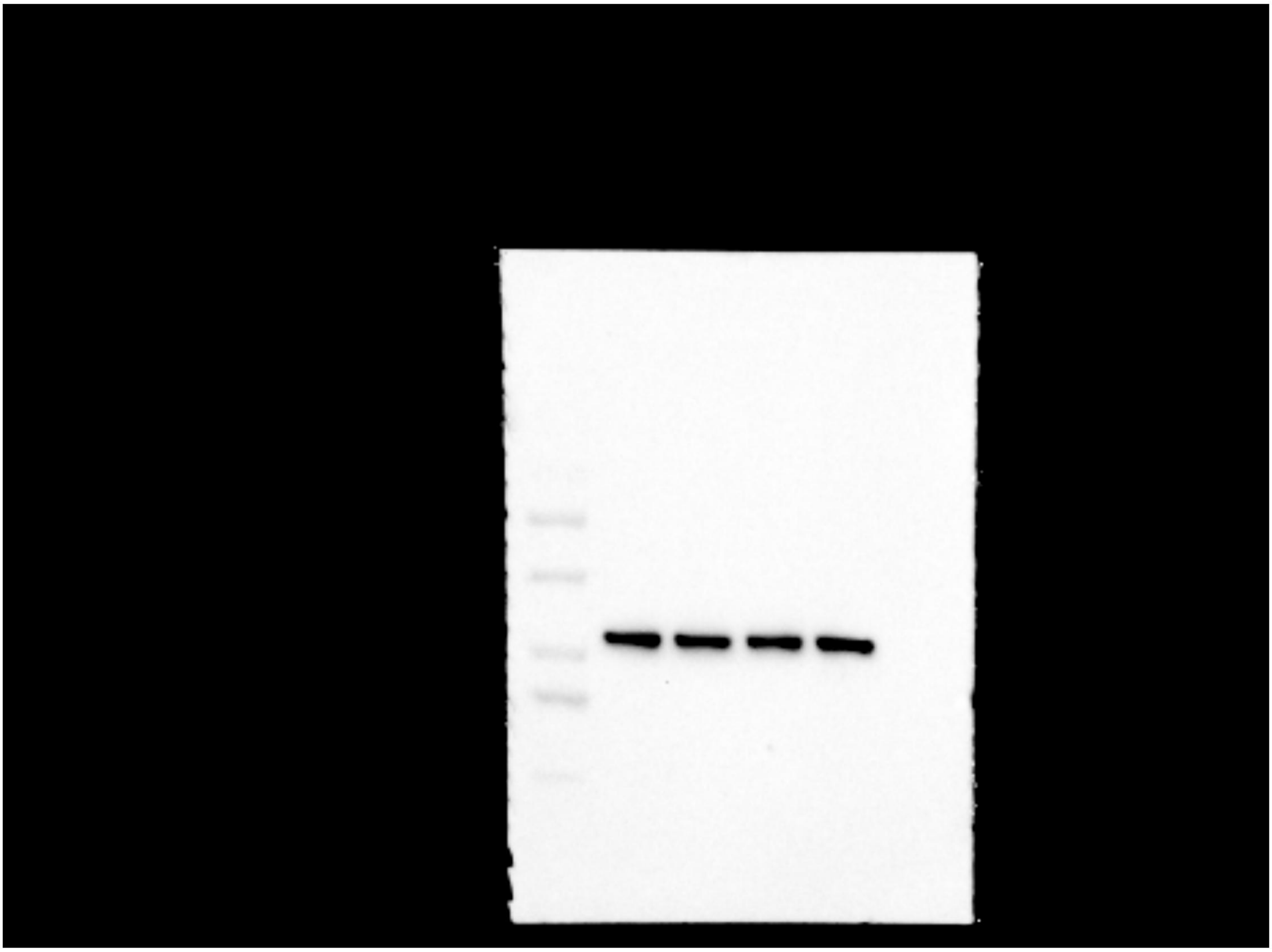

Figure 3B source image 1

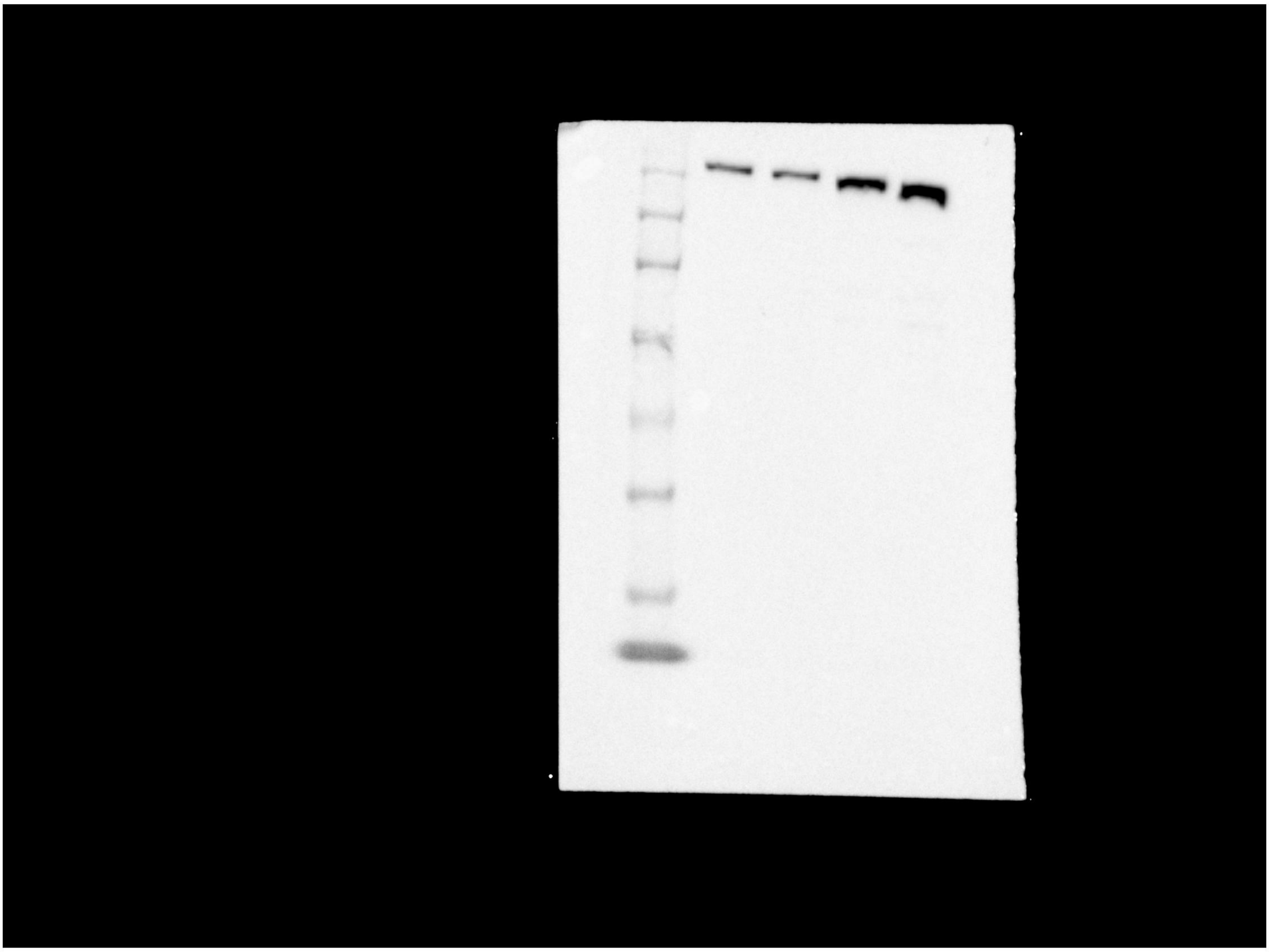

Figure 3B source image 2

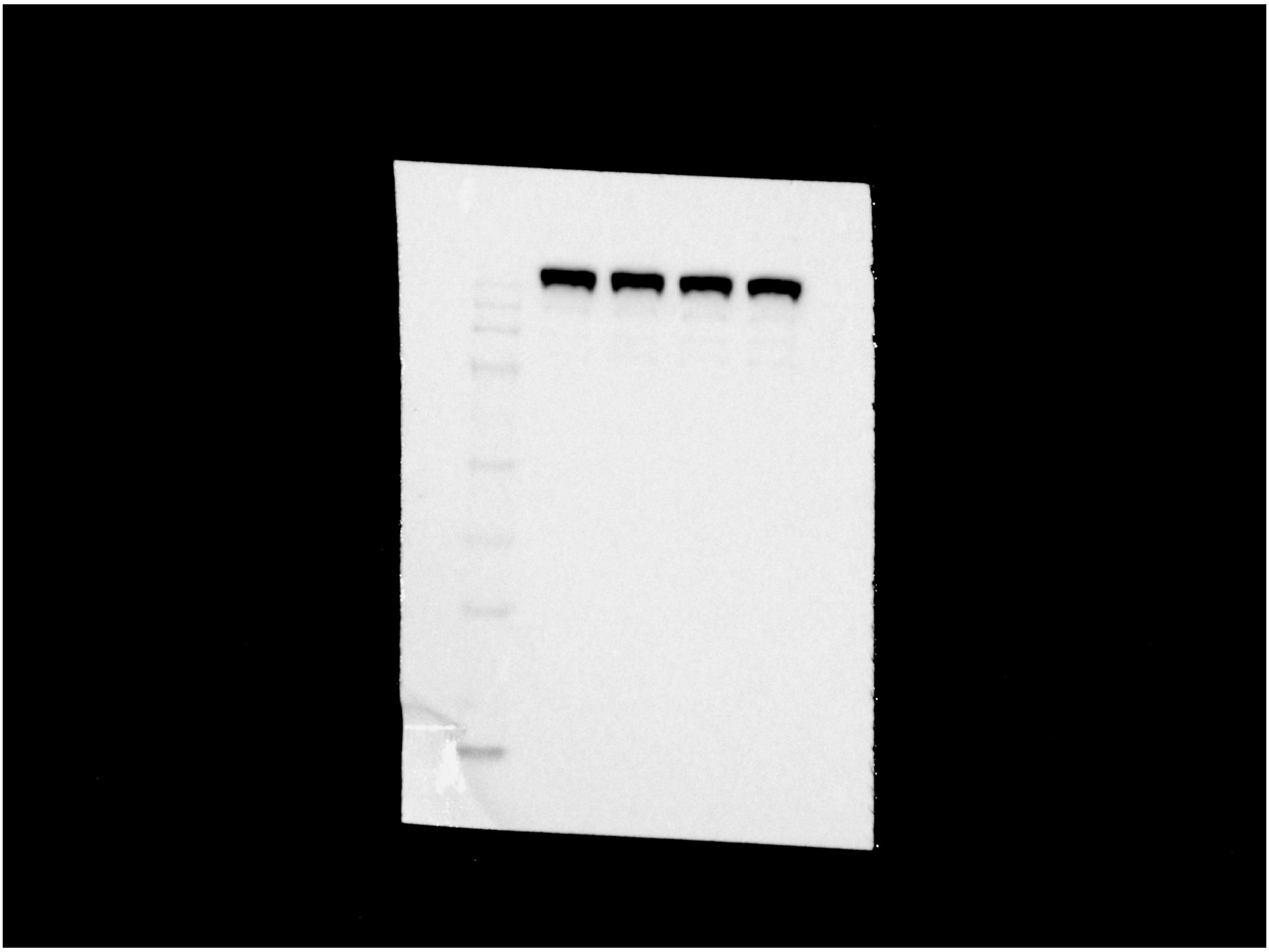

Figure 3B source image 3

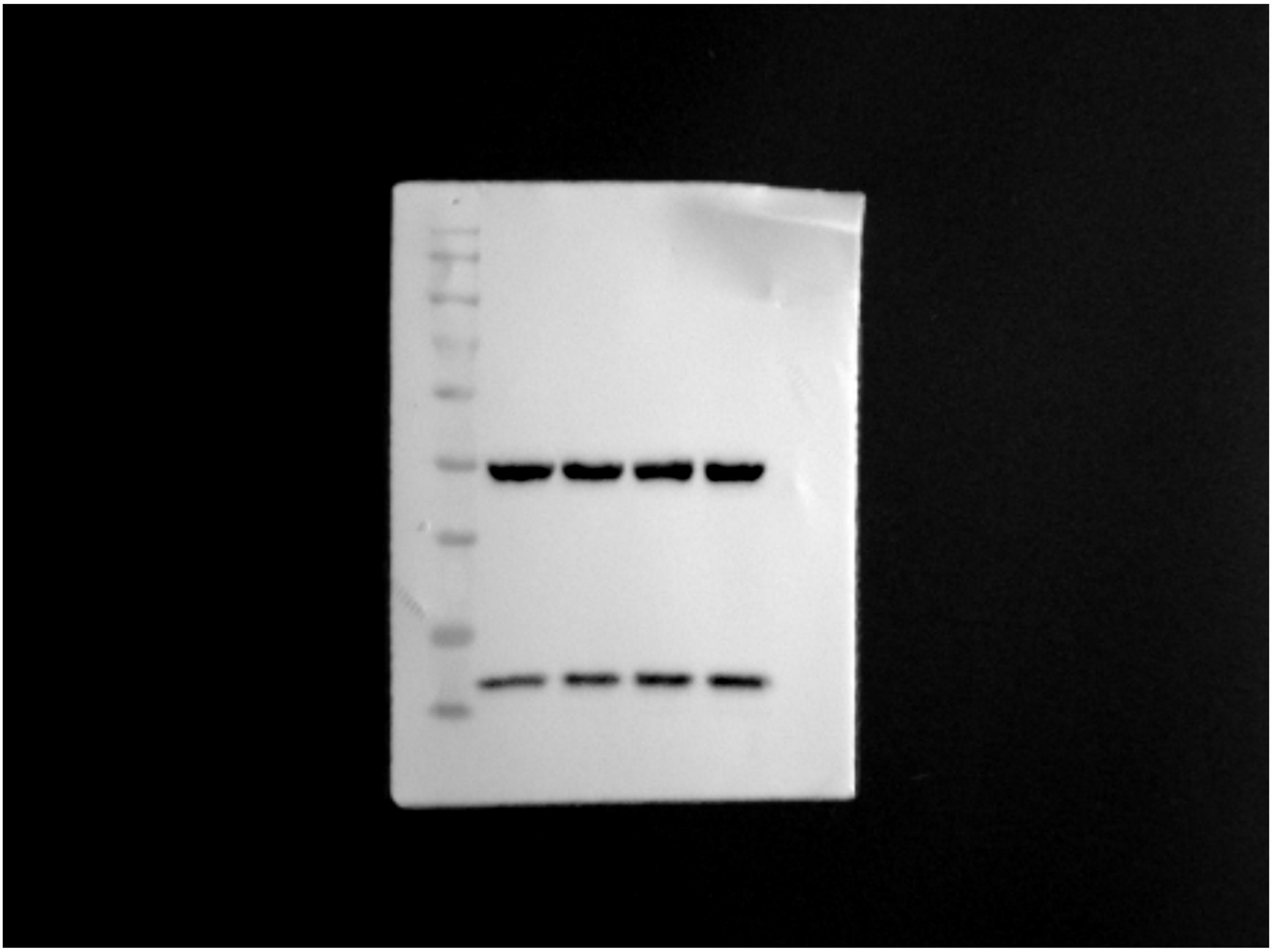

Figure 3B source image 4

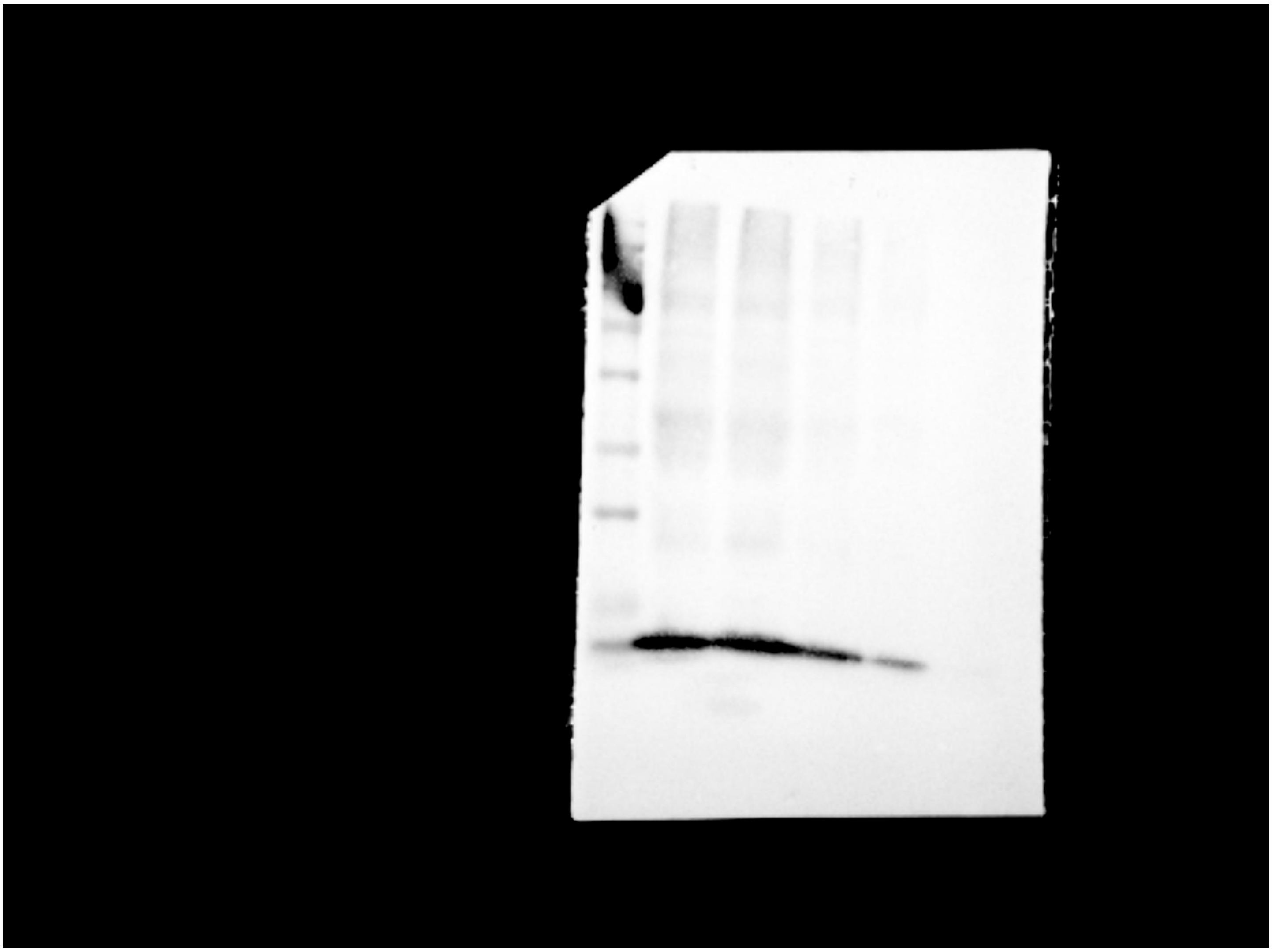

Figure 3B source image 5

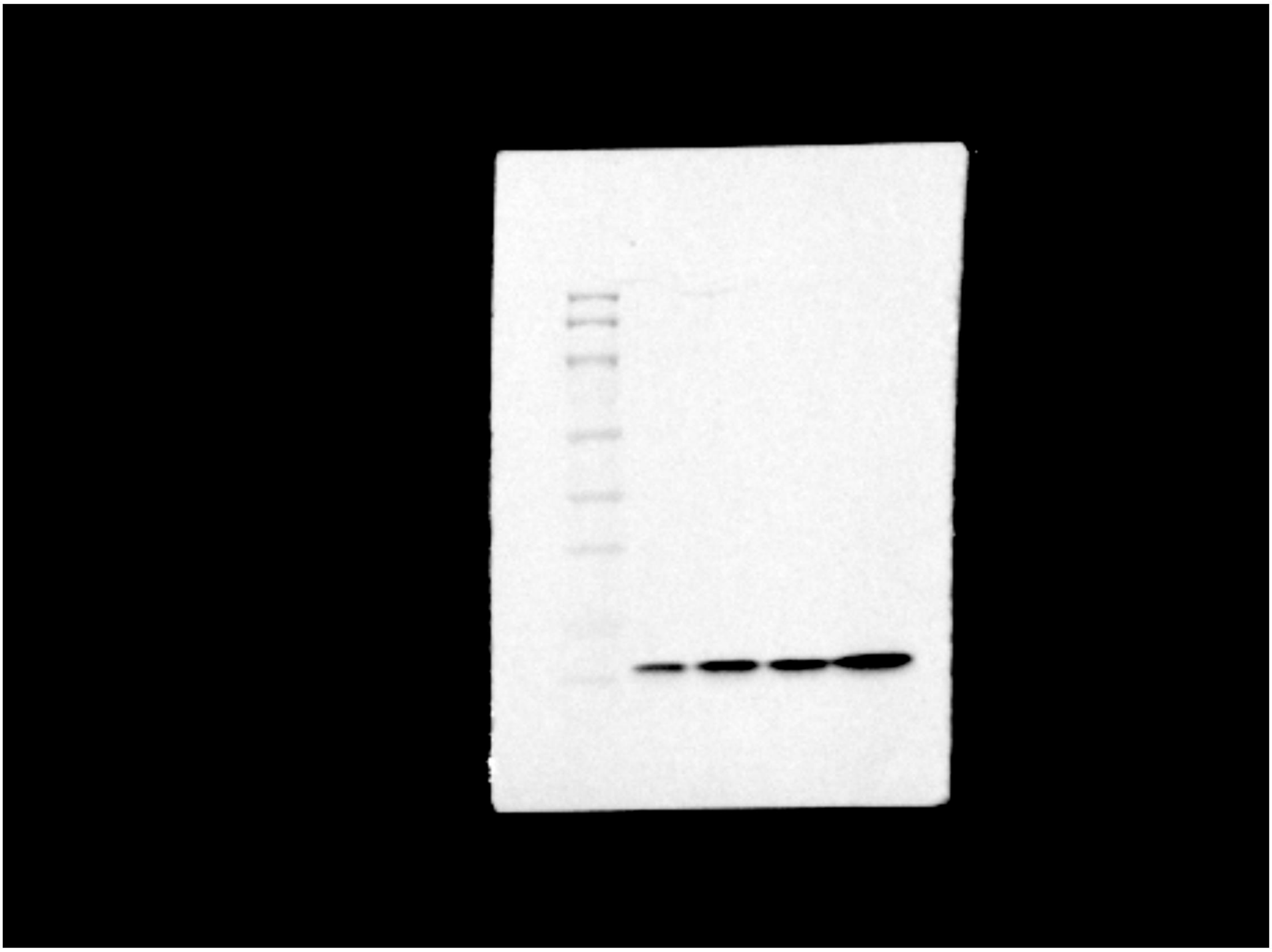

Figure 3B source image 6

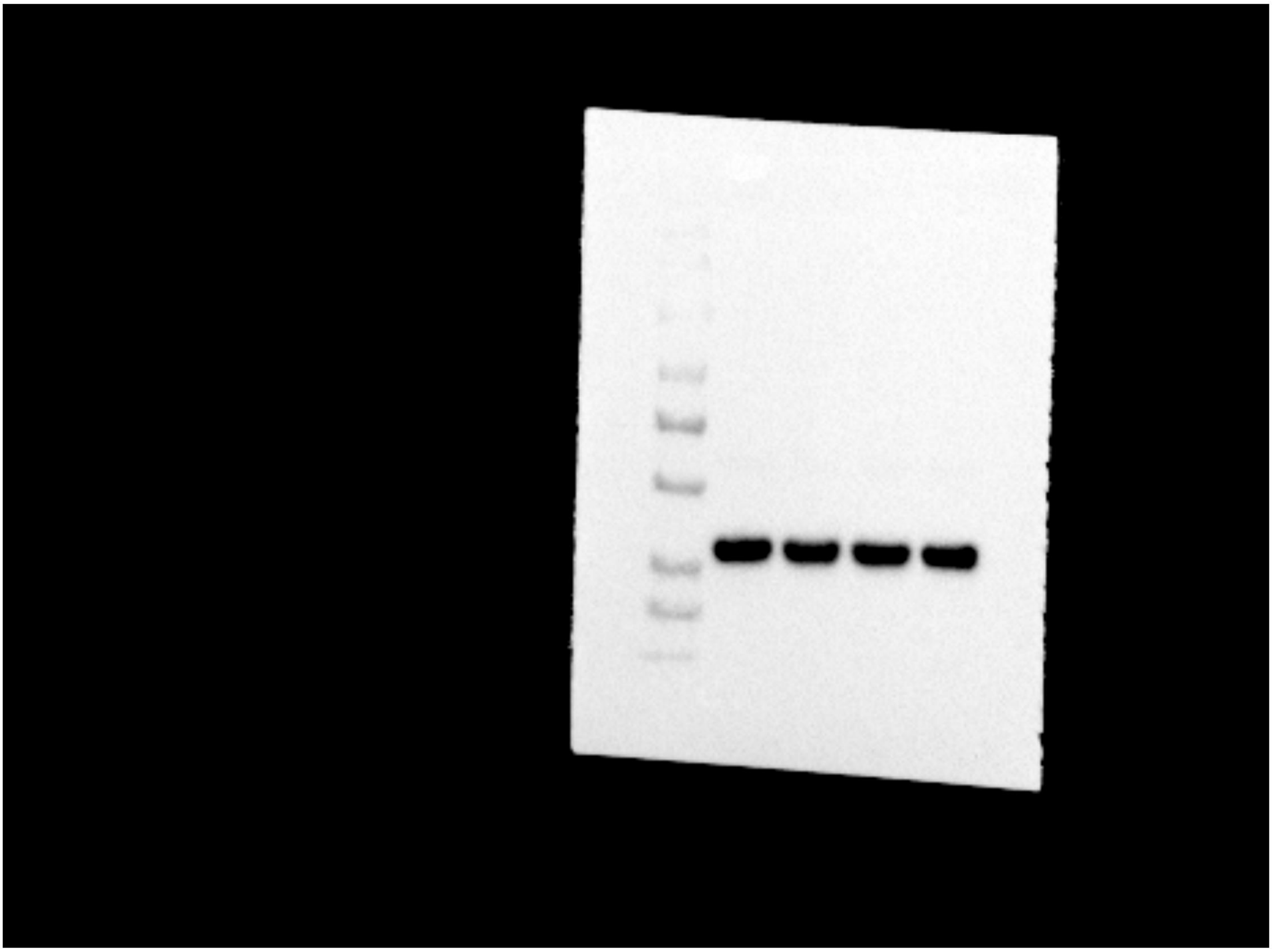

Figure 5A source image 1

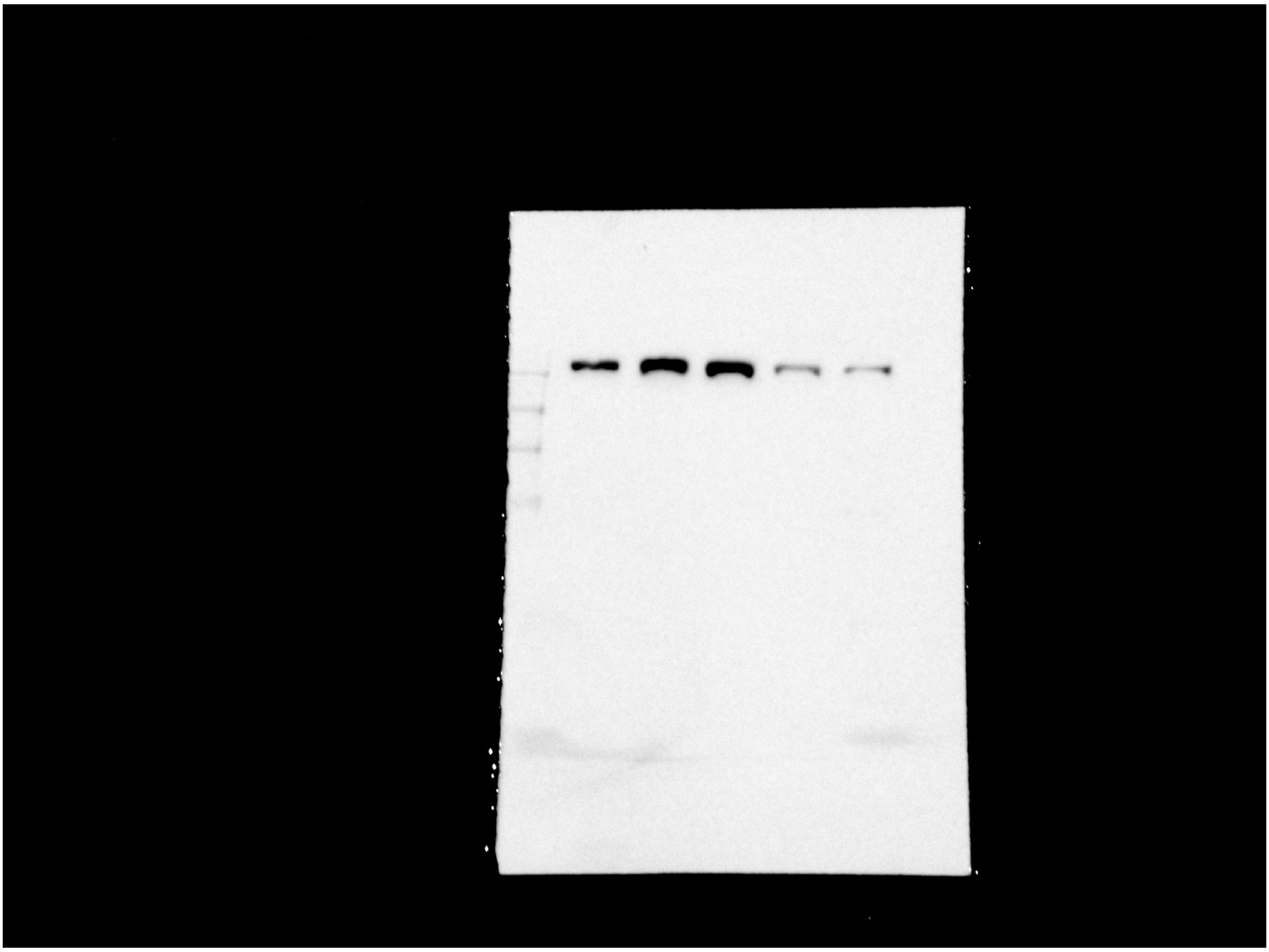

Figure 5A source image 2

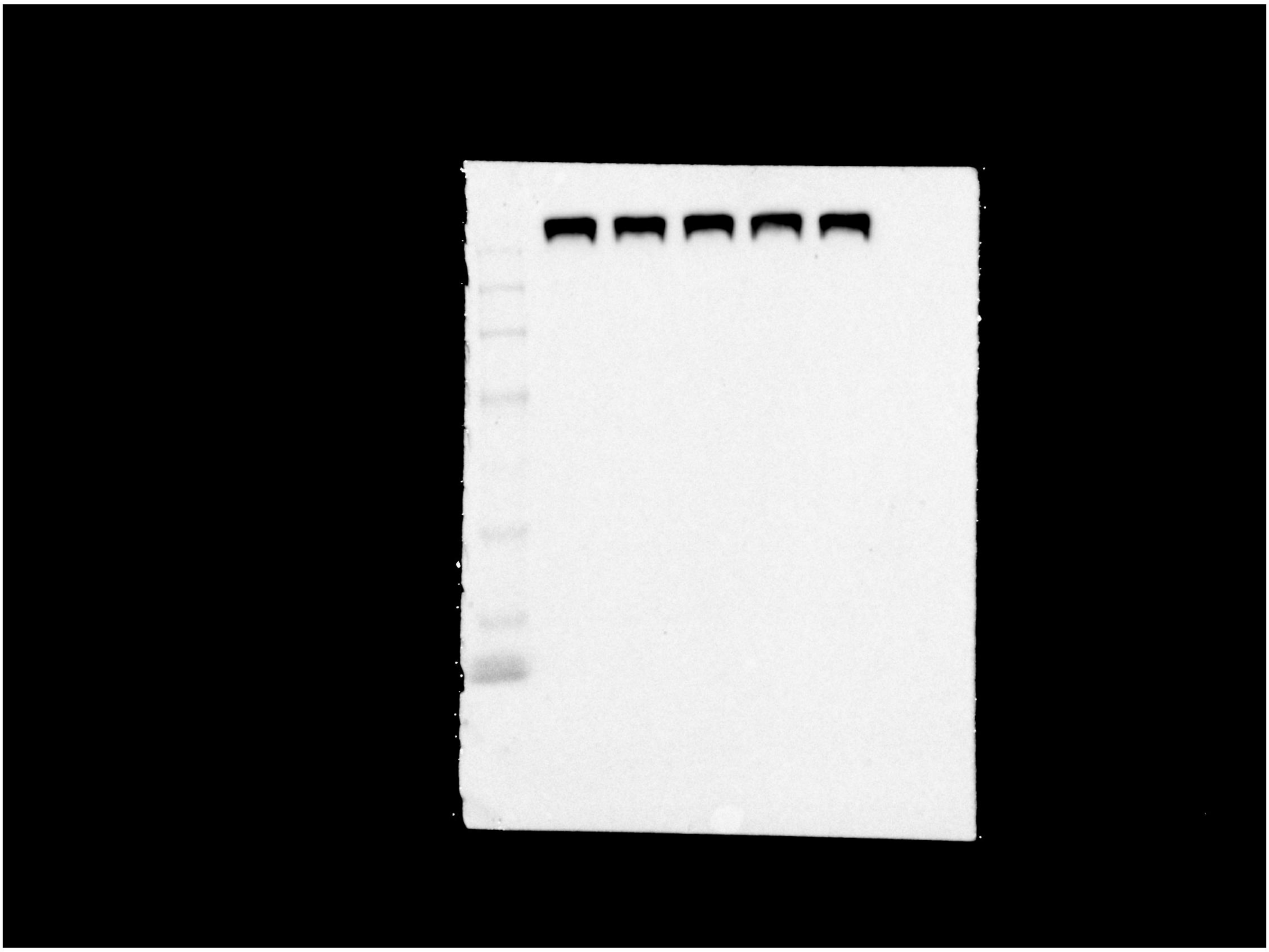

Figure 5A source image 3

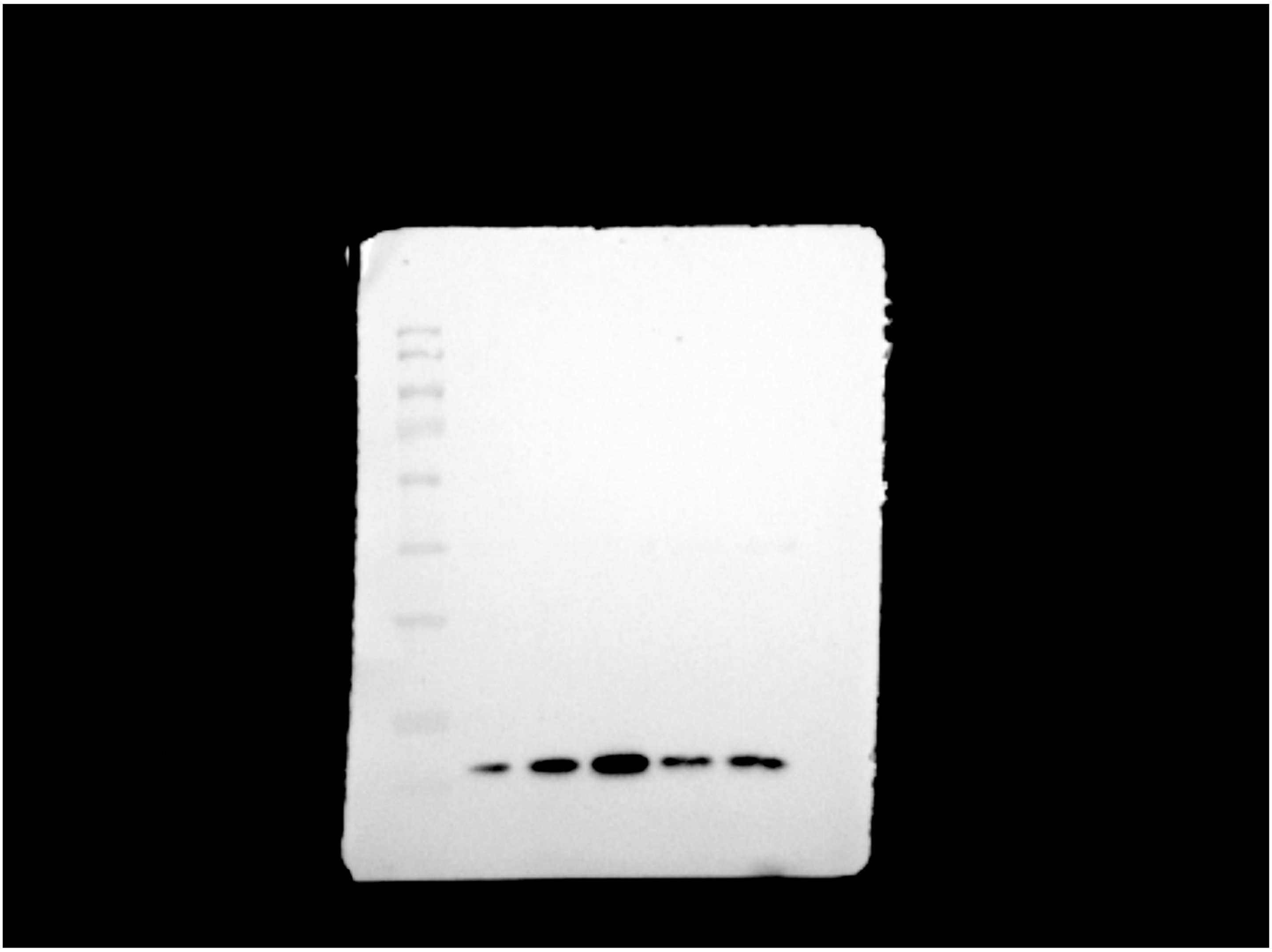

Figure 5A source image 4

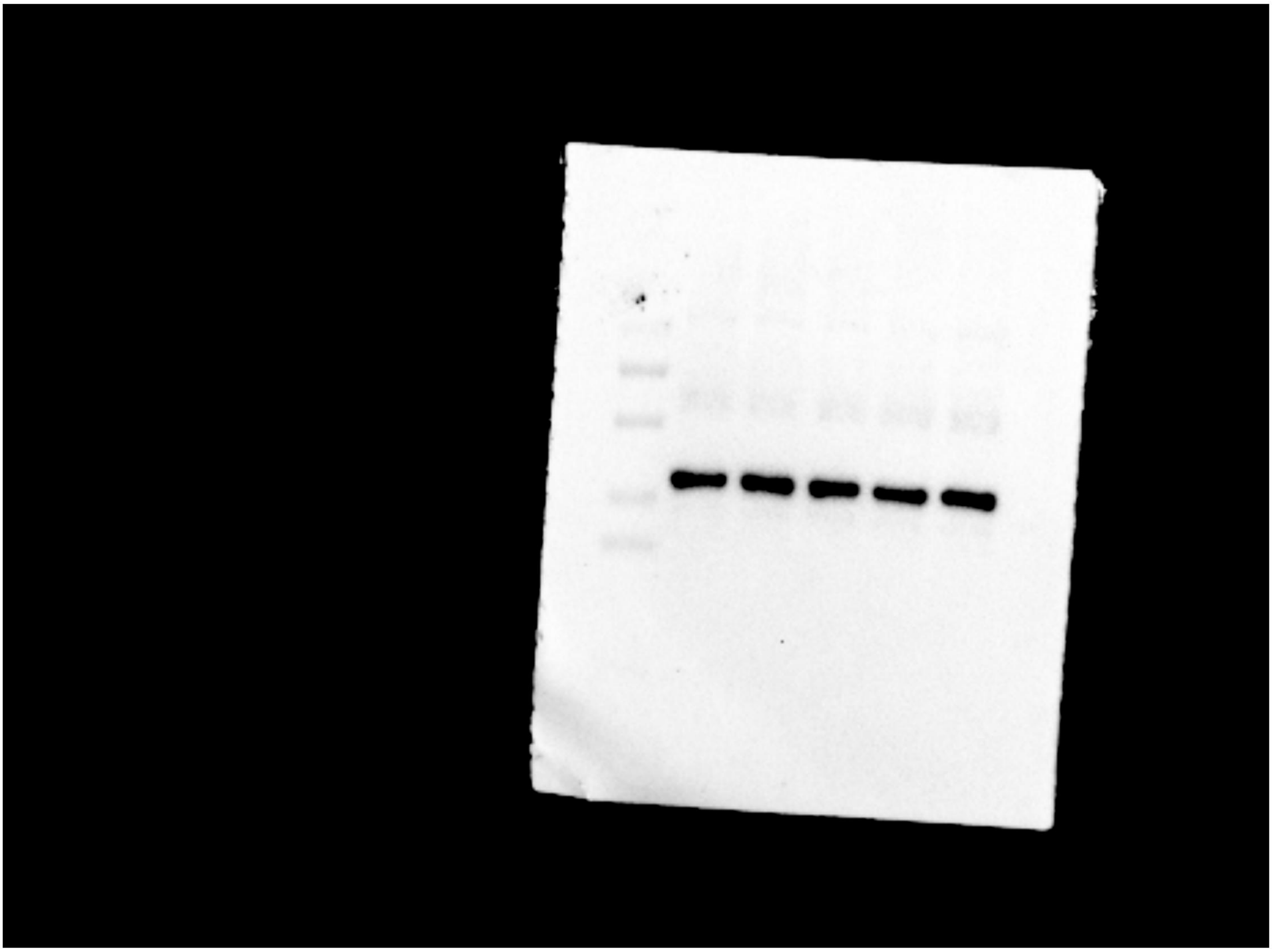

Supplementary Figure S5A source image 1

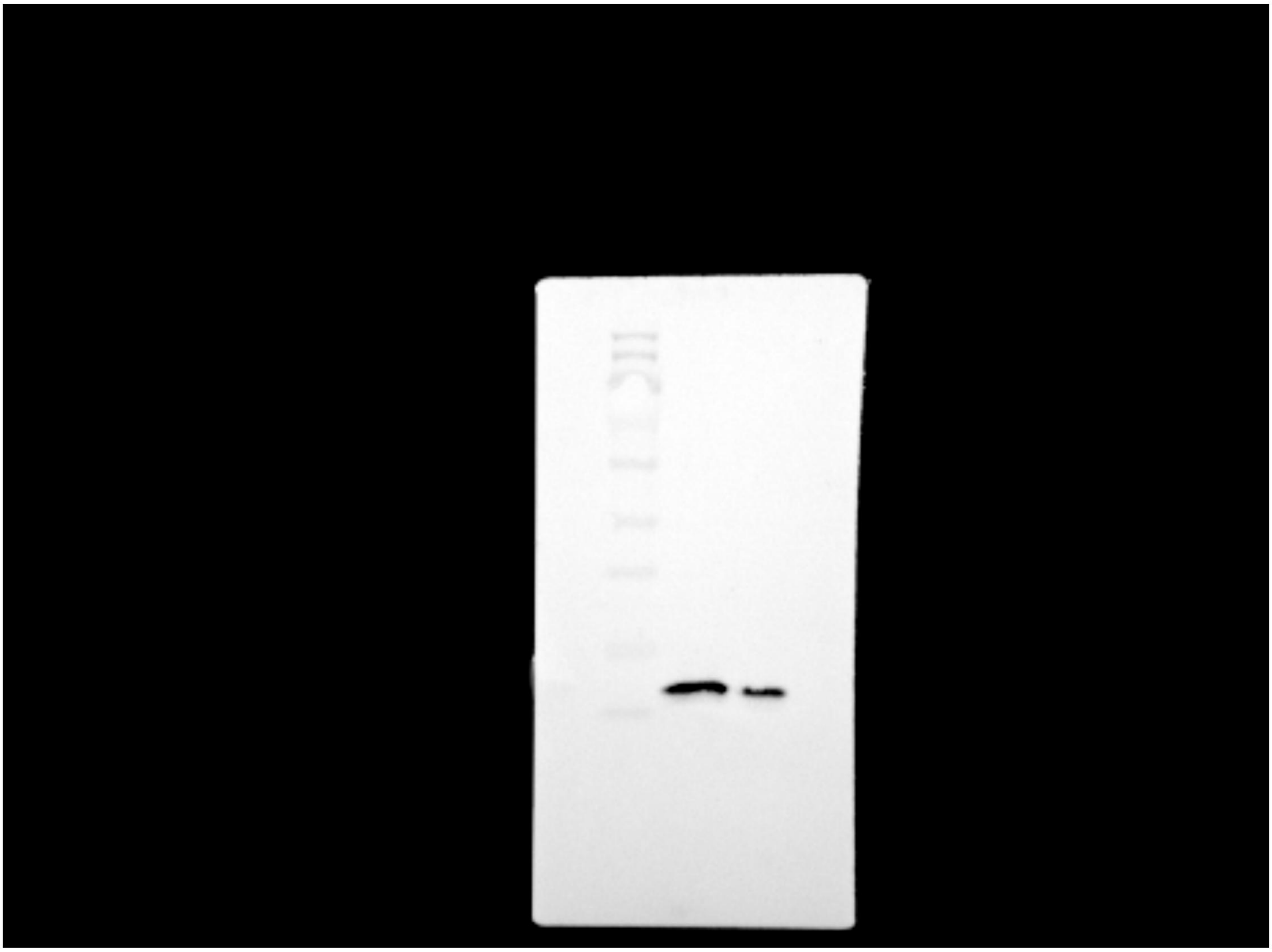

Supplementary Figure S5A source image 2

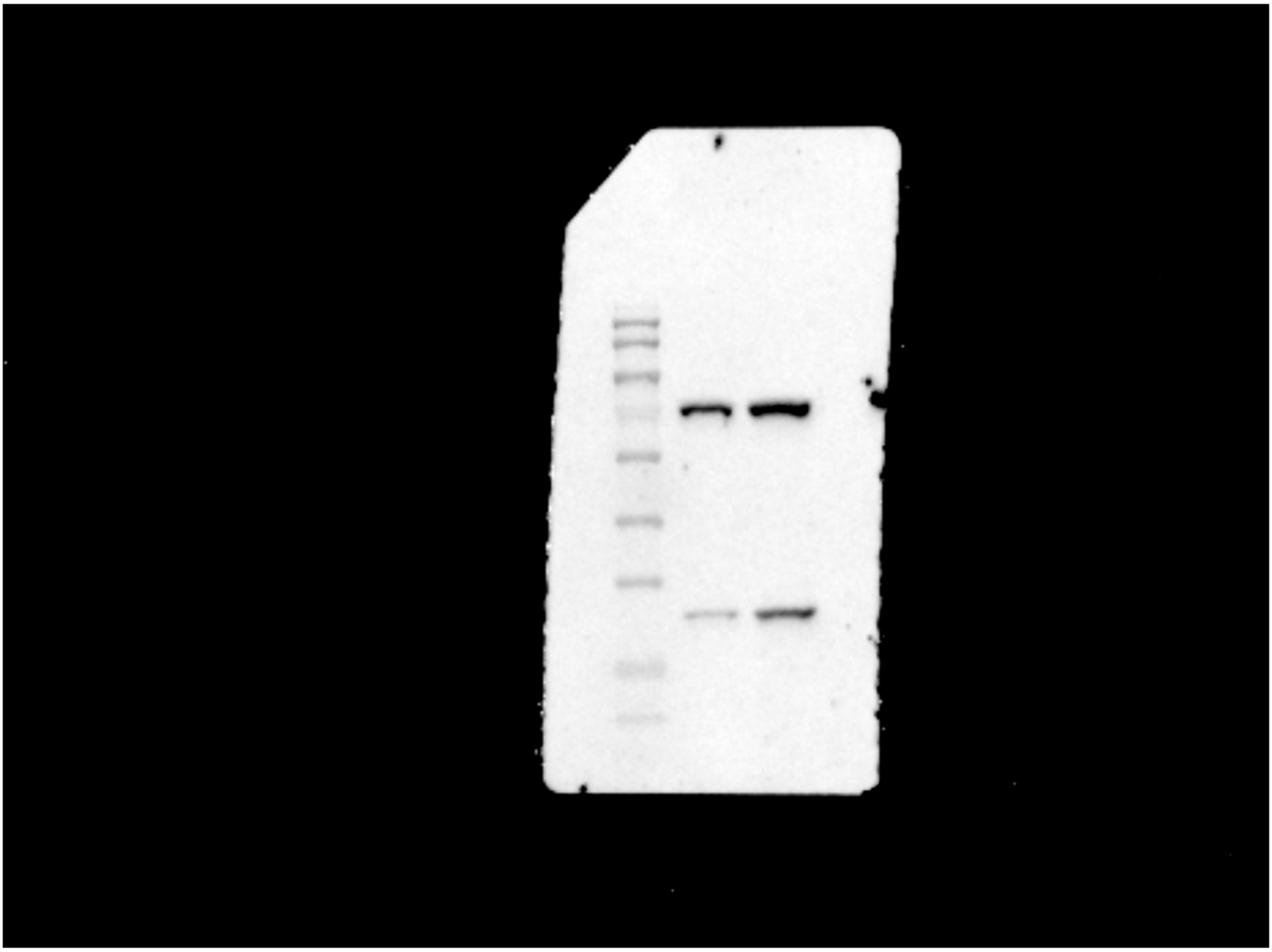

Supplementary Figure S5A source image 3

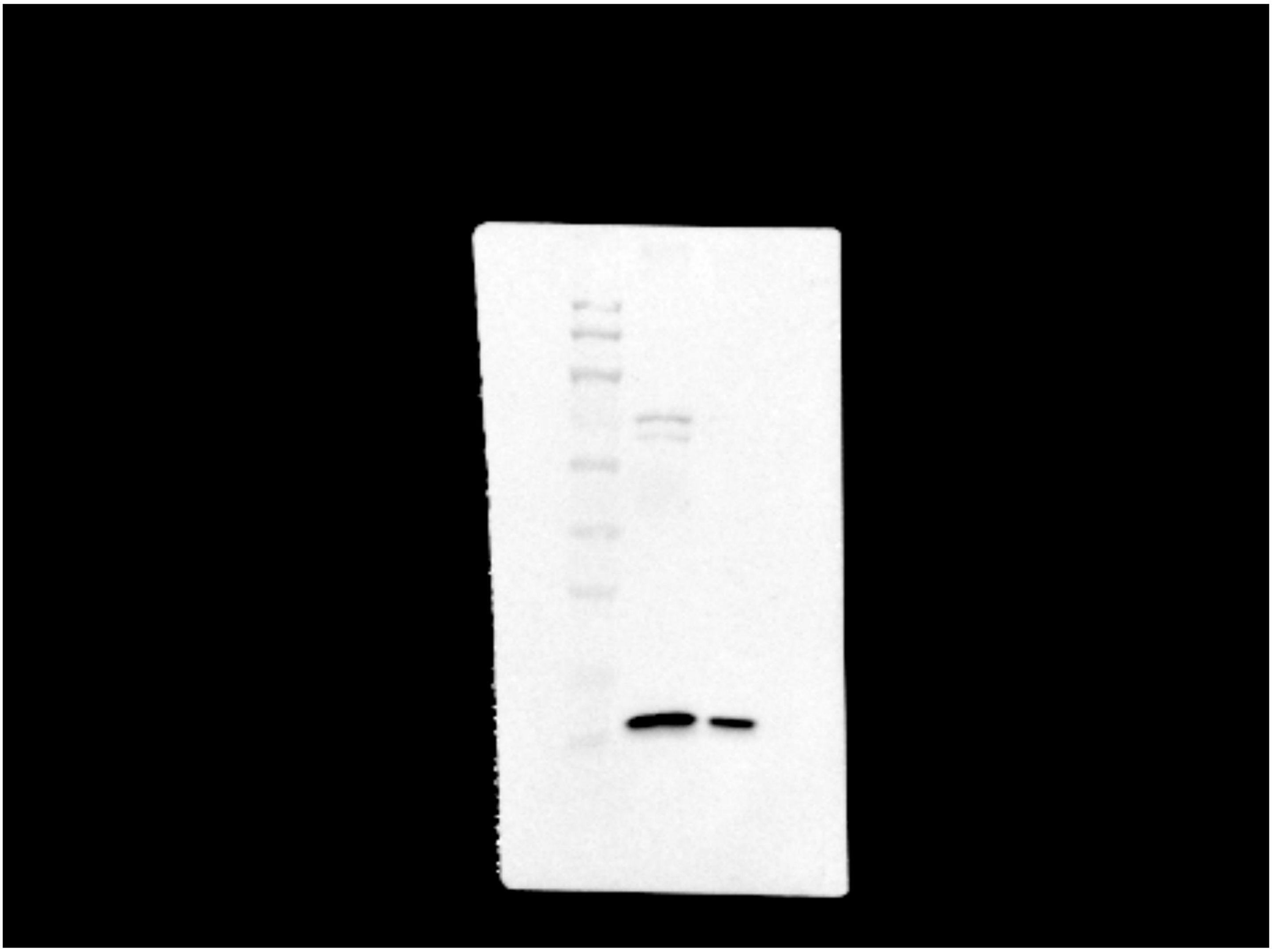

Supplementary Figure S5A source image 4

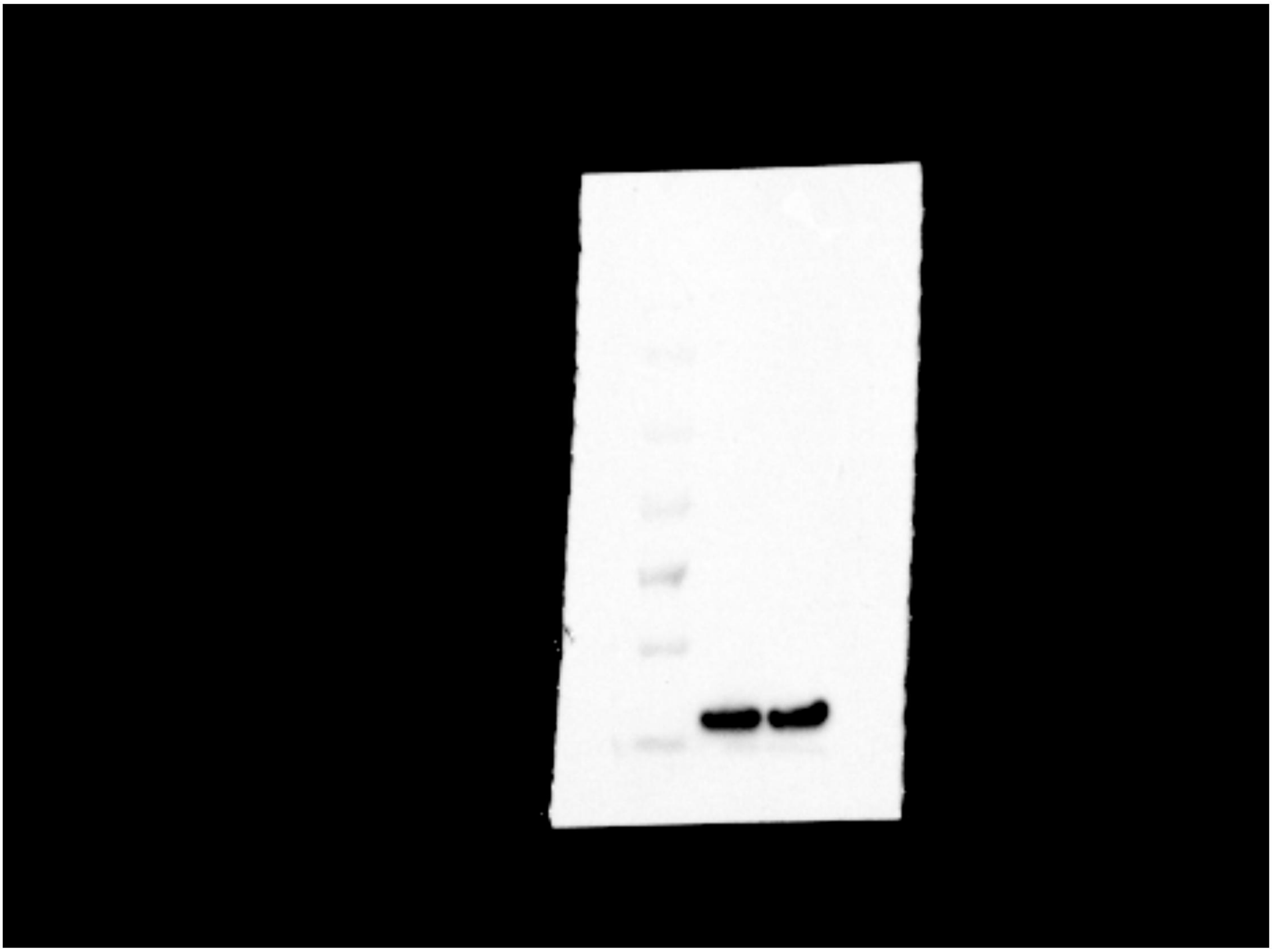

Supplementary Figure S5B source image 1

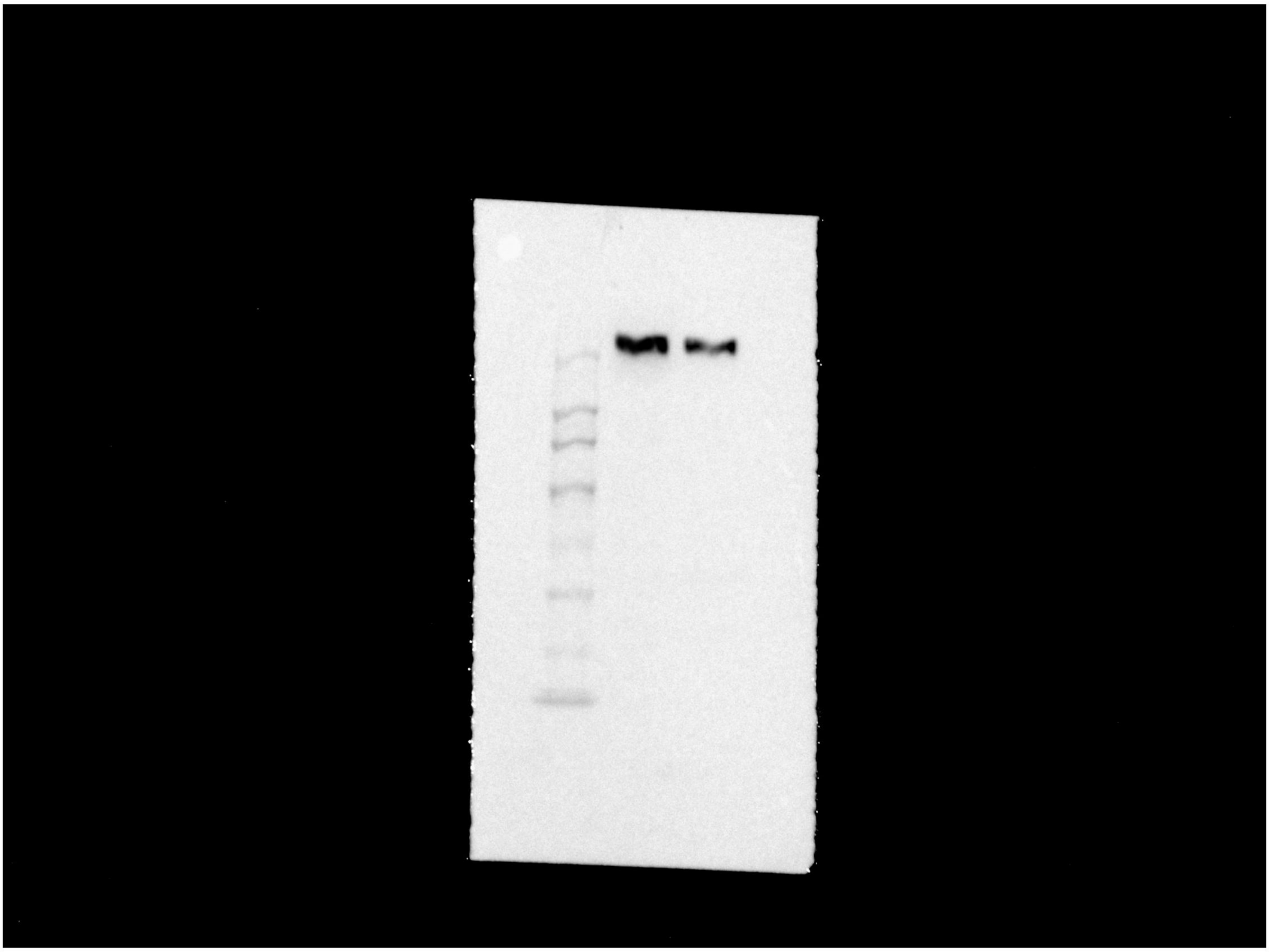

Supplementary Figure S5B source image 2

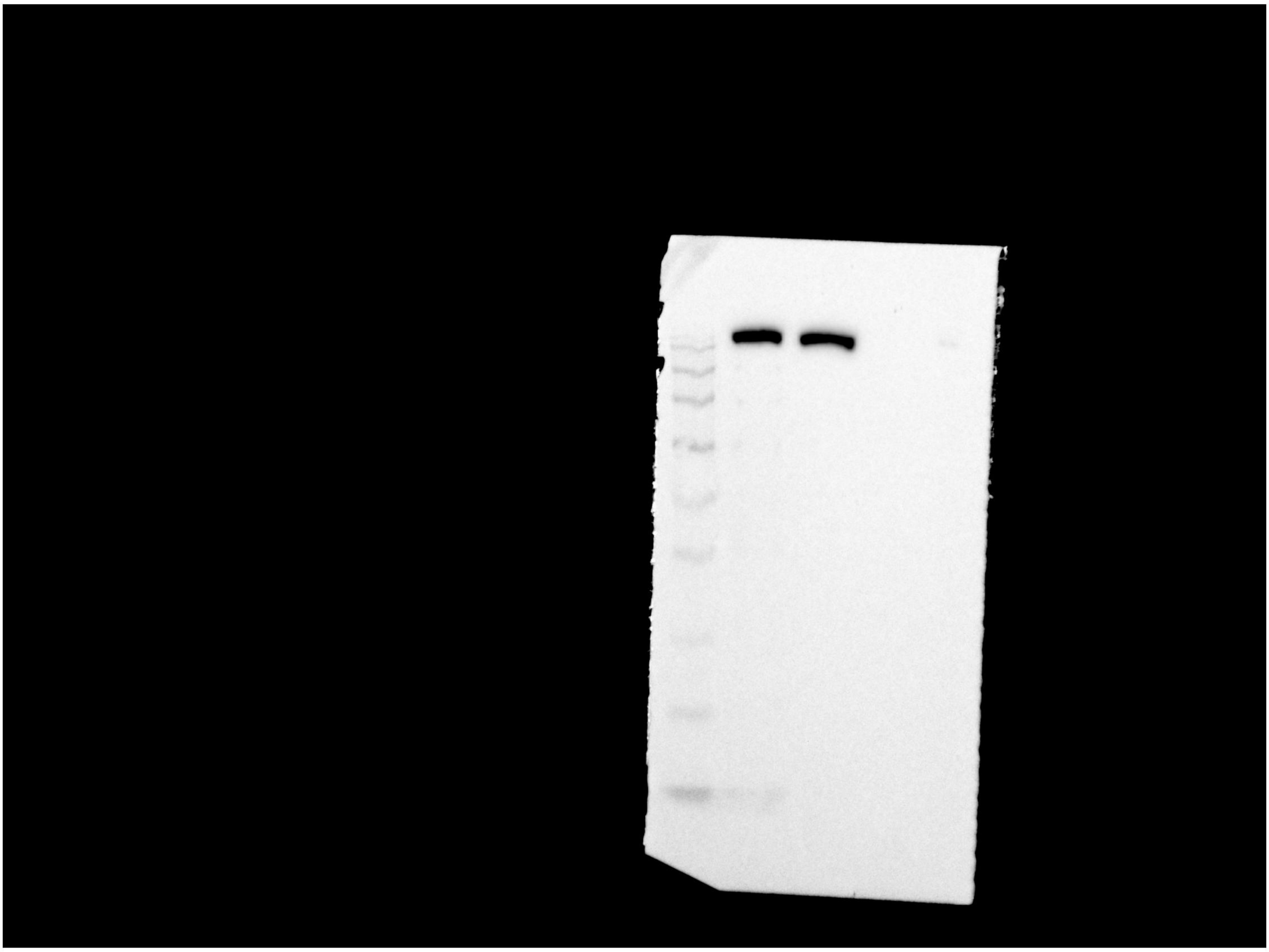

Supplementary Figure S5B source image 3

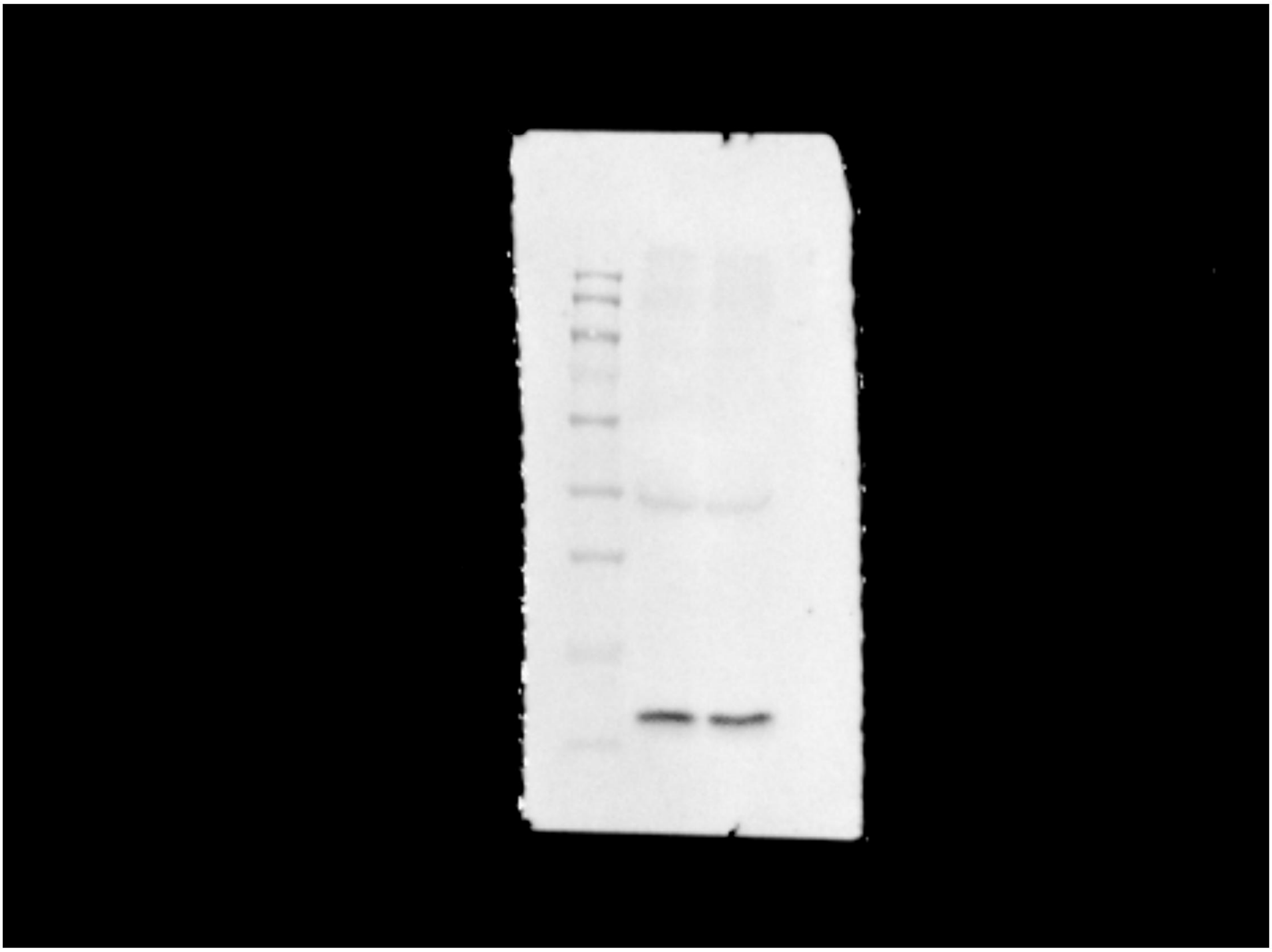

Supplementary Figure S5B source image 4

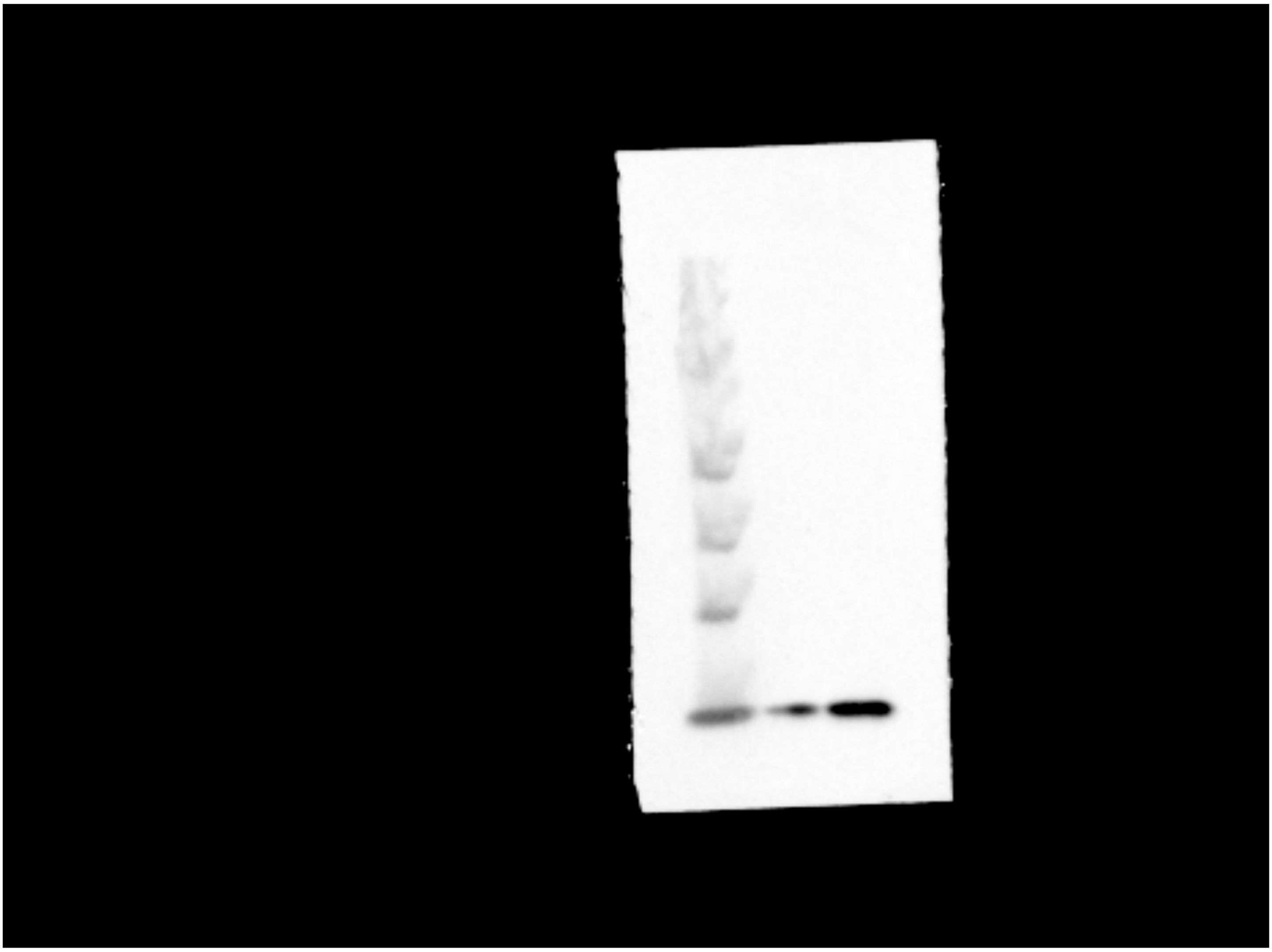

Supplementary Figure S5B source image 5

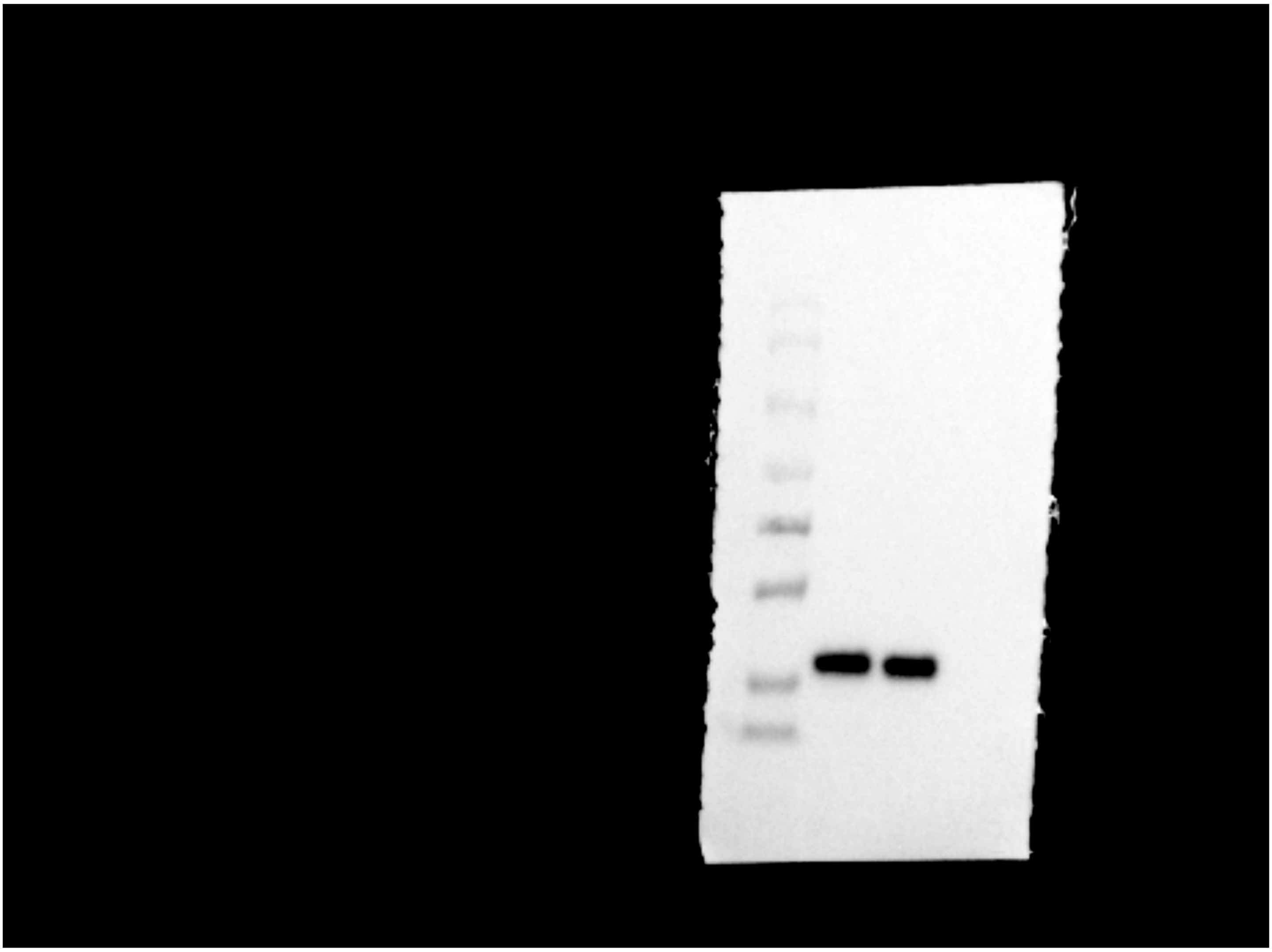

Supplementary Figure S5C source image 1

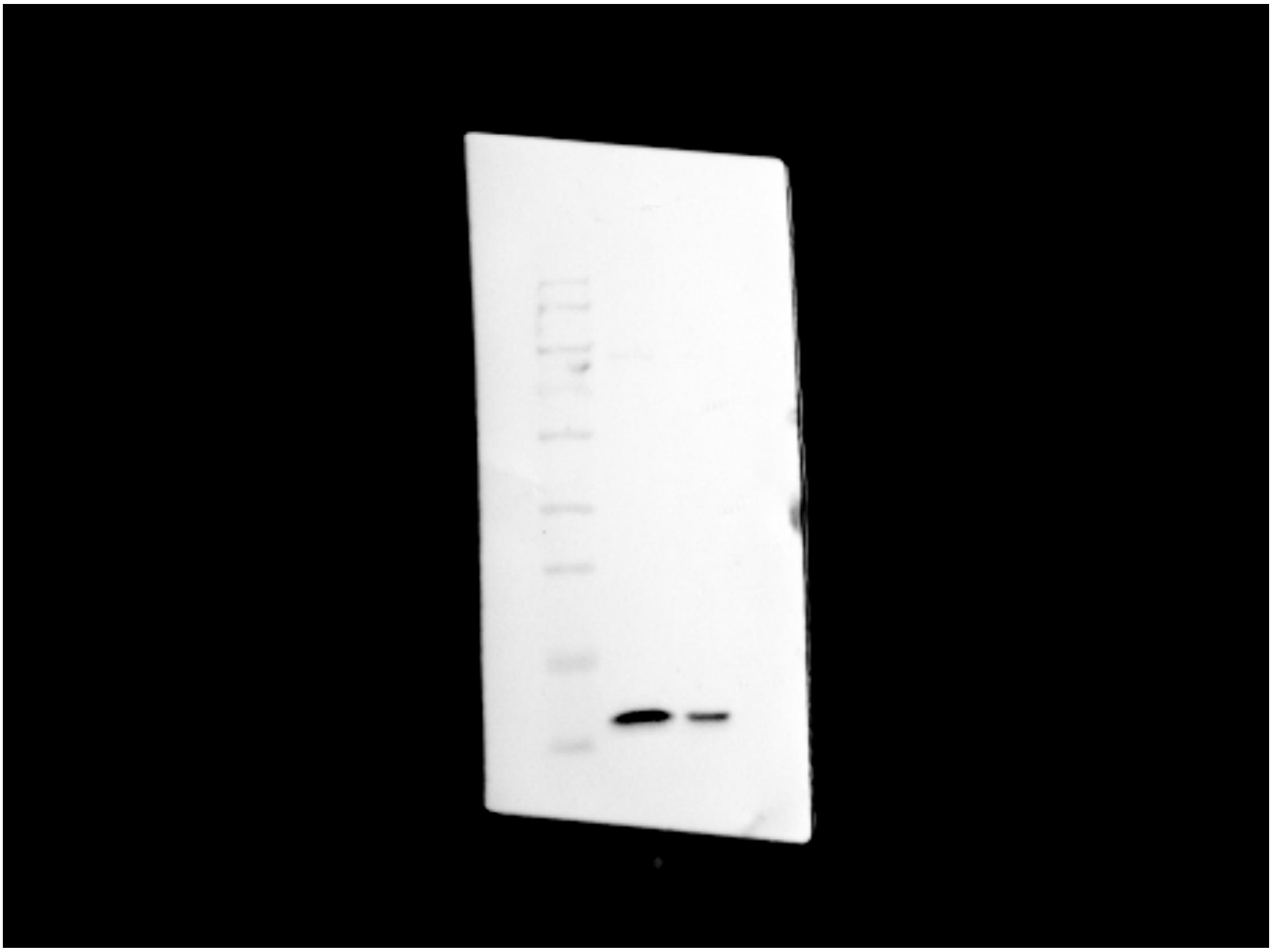

Supplementary Figure S5C source image 2

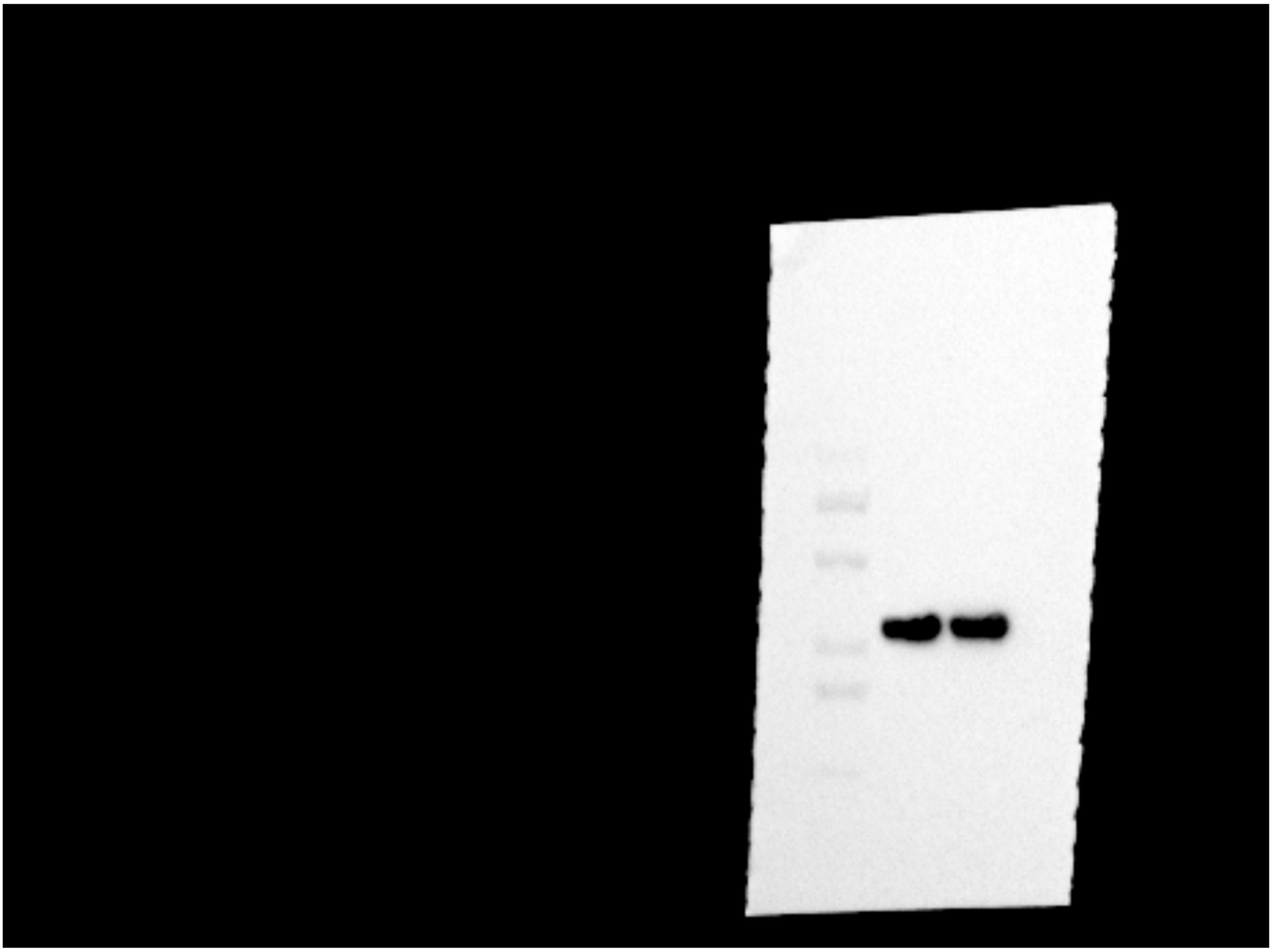

Supplement: Supplementary file 2 [file 12951_2026_4918_MOESM3_ESM.pdf]
