# Supplementary material for: MHY@MO@PPP inhibits ferroptosis positive feedback and delays the acute kidney injury-to-chronic kidney disease transition by remodeling the Fe2+-mTORC1-GPX4 axis
Source: J Nanobiotechnology. 2026 Aug 20;24:914. doi: 10.1186/s12951-026-04918-w (PMC13625235; doi:10.1186/s12951-026-04918-w)
Supplement: Supplementary file 3 [file 12951_2026_4918_MOESM1_ESM.docx]

**Table S1. shRNA sequences.**

| **Name** | **Sequence** |
| --- | --- |
| sh-GPX4#1 | 5’-GTGAGGCAAGACCGAAGTAAA-3’ |
| sh-GPX4#2 | 5’-GTGGATGAAGATCCAACCCAA-3’ |
| sh-NC | 5’-TTCTCCGAACGTGTCACGT-3’ |

**Table S2. RT-qPCR primer sequences (human).**

| **Gene** | **Primer Sequence** |
| --- | --- |
| GAPDH | F: 5'-CGGATTTGGTCGTATTGGGC-3' |
|  | R: 5'-TTGACGGTGCCATGGAATTTG-3' |
| KIM1 (HAVCR1) | F: 5'- TGGCAGATTCTGTAGCTGGTT -3' |
|  | R: 5'- AGAGAACATGAGCCTCTATTCCA -3' |
| NGAL (LCN2) | F: 5'- CCACCTCAGACCTGATCCCA -3' |
|  | R: 5'- CCCCTGGAATTGGTTGTCCTG -3' |
| CYSC (CST3) | F: 5'- GTCGGCGAGTACAACAAAGC -3' |
|  | R: 5'- CACCCCAGCTACGATCTGC -3' |
| ACTA2 | F: 5'- AAAAGACAGCTACGTGGGTGA -3' |
|  | R: 5'- GCCATGTTCTATCGGGTACTTC -3' |
| Fn1 | F: 5'- CGGTGGCTGTCAGTCAAAG -3' |
|  | R: 5'- AAACCTCGGCTTCCTCCATAA -3' |
| VIMENTIN (VIM) | F: 5'- CGGTGGCTGTCAGTCAAAG -3' |
|  | R: 5'- CTTTGTCGTTGGTTAGCTGGT -3' |

Note: F: Forward; R: Reverse.

**Table S3. Comparison of MHY@MO@PPP with representative nanozyme-based strategies reported for AKI-related oxidative injury.**

| System/material | Disease context/model | Main therapeutic emphasis | Key endpoints reported or emphasized | Difference from MHY@MO@PPP | Reference |
| --- | --- | --- | --- | --- | --- |
| Valence-engineered MoOx nanozyme | Experimental AKI | ROS scavenging through valence/catalysis-selectivity regulation | Renal oxidative stress, renal injury, and post-cure assessment | Focuses on MoOx catalytic regulation; does not incorporate MHY1485-mediated mTORC1/GPX4 intervention | [12] |
| Manganese-based nanozyme | Experimental AKI | Antioxidant and anti-inflammatory regulation involving Nrf2/HO-1 and PI3K/Akt/NF-κB signaling | Renal function, oxidative stress, inflammatory signaling, and tissue injury | Focuses on manganese-based redox regulation rather than Fe2+-mTORC1-GPX4 ferroptosis-axis remodeling | [15] |
| Gold nanozyme | Experimental AKI | Broad-spectrum antioxidant and anti-inflammatory activity | ROS/inflammation reduction and kidney-protective effects | Emphasizes clinically translatable antioxidant nanozyme activity without pH-responsive MHY1485 delivery | [16] |
| Fe-flavonoid nanozyme | Experimental AKI | Natural antioxidant/metal-organic nanozyme modulation of oxidative injury | ROS-related injury alleviation and renal protection | Represents antioxidant nanozyme therapy but does not directly test the MHY1485-mTORC1-GPX4 module | [17] |
| MHY@MO@PPP (this study) | UIRI-induced AKI-to-CKD transition and HK-2 ferroptosis model | Integrated ROS scavenging, responsive release, mTORC1 activation, and GPX4 restoration | Fe2+ burden, ROS/MDA/GSH, p-mTOR/GPX4/FTH1, renal function, fibrosis, and inflammation markers | Designed to target the ferroptosis-associated Fe2+-mTORC1-GPX4 network and fibrosis progression; component-specific in vivo attribution remains a limitation | This study |

Note: This comparison is intended to position the present platform relative to representative antioxidant nanozyme strategies. Efficacy endpoints are summarized qualitatively because disease models, doses, and follow-up durations differ across studies.
